# First-Trimester Antibiotic Use for Urinary Tract Infection and Risk of Congenital Malformations

**DOI:** 10.1001/jamanetworkopen.2025.19544

**Published:** 2025-07-09

**Authors:** Sarah S. Osmundson, Katelin B. Nickel, Susan M. Shortreed, Sascha Dublin, Dustin Stwalley, Michael J. Durkin, Paige D. Wartko, John M. Sahrmann, Ryan Colvin, Anne M. Butler

**Affiliations:** 1 Division of Maternal Fetal Medicine, Department of Obstetrics and Gynecology, Vanderbilt University Medical Center, Nashville, Tennessee; 2 Division of Infectious Diseases, John T. Milliken Department of Medicine, Washington University School of Medicine, St Louis, Missouri; 3 Kaiser Permanente Washington Health Research Institute, Seattle; 4 Department of Biostatistics, University of Washington, Seattle; 5 Department of Epidemiology, University of Washington, Seattle; 6 Institute for Informatics, Washington University School of Medicine, St Louis, Missouri; 7 Department of Surgery, Division of Public Health Sciences, Washington University School of Medicine, St Louis, Missouri

## Abstract

**Importance:**

Clinical guidelines recommend screening and treating bacteriuria in early pregnancy given that urinary tract infections (UTIs) can cause serious maternal and neonatal consequences. Evidence regarding antibiotic exposure during early pregnancy and risk of congenital malformations is limited and inconsistent.

**Objective:**

To compare the risk of congenital malformations following first-trimester exposure to different antibiotic agents used to treat UTI.

**Design, Setting, and Participants:**

This population-based cohort study included commercially insured pregnant individuals aged 15 to 49 years who were treated for UTI and linked liveborn infants in the Merative MarketScan Commercial Database (2006-2022).

**Exposure:**

First-trimester antibiotic prescription fill of nitrofurantoin, trimethoprim-sulfamethoxazole (TMP-SMX), fluoroquinolones (ciprofloxacin, levofloxacin, ofloxacin), and β-lactams to treat UTI.

**Main Outcomes and Measures:**

Congenital malformations (any and by organ system) were identified using validated algorithms based on diagnosis codes up to 365 days after birth. Log-binomial regression models were used to estimate propensity score–weighted risk ratios (RRs) and risk differences.

**Results:**

The cohort of 71 604 eligible pregnancies (median maternal [IQR] age, 30 [27-34] years) included 42 402 (59.2%) nitrofurantoin-exposed, 3494 (4.9%) TMP-SMX–exposed, 3663 (5.1%) fluoroquinolone-exposed, and 22 045 (30.8%) β-lactam–exposed individuals. Median (IQR) gestational age differed by antibiotic (nitrofurantoin, 62 [45-77] days; TMP-SMX, 26 [13-59] days; fluoroquinolones, 18 [9-27] days; β-lactams, 63 [48-77] days). The absolute risk of any malformation was 19.8 (95% CI, 18.0-21.8) per 1000 infants for β-lactams, 21.2 (95% CI, 19.9-22.7) per 1000 infants for nitrofurantoin, 23.5 (95% CI, 18.8-28.9) per 1000 infants for fluoroquinolones, and 26.9 (95% CI, 21.8-32.8) per 1000 infants for TMP-SMX. After accounting for confounding, risk of any congenital malformation was higher for TMP-SMX (RR, 1.35; 95% CI, 1.04-1.75) but similar for nitrofurantoin (RR, 1.12; 95% CI, 1.00-1.26) and fluoroquinolones (RR, 1.18; 95% CI, 0.87-1.60) compared with β-lactams. TMP-SMX was associated with increased risk of severe cardiac malformations (RR, 2.09; 95% CI, 1.09-3.99), other cardiac malformations (RR, 1.52; 95% CI, 1.02-2.25), and cleft lip and palate (RR, 3.23; 95% CI, 1.44-7.22) compared with β-lactams; however, for these specific malformations, the corresponding risk difference estimates included the null. Risk of other malformation types did not differ by agent, although some estimates were imprecise. Results were generally consistent across sensitivity analyses.

**Conclusions and Relevance:**

In this cohort study of first-trimester antibiotic exposure, the risk of any malformation, severe cardiac malformation, other cardiac malformation, and cleft lip and palate was higher for infants exposed to TMP-SMX vs β-lactam antibiotics. No elevated risk was observed for nitrofurantoin.

## Key Points

**Question:**

Are nitrofurantoin, trimethoprim-sulfamethoxazole (TMP-SMX), or fluoroquinolones associated with a higher risk of congenital malformations than β-lactams when used to treat urinary tract infection (UTI) during pregnancy?

**Findings:**

In this cohort study of 71 604 pregnancies with first-trimester antibiotic exposure for UTI, the risk of any malformation, severe cardiac malformations, other cardiac malformations, and cleft lip and palate was higher for TMP-SMX vs β-lactams and similar for nitrofurantoin and fluoroquinolones vs β-lactams, after accounting for confounding.

**Meaning:**

This study suggests that TMP-SMX may be associated with increased risk of malformations; however, no elevated risk was observed for nitrofurantoin.

## Teaser

This cohort study evaluates whether taking specific antibiotics (nitrofurantoin, fluoroquinolones, trimethoprim-sulfamethoxazole, and β-lactams) to treat a urinary tract infection (UTI) during the first trimester of pregnancy is associated with risk of congenital malformation.

## Introduction

Urinary tract infections (UTIs) are among the most common infections in pregnancy.^1,2,3^ They include asymptomatic bacteriuria (ASB; presence of bacteria in the urine without symptoms) and acute cystitis.^4^ ASB and acute cystitis are associated with adverse perinatal outcomes, including preterm birth, low birth weight, pyelonephritis, and maternal sepsis.^5,6^ Routine screening for ASB is recommended at the initial prenatal visit and often leads to antibiotic treatment in the first trimester of pregnancy—a period when the developing fetus is most susceptible to both teratogenic medication effects and potential adverse effects from infections.^7,8^

The American College of Obstetricians and Gynecologists (ACOG) provides vague guidance regarding antibiotic selection for the treatment of UTI in the first trimester due to uncertainty about the risk of congenital malformations.^4^ Concerns arise from several studies suggesting increased risk of congenital malformations associated with 2 antibiotics commonly used for UTI: nitrofurantoin and trimethoprim-sulfamethoxazole (TMP-SMX).^9,10,11,12,13^ However, these studies have important methodological limitations, such as self-report of antibiotic exposure and failure to account for important confounders. Many antibiotics cross the placenta, albeit in limited concentrations, with potential to impact fetal development.^14,15^ For example, TMP and SMX can inhibit folic acid metabolism and interfere with rapidly growing tissues.^16^ Despite the ACOG suggestion that nitrofurantoin and TMP-SMX be avoided during the first trimester when possible, they account for more than half of first-trimester UTI prescriptions.^17^

Evidence regarding antibiotic safety in pregnancy is needed to guide clinical practice. We evaluated whether nitrofurantoin, TMP-SMX, or fluoroquinolones were associated with congenital malformations (any and by organ system) compared with other antibiotics commonly used to treat UTIs in a commercially insured population in the United States.

## Methods

### Data Source

The Merative MarketScan Commercial Database contains longitudinal, patient-level data on enrollment, adjudicated inpatient and outpatient medical claims, and outpatient pharmacy-dispensed medications for individuals with employer-sponsored commercial insurance and their covered spouses and dependents.^18^ This study was approved by the Washington University School of Medicine institutional review board with a waiver of consent per section 164.512(i) of the Privacy Rule. We followed the Strengthening the Reporting of Observational Studies in Epidemiology (STROBE) reporting guideline for cohort studies.

### Study Design and Population

We identified a cohort of pregnant individuals who received first-trimester antibiotic therapy for UTI and their live-born infants. Pregnancies were identified via pregnancy outcome diagnosis and procedure codes (eTable 1 in Supplement 1 and eTable 23 in Supplement 2) from 2006 to 2022 among individuals ages 15 to 49 years (eMethods in Supplement 1).^19,20^ Several algorithms were applied to adjudicate pregnancy outcome type and determine the last menstrual period (LMP) date (eMethods and eTables 2-3 in Supplement 1 and eTables 24-25 in Supplement 2).^21,22,23^ We required pregnant individuals to have medical and prescription drug coverage from 3 months before LMP through 1 month after delivery. We required a first-trimester (defined as LMP to day 97 of pregnancy) outpatient pharmacy dispensing for an oral antibiotic agent commonly used to treat UTIs; the index date was defined as date of first antibiotic fill (eTable 4 in Supplement 1). We required a UTI indication within 7 days of the index date, defined as the presence of any of 3 criteria using outpatient claims: (1) diagnosis code for UTI (including ASB); (2) diagnosis code for UTI-related symptoms (ie, dysuria, painful micturition); or (3) procedure codes for a urine culture and antimicrobial susceptibility test (eTable 5 in Supplement 1 and eTable 26 in Supplement 2). Although laboratory results were not available, we considered a susceptibility test to be a proxy for a positive culture, since it is performed only when urine culture is positive.

We implemented several exclusion criteria (eTables 4 and 6 in Supplement 1 and eTables 27-28 in Supplement 2). To facilitate assessment of malformations coded on infant claims, we required pregnancy to end in a liveborn singleton delivery and infant linkage to the birthing parent’s insurance (eMethods in Supplement 1). We excluded pregnancies with more than 1 UTI-related antibiotic in the first trimester (>1 oral antibiotic class or ≥1 intramuscular or intravenous antibiotic), an index prescription days’ supply greater than 14 days, or an infection-related hospitalization. We excluded pregnancies in individuals who were immunosuppressed or with a spinal cord injury during the baseline period, defined as 90 days before LMP through the index date for medications and 180 days before LMP through the index date for diagnoses; pregnancies with diagnoses of chromosomal abnormalities; and pregnancies with first-trimester exposure to a known teratogenic medication.^24^

### Antibiotic Exposure

We used outpatient pharmacy claims to identify dispensings for oral antibiotic agents recommended for treatment of uncomplicated UTIs based on ACOG and Infectious Diseases Society of America guidelines: nitrofurantoin, TMP-SMX, fluoroquinolones (ciprofloxacin, levofloxacin, ofloxacin), and β-lactams (including cephalosporins and penicillins).^25,26^ eTable 4 in Supplement 1 presents antibiotic generic names. Pregnancies were considered exposed if the prescription was filled during the first trimester. β-Lactams served as the reference group because these agents are considered safe during pregnancy.^4^

### Outcomes

Congenital malformations (any and by organ system) were identified using validated algorithms developed by Kharbanda et al^27,28^ with high positive predictive values. The Kharbanda algorithms used infant electronic health record data to capture death and diagnosis codes, whereas we used inpatient and outpatient diagnosis codes on birthing parent claims for the first month after delivery and infant claims up to 365 days after birth. Because our data source did not include death, we were not able to implement one criterion: a single malformation diagnosis code and infant death in the first year. We made other slight modifications to specific malformation groups to align across the 2 versions of the algorithm (eTable 7 in Supplement 1 and eTable 29 in Supplement 2). Malformations were defined for each organ system–specific malformation group following the Kharbanda algorithms; most algorithms require a diagnosis code on more than 1 date (or 1 inpatient diagnosis, for some organ system categories).

### Covariates

Potential confounders were selected a priori based on expert opinion, including demographic characteristics, comorbidities, concomitant medications, and measures of health care utilization (eTable 8 in Supplement 1 and eTables 30-31 in Supplement 2).^29,30,31^ Demographic characteristics captured at index included age, geographic region, urban vs rural residence, and calendar year. Comorbidities (eg, pregestational diabetes or insulin use, chronic hypertension, obesity, alcohol use disorder) were captured at baseline. Suspected teratogenic medications were ascertained based on days’ supply overlapping the first trimester.^29^ Nonindex antibiotics were ascertained during the first trimester. Measures of prepregnancy health care utilization included hospitalization, emergency department visit, and number of outpatient office visits from 90 days before LMP through LMP.

### Statistical Analysis

For each malformation outcome, we reported the number of malformations and unadjusted absolute risks per 1000 infants with corresponding 95% CIs.^32^ To examine the association between antibiotic agents and risk of malformations, we used log-binomial regression models to estimate unadjusted and propensity score–weighted risk differences (RDs) and risk ratios (RRs).^33^ To account for confounding, 3 separate propensity scores were fit using logistic regression, estimating the probability that an individual initiated β-lactams vs each other antibiotic agent of interest (nitrofurantoin, TMP-SMX, fluoroquinolones), accounting for baseline covariates (eTable 8 in Supplement 1 and eTables 30-31 in Supplement 2). Propensity scores were used to calculate standardized mortality ratio (SMR) weights, which standardize the covariate distribution in each comparator cohort to the covariate distribution in the reference cohort (ie, β-lactams).^34^ We calculated and plotted the absolute standardized mean differences of baseline covariates in the unweighted and SMR-weighted samples to confirm weighting reduced imbalances of observed covariates. We applied asymmetric trimming of the propensity score as needed to remove pregnancies with nonoverlapping propensity scores.^35^ We used robust sandwich variance estimators to calculate SEs, accounting for weights and correlated multiple pregnancies in the same individual.^36,37^ To quantify the magnitude of the exposure-outcome impact, we calculated the number needed to harm (NNH) as the inverse of the RD estimate.

#### Sensitivity Analyses

We performed several sensitivity analyses to assess the robustness of our findings. We narrowed the β-lactam reference group to amoxicillin alone or cephalexin given that these agents are considered the safest antibiotics during pregnancy.^4^ We used an alternative outcome definition for malformations that required only a single diagnosis code. To mitigate confounding by severity, we restricted the cohort to symptomatic UTI (ie, excluding ASB), defined as meeting at least 1 of the following criteria: (1) urine culture on same date as antibiotic (eTable 5 in Supplement 1 and eTable 26 in Supplement 2); (2) no urine culture before or after 7 days of antibiotic; or (3) a UTI symptom diagnosis code before or after 7 days of antibiotic (eTable 5 in Supplement 1 and eTable 26 in Supplement 2). We restricted the cohort to deliveries in the *International Statistical Classification of Diseases, Tenth Revision, Clinical Modification (ICD-10-CM) *era, when coding for gestational age improved. We accounted for gestational age at index via cohort restriction. Specifically, we (1) narrowed the antibiotic exposure window to 2 to 13 weeks after LMP to exclude the preconception period and (2) attempted to narrow the antibiotic exposure window to the most critical period of organogenesis: 4 to 9 weeks after LMP for cardiac malformations^8^ and 7 to 10 weeks after LMP for cleft lip and cleft palate.^40^ We also accounted for gestational age at index via adjustment in the propensity score model. We calculated E-values to quantify the strength of unmeasured confounding needed to negate the observed associations.^41,42^ Finally, we explored the potential impact of selection bias^43^ related to restriction of the cohort to live births, using methods from Huybrechts et al^44^; we additionally used empirical data from our cohort on the distribution of pregnancy outcomes by antibiotic agent and examined scenarios where selection bias acted in opposite directions (eAppendix, eTable 22, and eFigures 16-17 in Supplement 1).

#### Alternative Analyses

We conducted analyses of the association between antibiotic agents and the risk of any and cardiac malformations in a larger cohort of pregnant individuals treated with a UTI-related antibiotic for any indication (eTable 5 in Supplement 1 and eTable 26 in Supplement 2).

Analyses were conducted using SAS Software version 9.4 (SAS Institute Inc) and R version 4.3 (R Project for Statistical Computing). Our approach to interpreting results was based on an evaluation of the magnitude, direction, and precision of the effect estimates.^38,39^

## Results

We identified 71 604 pregnancies that met study eligibility criteria (eFigure 1 and eTables 9-10 in Supplement 1). The cohort included 42 402 individuals (59.2%) with first-trimester exposure to nitrofurantoin; 3494 (4.9%) with first-trimester exposure to TMP-SMX; 3663 (5.1%) with first-trimester exposure to fluoroquinolones; and 22 045 (30.8%) with first-trimester exposure to β-lactams. The distribution of agents within antibiotic class and over time is presented in eTable 11 and eFigure 2 in Supplement 1.

**Figure 1.**
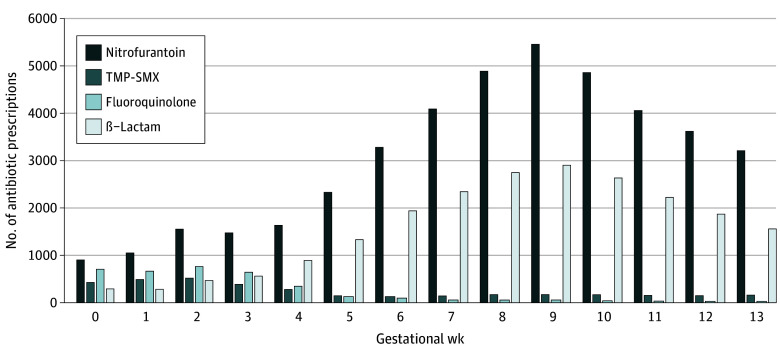
Utilization of Antibiotic Therapy for Urinary Tract Infection (UTI) During First Trimester of Pregnancy, by Agent and Gestational Week The median (IQR) gestational age at index for symptomatic UTI was 6 (3-9) weeks compared with 9 (7-11) weeks for asymptomatic bacteriuria. TMP-SMX indicates trimethoprim-sulfamethoxazole.

Pregnant individuals had a median (IQR) age of 30 (27-34 years), and most (69 639 [98.6%]) contributed a single pregnancy. Patient characteristics were generally similar across agents, with few exceptions (Table; eFigure 3 in Supplement 1).^45^ Median (IQR) gestational age at index was older for those receiving nitrofurantoin (62 [45-77] days) and β-lactams (63 [48-77] days) vs TMP-SMX (26 [13-59] days) and fluoroquinolones (18 [9-27] days) (Figure 1). All measured patient characteristics were well-balanced between exposure groups after propensity score weighting in all primary analyses, as indicated by standardized mean differences less than 0.10 (eTable 12 and eFigure 3 in Supplement 1).

**Table.** Characteristics of 71 604 Pregnancies Among Individuals Treated for Urinary Tract Infection in the First Trimester by Index Antibiotic Agent

Characteristic	Pregnancies, No. (%)			
	Nitrofurantoin	TMP-SMX	Fluoroquinolone	β-lactam
No. of pregnancies	42 402	3494	3663	22 045
No. of birthing parents	41 965	3489	3661	21 928
Age of birthing parent at index, y^a^				
15-22	1727 (4.1)	192 (5.5)	171 (4.7)	814 (3.7)
23-29	16 512 (38.9)	1462 (41.8)	1469 (40.1)	8073 (36.6)
30-34	15 480 (36.5)	1155 (33.1)	1302 (35.5)	8208 (37.2)
35-39	7227 (17.0)	564 (16.1)	604 (16.5)	4141 (18.8)
40-49	1456 (3.4)	121 (3.5)	117 (3.2)	809 (3.7)
Region of residence				
Northeast	5695 (13.4)	454 (13.0)	460 (12.6)	3353 (15.2)
Midwest	8674 (20.5)	896 (25.6)	785 (21.4)	5053 (22.9)
South	19 680 (46.4)	1537 (44.0)	1666 (45.5)	8837 (40.1)
West	7965 (18.8)	575 (16.5)	704 (19.2)	4648 (21.1)
Unknown	388 (0.9)	32 (0.9)	48 (1.3)	154 (0.7)
Urbanicity				
Urban	36 620 (86.4)	2891 (82.7)	3136 (85.6)	18 549 (84.1)
Rural	4165 (9.8)	504 (14.4)	413 (11.3)	2354 (10.7)
Missing	1617 (3.8)	99 (2.8)	114 (3.1)	1142 (5.2)
Year of index antibiotic fill^a^				
2006-2010	12 612 (29.7)	1339 (38.3)	1452 (39.6)	4710 (21.4)
2011-2016	18 635 (44.0)	1493 (42.7)	1722 (47.0)	8500 (38.6)
2017-2022	11 155 (26.3)	662 (19.0)	489 (13.3)	8835 (40.1)
Gestational age of index antibiotic fill, wk				
0-2	3505 (8.3)	1436 (41.1)	2138 (58.4)	1043 (4.7)
3-5	5441 (12.8)	813 (23.3)	1120 (30.6)	2784 (12.6)
6-8	12 258 (28.9)	442 (12.7)	212 (5.8)	7030 (31.9)
9-11	14 371 (33.9)	493 (14.1)	132 (3.6)	7761 (35.2)
12-13	6827 (16.1)	310 (8.9)	61 (1.7)	3427 (15.6)
Comorbidities^b^				
Pregestational diabetes or insulin use	628 (1.5)	47 (1.4)	63 (1.7)	352 (1.6)
Chronic heart disease	9 (0.0)	3 (0.1)	2 (0.0)	9 (0.0)
Chronic hypertension	1226 (2.9)	91 (2.6)	98 (2.7)	706 (3.2)
Obesity	1773 (4.2)	138 (4.0)	118 (3.2)	1199 (5.4)
Pelvic inflammatory disease or sexually transmitted infection	6388 (15.1)	310 (8.9)	303 (8.3)	3076 (13.9)
Alcohol use disorder	71 (0.2)	2 (0.1)	8 (0.2)	34 (0.1)
Other substance use disorder	93 (0.2)	11 (0.3)	13 (0.3)	64 (0.3)
Tobacco use	866 (2.0)	89 (2.5)	60 (1.6)	538 (2.4)
Suspected teratogenic medication^c^				
Benzodiazepine	991 (2.3)	86 (2.5)	126 (3.4)	554 (2.5)
Fluconazole^d^	334 (0.8)	79 (2.3)	115 (3.1)	142 (0.6)
Folic acid antagonist	275 (0.7)	24 (0.7)	33 (0.9)	155 (0.7)
Selective serotonin reuptake inhibitor	2670 (6.3)	237 (6.8)	278 (7.6)	1602 (7.3)
Other suspected teratogen^e^	424 (1.0)	39 (1.1)	50 (1.4)	215 (1.0)
Other medications				
Nonindex antibiotic^f^	4258 (10.0)	400 (11.4)	462 (12.6)	2327 (10.6)
Oral antidiabetic^b^	1422 (3.4)	106 (3.0)	116 (3.2)	761 (3.5)
Prepregnancy health care utilization intensity^g^				
Any hospitalization	328 (0.8)	29 (0.8)	32 (0.9)	177 (0.8)
Any emergency department visit	2725 (6.4)	238 (6.8)	264 (7.2)	1447 (6.6)
No. of outpatient office visits, mean (SD)^a^	1.2 (1.7)	1.2 (1.6)	1.3 (1.6)	1.3 (1.7)

Abbreviation: TMP-SMX, trimethoprim-sulfamethoxazole.

^a^ Included in the propensity score model as a continuous covariate using a restricted cubic spline.^45^

^b^ Identified during the baseline period. For diagnosis codes, this was defined as 180 days before the last menstrual period date through the index antibiotic date. Baseline period for medications was defined as 90 days before the last menstrual period date through the index antibiotic date.

^c^ Identified as days’ supply overlapping the first trimester.

^d^ A total of 12 000 mg of fluconazole exposure during the exposure period was required based on prescription days’ supply, dose, and quantity.

^e^ Other suspected teratogenic medications were aminoglycoside, angiotensin-converting enzyme inhibitor, angiotensin receptor blocker, danazol, methimazole, molnupiravir, mycophenolate, potassium iodide, propylthiouracil, ribavirin, statin, and tetracycline.

^f^ Identified during the first trimester.

^g^ Identified prepregnancy (ie, 90 days before last menstrual period through last menstrual period date).

The distribution of any and organ system-specific congenital malformations is presented in eTable 13 in Supplement 1. We observed 1518 infants with malformations, including 729 with cardiac malformations. The unadjusted absolute risk of any malformation by antibiotic agent was: 19.8 (95% CI, 18.0-21.8) per 1000 infants for β-lactams, 21.2 (95% CI, 19.9-22.7) per 1000 infants for nitrofurantoin, 23.5 (95% CI, 18.8-28.9) per 1000 infants for fluoroquinolones, and 26.9 (95% CI, 21.8-32.8) per 1000 infants for TMP-SMX; patterns were similar for any cardiac malformations (eTable 14 in Supplement 1).

**Figure 2.**
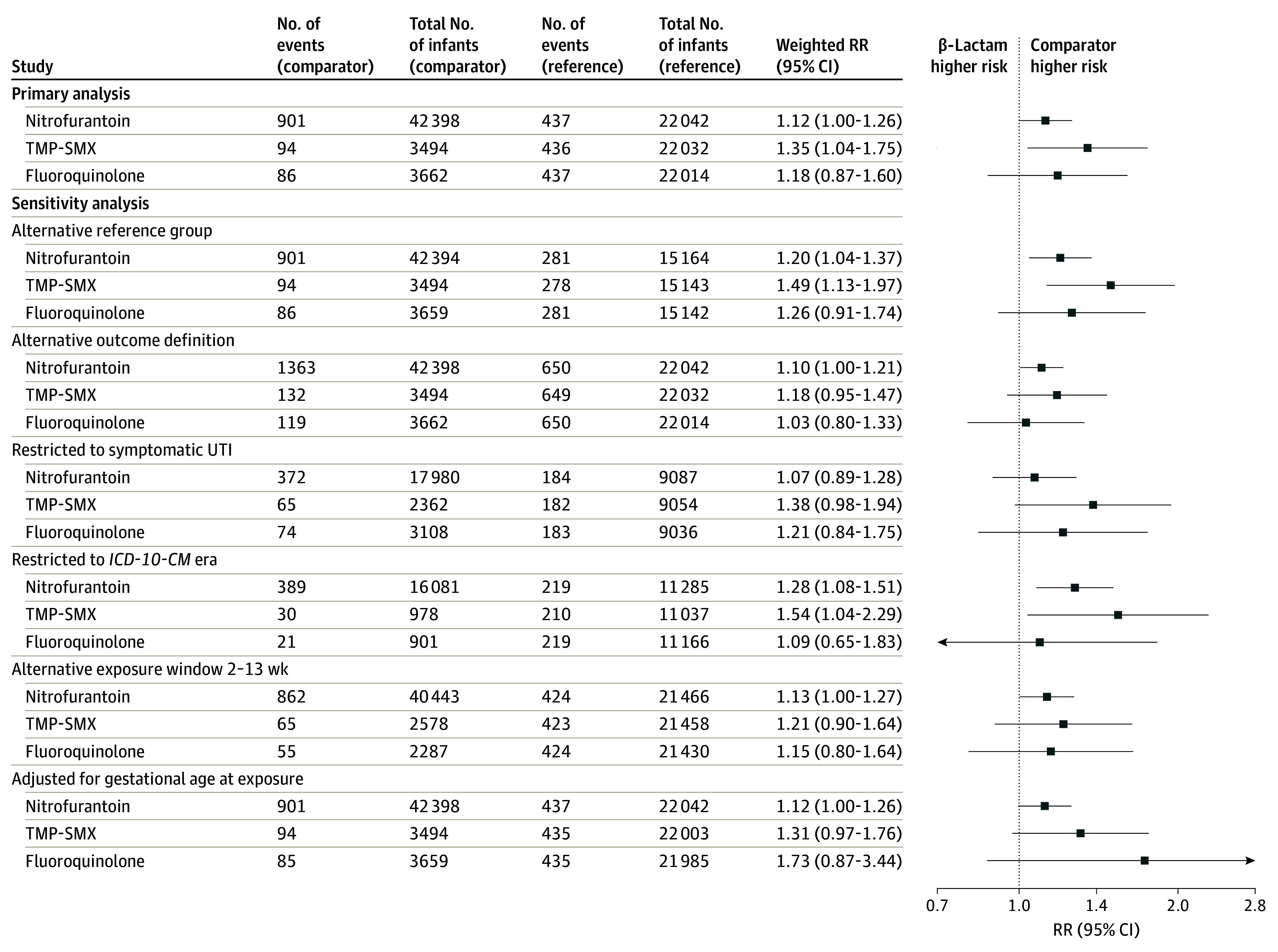
Weighted Risk Ratio (RR) Estimates of Any Congenital Malformation in Infants Born to Individuals Treated for Urinary Tract Infection (UTI) in the First Trimester Propensity score–weighted analysis accounted for all potential confounders listed in eTable 8 in Supplement 1 and eTables 30 and 31 in Supplement 2. At least 5 events in each exposure group were required to estimate the treatment effect. The reference group was the β-lactam group. Alternative reference group for sensitivity analysis was receipt of amoxicillin alone or cephalexin (rather than all UTI-related β-lactam antibiotics). Alternative outcome definition relaxed the congenital malformation algorithm to allow any single diagnosis code within the specific malformation group (after excluding diagnostic and rule-out codes [eMethods in Supplement 1]). Symptomatic UTI restricted to pregnancies with symptomatic UTIs (definition provided in Methods section). *International Statistical Classification of Diseases, Tenth Revision, Clinical Modification *(*ICD-10-CM*) era restricted to pregnancies with delivery dates on or after October 1, 2015. Alternative exposure window restricted the antibiotic exposure timeframe from 0 to 13 weeks’ to 2 to 13 weeks’ gestation (14-97 days’ gestation). Adjusted for gestational age at exposure used gestational age distribution tertiles (0-6, 7-9, and 10-13 weeks). TMP-SMX indicates trimethoprim-sulfamethoxazole.

The crude and weighted associations between each comparator antibiotic agent vs β-lactams and any malformation are presented in Figure 2 as well as eFigure 4 and eTable 15 in Supplement 1. Compared with β-lactam–exposed infants, TMP-SMX–exposed infants had a higher risk of any malformation on the relative scale (weighted RR, 1.35; 95% CI, 1.04 to 1.75) and absolute scale (weighted RD per 1000, 6.88; 95% CI, 0.11 to 13.65). The NNH suggested that 1 additional malformation would occur for every 145 (95% CI, 73 to 8826) TMP-SMX–exposed pregnancies. Compared with β-lactam–exposed infants, the risk of any malformation was similar for nitrofurantoin-exposed infants (weighted RR, 1.12; 95% CI, 1.00 to 1.26; weighted RD per 1000, 2.41; 95% CI, 0.00 to 4.82) and fluoroquinolone-exposed infants (weighted RR, 1.18; 95% CI, 0.87 to 1.60; weighted RD per 1000, 3.63; 95% CI, −3.43 to 10.70).

**Figure 3.**
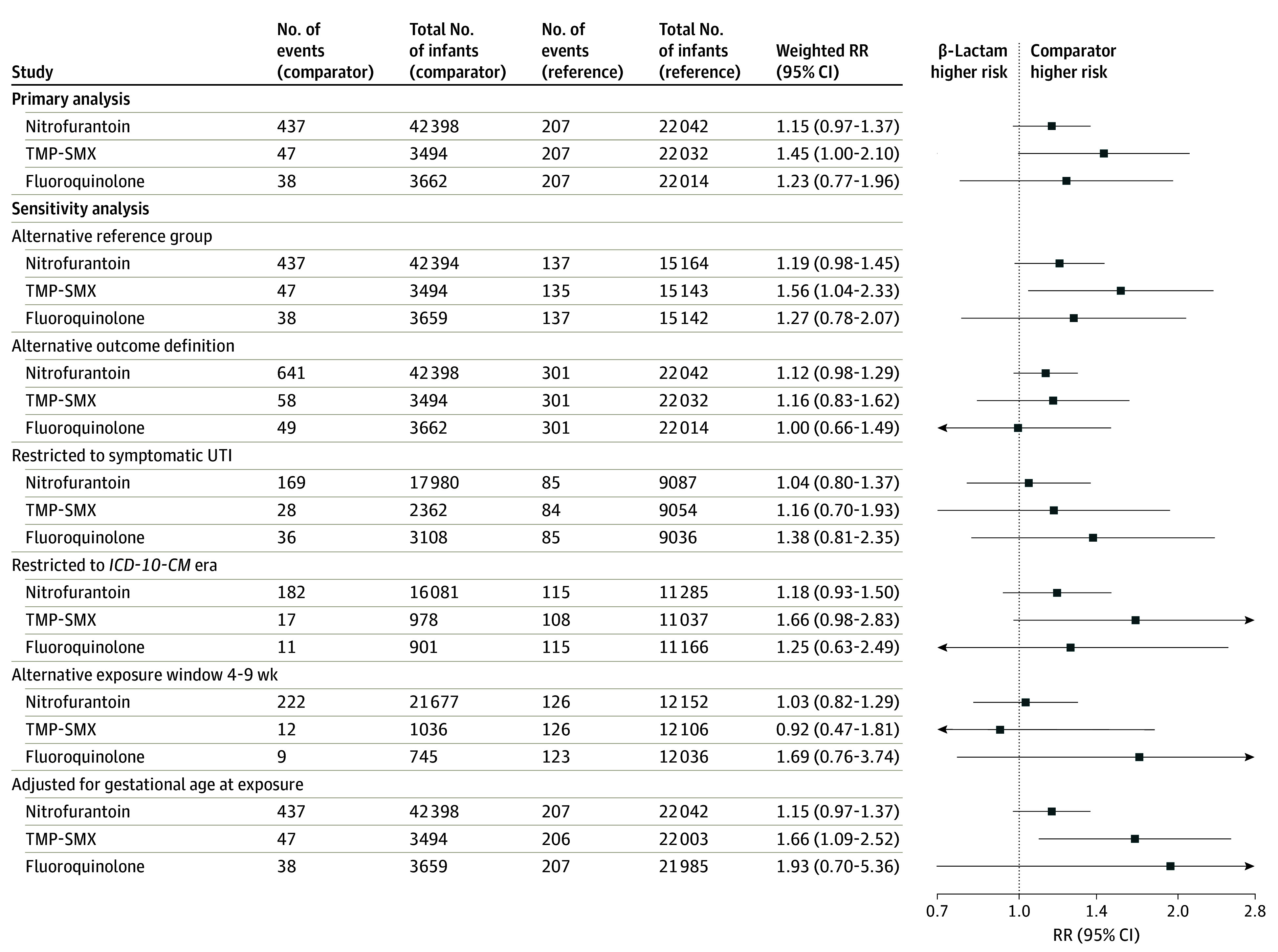
Weighted Risk Ratio (RR) Estimates of Any Cardiac Malformation in Infants Born to Individuals Treated for Urinary Tract Infection (UTI) in the First Trimester Propensity score–weighted analysis accounted for all potential confounders listed in eTable 8 in Supplement 1 and eTables 30 to 31 in Supplement 2. At least 5 events in each exposure group were required to estimate the treatment effect. The reference group was the β-lactam group. Alternative reference group for sensitivity analysis was receipt of amoxicillin alone or cephalexin (rather than all UTI-related β-lactam antibiotics). Alternative outcome definition relaxed the congenital malformation algorithm to allow any single diagnosis code within the specific malformation group (after excluding diagnostic and rule-out codes [eMethods in Supplement 1]). Symptomatic UTI restricted to pregnancies with symptomatic UTIs (definition provided in Methods section). *International Statistical Classification of Diseases, Tenth Revision, Clinical Modification *(*ICD-10-CM*) era restricted to pregnancies with delivery dates on or after October 1, 2015. Alternative exposure window restricted the antibiotic exposure timeframe from 0 to 13 weeks’ to 4 to 9 weeks’ gestation (28-69 days’ gestation). Adjusted for gestational age at exposure used gestational age distribution tertiles (0-6, 7-9, and 10-13 weeks). TMP-SMX indicates trimethoprim-sulfamethoxazole.

**Figure 4.**
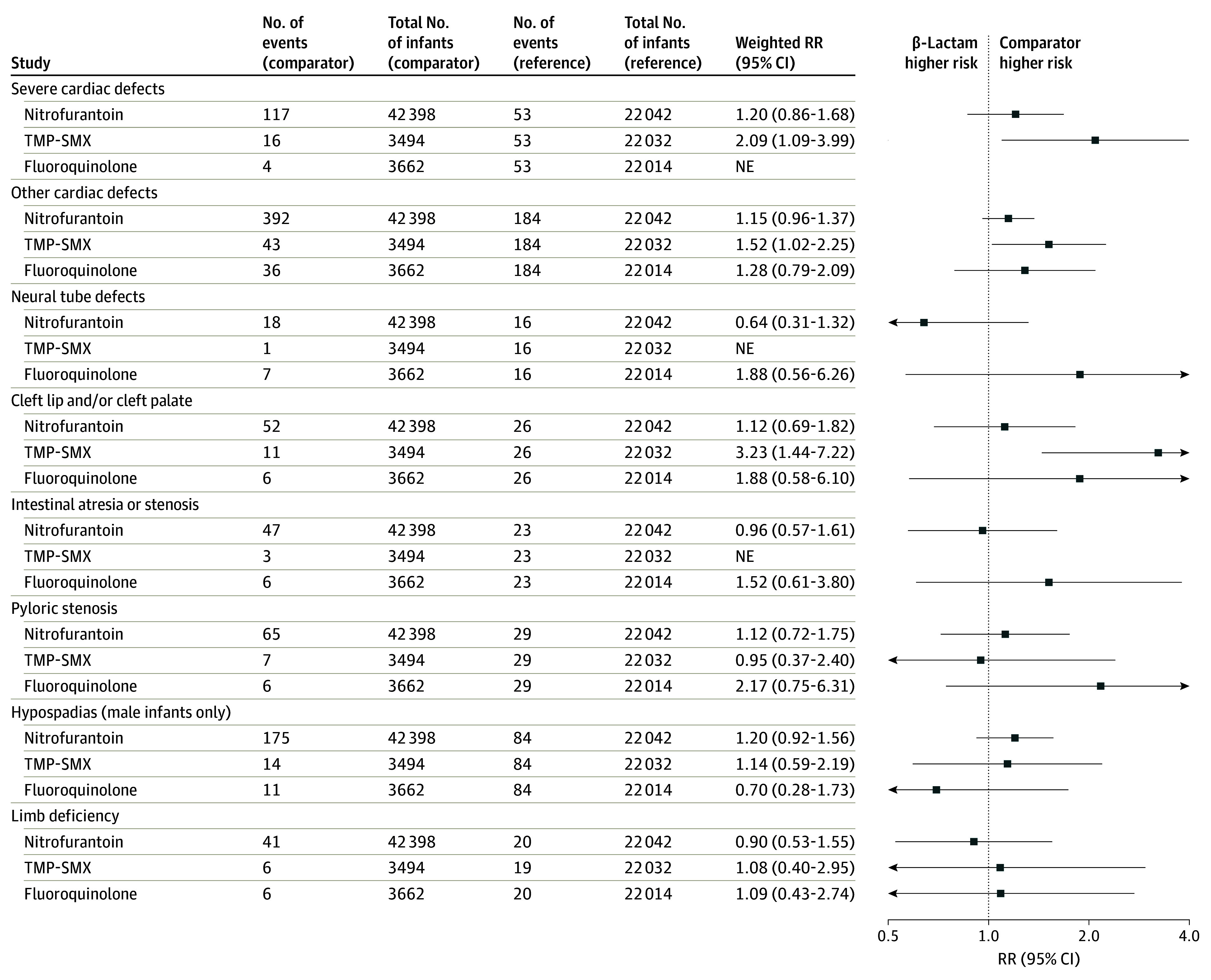
Weighted Risk Ratio (RR) Estimates of Specific Malformation Groups in Infants Born to Individuals Treated for Urinary Tract Infection in the First Trimester Propensity score–weighted analysis accounted for all potential confounders listed in eTable 8 in Supplement 1 and eTables 30-31 in Supplement 2. At least 50 events in the urinary tract infection cohort were required to perform specific malformation group analyses, and at least 5 events in each exposure group were required to estimate the treatment effect. NE indicates not estimable; TMP-SMX, trimethoprim-sulfamethoxazole.

In analyses of organ-specific malformation groups, risk was similar for TMP-SMX–exposed and β-lactam–exposed pregnancies for cardiac malformations (weighted RR 1.45, 95% CI 1.00 to 2.10; weighted RD per 1000, 4.20; 95% CI, −0.71 to 9.11) (Figure 3; eFigure 5 and eTable 16 in Supplement 1) and elevated for orofacial and respiratory malformations on a relative but not absolute scale (weighted RR, 2.89; 95% CI, 1.31 to 6.41; weighted RD per 1000, 2.49; 95% CI, −0.24 to 5.23) (eFigures 6-7 in Supplement 1). Analyses further restricted to specific malformation groups provided additional insight. Specifically, compared with β-lactam–exposed pregnancies, TMP-SMX–exposed pregnancies had a higher risk of severe cardiac malformations, other cardiac malformations, and cleft lip and cleft palate on a relative but not absolute scale (Figure 4; eFigure 8 in Supplement 1). For other organ-specific malformation groups (eFigure 6 and eTable 17 in Supplement 1) and other specific malformation groups (Figure 4; eTable 18 in Supplement 1), results did not suggest differential risk by antibiotic agent or were not estimable.

### Sensitivity Analyses

Sensitivity analyses using the alternative reference group, alternative outcome definition, restriction to symptomatic UTI (vs ASB), and adjustment for gestational age at index in the propensity score yielded effect estimates consistent with the magnitude and direction of estimates in primary analyses for any malformation (Figure 2; eFigure 4 in Supplement 1). For the nitrofurantoin and TMP-SMX vs β-lactam comparisons, restricting to *ICD-10-CM* era deliveries resulted in larger effect estimates further from the null, albeit less precise. For the TMP-SMX vs β-lactam comparison, narrowing the exposure window to 2 to 13 weeks of gestation resulted in less precise effect estimates that shifted down and included the null (weighted RR, 1.21; 95% CI, 0.90 to 1.64; weighted RD per 1000, 4.20; 95% CI, −2.86 to 11.27). Narrowing the antibiotic exposure window to the most critical organogenesis period for cardiac malformations (4-9 weeks’ gestation for any, severe, other) indicated no evidence of elevated risk with nitrofurantoin; effect estimates for TMP-SMX included the null but were imprecise (Figure 3; eFigures 5 and 9-10 in Supplement 1). Narrowing the antibiotic exposure window for cleft lip and cleft palate (7-10 weeks’ gestation) indicated no evidence of elevated risk with nitrofurantoin on the relative or absolute scale; effect estimates for TMP-SMX were not estimable (eFigures 9-10 in Supplement 1).

The E-value for RR estimates suggested that a potential confounder would need to have a large association with both the antibiotic exposure and malformation outcome (any malformations, 2.03; specific malformations, >2.40) beyond the measured covariates to explain the higher risk of malformations in the TMP-SMX vs β-lactam comparisons (eTable 19 in Supplement 1). Selection bias due to the restriction of the cohort to live births was unlikely to have impacted our findings (eAppendix in Supplement 1).

### Alternative Analyses

eTables 20 and 21 in Supplement 1 present patient characteristics of 256 686 pregnant individuals treated with a UTI-related antibiotic for any indication. Among TMP-SMX–exposed pregnancies, the absolute risk of any malformation was higher for UTI vs other indications; the risk did not vary by indication for other antibiotics (eFigure 11 in Supplement 1). Compared with β-lactam–exposed infants, the risk of any malformations and cardiac malformations was similar for nitrofurantoin-exposed, TMP-SMX–exposed, and fluoroquinolone–exposed infants; results of most sensitivity analyses were null (eFigures 12-15 in Supplement 1).

## Discussion

In this population-based cohort study of commercially insured pregnant individuals with UTI, first-trimester exposure to TMP-SMX was associated with increased risk of any congenital malformation (1 additional malformation per 145 pregnant individuals treated with TMP-SMX vs β-lactams). Results for specific malformations showed some inconsistencies; compared with β-lactams, TMP-SMX was associated with severe cardiac, other cardiac, and cleft lip and cleft palate malformations on the relative scale but not the absolute scale. Importantly, we did not observe increased risk of malformations associated with nitrofurantoin vs β-lactams, and findings were consistent and precise on both the relative and absolute scale. Results were consistent across several sensitivity analyses.

Existing evidence on the risk of malformations associated with first-trimester antibiotic therapy for UTI is limited. To our knowledge, our study is the first large-scale examination restricted to pregnant individuals with UTI. Previous studies—which include individuals treated for heterogeneous indications—reported mixed results.^9,10,12,13,46,47,48^ A meta-analysis found an association between first-trimester exposure to nitrofurantoin and major malformation across 3 case-control studies that measured exposure via self-report (odds ratio [OR], 1.22; 95% CI, 1.02-1.45), but no association across 5 cohort studies (RR, 1.01; 95% CI, 0.81-1.26).^10^ For all studies, the comparator group was unexposed pregnancies rather than an active comparator, which precluded the ability to properly control confounding by indication.^49^ In the largest cohort study of TMP-SMX, Hansen et al^47^ reported no difference in risk of several specific congenital malformations between 6688 infants with first-trimester exposure to TMP-SMX compared with 6688 with first-trimester exposure to β-lactams for any indication (eg, cardiac defects: odds ratio [OR], 0.94; 95% CI, 0.71-1.23). A population-based cohort study in Denmark reported increased risk of cleft lip for early gestational exposure to sulfamethoxazole (OR, 1.76; 95% CI, 1.10-2.81) and trimethoprim (OR, 14.29; 95% CI, 3.46-59.05) compared with unexposed pregnancies.^11^ Additional limitations of some prior studies include inclusion of individuals with exposure to known teratogens, failure to account for important potential confounders, (eg, suspected teratogens, comorbidities), lack of analysis of malformation subtypes, and limited statistical power.

Our study design addresses 2 major limitations of prior studies: heterogeneous antibiotic indications and unexposed comparator groups. To mitigate possible confounding by indication, we restricted the cohort to a single indication (uncomplicated UTI) and used an active comparator (β-lactams—widely accepted as safe in pregnancy).^49,50,51^ This cohort restriction is important because pregnant individuals receive antibiotics for a variety of non-UTI indications (eg, respiratory infections) that could independently increase risk of malformations.^52,53^ In contrast to our findings in the UTI cohort, the associations of TMP-SMX vs β-lactams with risk of malformations were null in the alternative analyses conducted in a larger cohort with a wide range of indications. Among TMP-SMX–exposed pregnancies, the absolute risk of any malformation was higher for pregnancies with UTI vs other indications, whereas risk did not vary by indication for other antibiotics. There is no obvious explanation for this finding, and it suggests that confounding by indication may not explain the difference in results between the UTI cohort and the broader cohort.

In our cohort, TMP-SMX use occurred much earlier in pregnancy than β-lactam use (median gestational age, 26 vs 63 days), and very little TMP-SMX use occurred at 10 to 13 weeks, when ASB screening typically occurs. It is possible that TMP-SMX–exposed individuals had more unrecognized or unplanned pregnancies than β-lactam–exposed individuals, which could result in residual confounding. Unrecognized or unplanned pregnancies may have more exposure to teratogenic prescription medications, tobacco, alcohol, or illicit drugs^54,55,56,57^—risk factors for malformations.^58,59,60,61^ In our propensity score–weighted cohort, TMP-SMX–exposed and β-lactam–exposed individuals had similar distributions of some measured confounders, such as tobacco use and suspected teratogenic medication use. Furthermore, median gestational age at prenatal care initiation did not differ by antibiotic type (range, 54-59 days). Because routine screening for ASB occurs late in the first trimester, patients prescribed antibiotics earlier in pregnancy may be more likely to have symptomatic UTI (which could theoretically convey higher risk of malformations than ASB). However, results did not change meaningfully after restriction to symptomatic UTI. Finally, our bias analyses indicated that unmeasured confounders would have to be strongly associated with the antibiotic and malformation to explain the observed associations. Thus, after careful exploration of potential biases, we have not found an alternative explanation for the observed association of TMP-SMX exposure with higher malformation risk.

### Limitations

This study has limitations. First, given the nonrandomized study design, results may be subject to residual confounding due to unmeasured or poorly measured factors. Some important covariates are not captured or undercaptured in administrative data, such as genetic factors, obesity, tobacco use, alcohol use, substance use, and severity of infection.^62^ Second, our study population was restricted to live births, which may introduce selection bias due to unobserved malformation outcomes if the probability of livebirth differs between pregnancies exposed to different antibiotic agents.^63^ Third, outcome misclassification is possible, as malformations were identified using claims-based algorithms rather than medical records. We expect such misclassification to be nondifferential by exposure group, which would typically bias findings toward the null.^64^ Fourth, exposure misclassification is possible if individuals did not consume filled prescriptions. Fifth, some analyses included a small number of events and produced imprecise estimates. Sixth, our study included commercially insured pregnancies, and results may not generalize to Medicaid-insured or uninsured populations.

## Conclusions

In this cohort study of pregnant individuals and their infants, first-trimester TMP-SMX exposure to treat UTI was associated with increased risk of any malformation, severe cardiac malformation, other cardiac malformation, and cleft lip and palate compared with β-lactam exposure. No elevated risk was observed for nitrofurantoin. Our results support the current ACOG recommendation for caution in using TMP-SMX during the first trimester but do not support current recommendations to limit nitrofurantoin use.

## Notes

Supplement 1.
eMethods.

eReferences.
**eTable 1.** Codes to Identify Pregnancy Outcomes **eTable 2.** Codes to Verify Pregnancy Outcome **eTable 3.** Codes to Determine Gestational Age **eTable 4.** Oral Antibiotic Categories for Agents Used to Treat Urinary Tract Infection **eTable 5.** Codes to Identify Possible Antibiotic Indication(s) ± 7 Days from the Index Antibiotic Date **eTable 6.** Codes to Identify Individuals for Exclusion **eTable 7.** Outcome Definitions for Congenital Malformations Based on Validated Algorithms by Kharbanda et al **eTable 8.** Codes to Identify Covariates **eTable 9.** Distribution of Urinary Tract Infection-related Oral Antibiotic Combinations that Resulted in Exclusion from the Study Cohort **eTable 10.** Distribution of UTI Indication Criterion Qualification among Individuals Treated for Urinary Tract Infection in the First Trimester of Pregnancy **eTable 11.** Distribution of Index Oral Antibiotic Agents among Individuals Treated for Urinary Tract Infection in the First Trimester of Pregnancy **eTable 12.** Characteristics of Pregnancies Among Individuals Treated for Urinary Tract Infection in the First Trimester by Index Antibiotic Agent, After Propensity Score Weighting **eTable 13.** Absolute Risk of Congenital Malformations Among Infants Born to Individuals Treated for Urinary tract infection in the First Trimester With an Antibiotic Agent by Organ-Specific Malformation Group and Outcome Definition **eTable 14.** Absolute Risk of Congenital Malformations Among Infants Born to Individuals Treated for Urinary Tract Infection in the First Trimester by Index Antibiotic Agent **eTable 15.** Crude and Weighted Risk Difference and Risk Ratio Estimates of Any Congenital Malformation in Infants Born to Individuals Treated for Urinary Tract Infection (UTI) in the First Trimester: Comparison of Different UTI-related Antibiotics Versus β-lactams (Reference) **eTable 16.** Crude and Weighted Risk Difference and Risk Ratio Estimates of Any Cardiac Malformation in Infants Born to Individuals Treated for Urinary Tract Infection (UTI) in the First Trimester: Comparison of Different UTI-related Antibiotics Versus β-lactams (Reference) **eTable 17.** Crude and Weighted Risk Difference and Risk Ratio Estimates of Other Organ-Specific Malformations in Infants Born to Individuals Treated for Urinary Tract Infection (UTI) in the First Trimester: Comparison of Different UTI-related Antibiotics Versus β-lactams (Reference) **eTable 18.** Crude and Weighted Risk Difference and Risk Ratio Estimates of Specific Malformation Groups in Infants Born to Individuals Treated for Urinary Tract Infection (UTI) in the First Trimester: Comparison of Different UTI-related Antibiotics Versus β-lactams (Reference) **eTable 19.** E-values and Associated Weighted Risk Ratio Estimates of Any Congenital Malformation, Any Cardiac Malformation, Other Organ-Specific Congenital Malformations, and Specific Congenital Malformations in Infants Born to Individuals Treated for Urinary Tract Infection (UTI) in the First Trimester: Comparison of Different UTI-related Antibiotics Versus β-lactams (Reference) **eTable 20.** Characteristics of Pregnancies Among Individuals Treated with a UTI-related Antibiotic for Any Indication in the First Trimester by Index Antibiotic Agent (N=275,413) **eTable 21.** Characteristics of Pregnancies Among Individuals Treated with a UTI-related Antibiotic for Any Indication in the First Trimester by Index Antibiotic Agent, After Propensity Score Weighting **eTable 22.** Potential Values of the Probability of Livebirth for β-lactam and Trimethoprim-sulfamethoxazole Under the Assumption of a Higher Probability of Livebirth for β-lactam and Corrected Risk Ratios for Any Malformation **eFigure 1.** Derivation of Cohort of Pregnant Individuals Treated for Urinary Tract Infection (UTI) in the First Trimester with an Antibiotic Agent in the MarketScan Commercial Database **eFigure 2.** Utilization of Antibiotic Agents for the Treatment of Urinary Tract Infection During the First Trimester of Pregnancy by Calendar Year **eFigure 3.** Standardized Mean Differences of Patient Characteristics Among Pregnant Individuals Treated for Urinary Tract Infection in the First Trimester Between Each Antibiotic Exposure Group versus β-lactam Users (Reference Group), in the Unweighted and Weighted Populations, Primary Analysis **eFigure 4.** Weighted Risk Difference Estimates of Any Congenital Malformation in Infants Born to Individuals Treated for Urinary Tract Infection (UTI) in the First Trimester: Comparison of Different UTI-related Antibiotics Versus β-lactams (Reference) **eFigure 5.** Weighted Risk Difference Estimates of Any Cardiac Malformation in Infants Born to Individuals Treated for Urinary Tract Infection (UTI) in the First Trimester: Comparison of Different UTI-related Antibiotics Versus β-lactams (Reference) **eFigure 6.** Weighted Risk Ratio Estimates of Other Organ-Specific Malformations in Infants Born to Individuals Treated for Urinary Tract Infection (UTI) in the First Trimester: Comparison of Different UTI-related Antibiotics Versus β-lactams (Reference) **eFigure 7.** Weighted Risk Difference Estimates of Other Organ-Specific Malformations in Infants Born to Individuals Treated for Urinary Tract Infection (UTI) in the First Trimester: Comparison of Different UTI-related Antibiotics Versus β-lactams (Reference) **eFigure 8.** Weighted Risk Difference Estimates of Specific Malformation Groups in Infants Born to Individuals Treated for Urinary Tract Infection (UTI) in the First Trimester: Comparison of Different UTI-related Antibiotics Versus β-lactams (Reference) **eFigure 9.** Weighted Risk Ratio Estimates of Severe Cardiac Defect, Other Cardiac Defect, and Cleft Lip and/or Cleft Palate in Infants Born to Individuals Treated for Urinary Tract Infection (UTI) in the First Trimester: Comparison of Different UTI-related Antibiotics Versus β-lactams (Reference) **eFigure 10.** Weighted Risk Difference Estimates of Severe Cardiac Defect, Other Cardiac Defect, and Cleft Lip and/or Cleft Palate in Infants Born to Individuals Treated for Urinary Tract Infection (UTI) in the First Trimester: Comparison of Different UTI-related Antibiotics Versus β-lactams (Reference) **eFigure 11.** Absolute Risk of Congenital Malformations Among Infants Born to Individuals Treated with a UTI-related Antibiotic for Any Indication in the First Trimester by Index Antibiotic Agent and Indication **eFigure 12.** Weighted Risk Difference Estimates of Any Congenital Malformation in Infants Born to Individuals Treated with a UTI-related Antibiotic for Any Indication in the First Trimester: Comparison of Different UTI-related Antibiotics Versus β-lactams (Reference) **eFigure 13.** Weighted Risk Ratio Estimates of Any Congenital Malformation in Infants Born to Individuals Treated with a UTI-related Antibiotic for Any Indication in the First Trimester: Comparison of Different UTI-related Antibiotics Versus β-lactams (Reference) **eFigure 14.** Weighted Risk Difference Estimates of Any Cardiac Malformation in Infants Born to Individuals Treated with a UTI-related Antibiotic for Any Indication in the First Trimester: Comparison of Different UTI-related Antibiotics Versus β-lactams (Reference) **eFigure 15.** Weighted Risk Ratio Estimates of Any Cardiac Malformation in Infants Born to Individuals Treated with a UTI-related Antibiotic for Any Indication in the First Trimester: Comparison of Different UTI-related Antibiotics Versus β-lactams (Reference) **eFigure 16.** Selection Bias-Corrected Corrected Weighted Risk Ratio Estimates of (A) Any Malformation and (B) Any Cardiac Malformation in Infants Born to Individuals Treated for Urinary Tract Infection (UTI) in the First Trimester: TMP-SMX Versus β-lactam (Reference) **eFigure 17.** Selection Bias-Corrected Weighted Risk Ratio Estimates of (A) Any Malformation and (B) Any Cardiac Malformation in Infants Born to Individuals Treated for Urinary Tract Infection (UTI) in the First Trimester: Fluoroquinolone Versus β-lactam (Reference)
eAppendix.

Supplement 2. **eTable 23.** Code List for eTable 1 **eTable 24.** Code List for eTable 2 **eTable 25.** Code List for eTable 3 **eTable 26.** Code List for eTable 5 **eTable 27.** ICD-9-CM/ICD-10-CM Diagnosis and HCPCS Codes for eTable 6 **eTable 28.** Medication Names Derived From NDC Codes for eTable 6 **eTable 29.** Code List for eTable 7 **eTable 30.** ICD-9-CM/ICD-10-CM Diagnosis, CPT Codes, HCPCS Codes, and UB-04 Revenue Codes for eTable 8 **eTable 31.** Medication Names Derived From NDC Codes for eTable 8

Supplement 3.
Data Sharing Statement
