# Supplementary material for: First-Trimester Antibiotic Use for Urinary Tract Infection and Risk of Congenital Malformations
Source: JAMA Netw Open. 2025 Jul 9;8(7):e2519544. doi: 10.1001/jamanetworkopen.2025.19544 (PMC12242685; doi:10.1001/jamanetworkopen.2025.19544)
Supplement: Supplement 1. — eMethods. eReferences. eTable 1. Codes to Identify Pregnancy Outcomes eTable 2. Codes to Verify Pregnancy Outcome eTable 3. Codes to Determine Gestational Age eTable 4. Oral Antibiotic Categories for Agents Used to Treat Urinary Tract Infection eTable 5. Codes to Identify Possible Antibiotic Indication(s) ± 7 Days from the Index Antibiotic Date eTable 6. Codes to Identify Individuals for Exclusion eTable 7. Outcome Definitions for Congenital Malformations Based on Validated Algorithms by Kharbanda et al eTable 8. Codes to Identify Covariates eTable 9. Distribution of Urinary Tract Infection-related Oral Antibiotic Combinations that Resulted in Exclusion from the Study Cohort eTable 10. Distribution of UTI Indication Criterion Qualification among Individuals Treated for Urinary Tract Infection in the First Trimester of Pregnancy eTable 11. Distribution of Index Oral Antibiotic Agents among Individuals Treated for Urinary Tract Infection in the First Trimester of Pregnancy eTable 12. Characteristics of Pregnancies Among Individuals Treated for Urinary Tract Infection in the First Trimester by Index Antibiotic Agent, After Propensity Score Weighting eTable 13. Absolute Risk of Congenital Malformations Among Infants Born to Individuals Treated for Urinary tract infection in the First Trimester With an Antibiotic Agent by Organ-Specific Malformation Group and Outcome Definition eTable 14. Absolute Risk of Congenital Malformations Among Infants Born to Individuals Treated for Urinary Tract Infection in the First Trimester by Index Antibiotic Agent eTable 15. Crude and Weighted Risk Difference and Risk Ratio Estimates of Any Congenital Malformation in Infants Born to Individuals Treated for Urinary Tract Infection (UTI) in the First Trimester: Comparison of Different UTI-related Antibiotics Versus β-lactams (Reference) eTable 16. Crude and Weighted Risk Difference and Risk Ratio Estimates of Any Cardiac Malformation in Infants Born to Individuals Treated for Urinary Tract [file jamanetwopen-e2519544-s001.pdf]

Supplemental Online Content

Osmundson SS, Nickel KB, Shortreed SM, et al. First-trimester antibiotic use for urinary tract infection and risk of congenital malformations. *JAMA Netw Open.* 2025;8(7):e2519544. doi:10.1001/jamanetworkopen.2025.19544

eMethods.

eReferences.

eTable 1. Codes to Identify Pregnancy Outcomes

eTable 2. Codes to Verify Pregnancy Outcome

eTable 3. Codes to Determine Gestational Age

eTable 4. Oral Antibiotic Categories for Agents Used to Treat Urinary Tract Infection

eTable 5. Codes to Identify Possible Antibiotic Indication(s) ± 7 Days from the Index Antibiotic Date

eTable 6. Codes to Identify Individuals for Exclusion

eTable 7. Outcome Definitions for Congenital Malformations Based on Validated Algorithms by Kharbanda et al

eTable 8. Codes to Identify Covariates

eTable 9. Distribution of Urinary Tract Infection-related Oral Antibiotic Combinations that Resulted in Exclusion from the Study Cohort

eTable 10. Distribution of UTI Indication Criterion Qualification among Individuals Treated for Urinary Tract Infection in the First Trimester of Pregnancy

eTable 11. Distribution of Index Oral Antibiotic Agents among Individuals Treated for Urinary Tract Infection in the First Trimester of Pregnancy

eTable 12. Characteristics of Pregnancies Among Individuals Treated for Urinary Tract Infection in the First Trimester by Index Antibiotic Agent, After Propensity Score Weighting

eTable 13. Absolute Risk of Congenital Malformations Among Infants Born to Individuals Treated for Urinary tract infection in the First Trimester With an Antibiotic Agent by Organ-Specific Malformation Group and Outcome Definition

eTable 14. Absolute Risk of Congenital Malformations Among Infants Born to Individuals Treated for Urinary Tract Infection in the First Trimester by Index Antibiotic Agent

© 2025 Osmundson SS et al. *JAMA Network Open*.

eTable 15. Crude and Weighted Risk Difference and Risk Ratio Estimates of Any Congenital Malformation in Infants Born to Individuals Treated for Urinary Tract Infection (UTI) in the First Trimester: Comparison of Different UTI-related Antibiotics Versus β-lactams (Reference)

eTable 16. Crude and Weighted Risk Difference and Risk Ratio Estimates of Any Cardiac Malformation in Infants Born to Individuals Treated for Urinary Tract Infection (UTI) in the First Trimester: Comparison of Different UTI-related Antibiotics Versus β-lactams (Reference)

eTable 17. Crude and Weighted Risk Difference and Risk Ratio Estimates of Other Organ-Specific Malformations in Infants Born to Individuals Treated for Urinary Tract Infection (UTI) in the First Trimester: Comparison of Different UTI-related Antibiotics Versus β-lactams (Reference)

eTable 18. Crude and Weighted Risk Difference and Risk Ratio Estimates of Specific Malformation Groups in Infants Born to Individuals Treated for Urinary Tract Infection (UTI) in the First Trimester: Comparison of Different UTI-related Antibiotics Versus β-lactams (Reference)

eTable 19. E-values and Associated Weighted Risk Ratio Estimates of Any Congenital Malformation, Any Cardiac Malformation, Other Organ-Specific Congenital Malformations, and Specific Congenital Malformations in Infants Born to Individuals Treated for Urinary Tract Infection (UTI) in the First Trimester: Comparison of Different UTI-related Antibiotics Versus β-lactams (Reference)

eTable 20. Characteristics of Pregnancies Among Individuals Treated with a UTI-related Antibiotic for Any Indication in the First Trimester by Index Antibiotic Agent (N=275,413)

eTable 21. Characteristics of Pregnancies Among Individuals Treated with a UTI-related Antibiotic for Any Indication in the First Trimester by Index Antibiotic Agent, After Propensity Score Weighting

eTable 22. Potential Values of the Probability of Livebirth for β-lactam and Trimethoprim-sulfamethoxazole Under the Assumption of a Higher Probability of Livebirth for β-lactam and Corrected Risk Ratios for Any Malformation

eFigure 1. Derivation of Cohort of Pregnant Individuals Treated for Urinary Tract Infection (UTI) in the First Trimester with an Antibiotic Agent in the MarketScan Commercial Database

eFigure 2. Utilization of Antibiotic Agents for the Treatment of Urinary Tract Infection During the First Trimester of Pregnancy by Calendar Year

eFigure 3. Standardized Mean Differences of Patient Characteristics Among Pregnant Individuals Treated for Urinary Tract Infection in the First Trimester Between Each Antibiotic Exposure Group versus β-lactam Users (Reference Group), in the Unweighted and Weighted Populations, Primary Analysis

eFigure 4. Weighted Risk Difference Estimates of Any Congenital Malformation in Infants Born to Individuals Treated for Urinary Tract Infection (UTI) in the First Trimester: Comparison of Different UTI-related Antibiotics Versus β-lactams

© 2025 Osmundson SS et al. *JAMA Network Open*.

(Reference)

eFigure 5. Weighted Risk Difference Estimates of Any Cardiac Malformation in Infants Born to Individuals Treated for Urinary Tract Infection (UTI) in the First Trimester: Comparison of Different UTI-related Antibiotics Versus β-lactams (Reference)

eFigure 6. Weighted Risk Ratio Estimates of Other Organ-Specific Malformations in Infants Born to Individuals Treated for Urinary Tract Infection (UTI) in the First Trimester: Comparison of Different UTI-related Antibiotics Versus β-lactams (Reference)

eFigure 7. Weighted Risk Difference Estimates of Other Organ-Specific Malformations in Infants Born to Individuals Treated for Urinary Tract Infection (UTI) in the First Trimester: Comparison of Different UTI-related Antibiotics Versus β-lactams (Reference)

eFigure 8. Weighted Risk Difference Estimates of Specific Malformation Groups in Infants Born to Individuals Treated for Urinary Tract Infection (UTI) in the First Trimester: Comparison of Different UTI-related Antibiotics Versus β-lactams (Reference)

eFigure 9. Weighted Risk Ratio Estimates of Severe Cardiac Defect, Other Cardiac Defect, and Cleft Lip and/or Cleft Palate in Infants Born to Individuals Treated for Urinary Tract Infection (UTI) in the First Trimester: Comparison of Different UTI-related Antibiotics Versus β-lactams (Reference)

eFigure 10. Weighted Risk Difference Estimates of Severe Cardiac Defect, Other Cardiac Defect, and Cleft Lip and/or Cleft Palate in Infants Born to Individuals Treated for Urinary Tract Infection (UTI) in the First Trimester: Comparison of Different UTI-related Antibiotics Versus β-lactams (Reference)

eFigure 11. Absolute Risk of Congenital Malformations Among Infants Born to Individuals Treated with a UTI-related Antibiotic for Any Indication in the First Trimester by Index Antibiotic Agent and Indication

eFigure 12. Weighted Risk Difference Estimates of Any Congenital Malformation in Infants Born to Individuals Treated with a UTI-related Antibiotic for Any Indication in the First Trimester: Comparison of Different UTI-related Antibiotics Versus β-lactams (Reference)

eFigure 13. Weighted Risk Ratio Estimates of Any Congenital Malformation in Infants Born to Individuals Treated with a UTI-related Antibiotic for Any Indication in the First Trimester: Comparison of Different UTI-related Antibiotics Versus β-lactams (Reference)

eFigure 14. Weighted Risk Difference Estimates of Any Cardiac Malformation in Infants Born to Individuals Treated with a UTI-related Antibiotic for Any Indication in the First Trimester: Comparison of Different UTI-related Antibiotics Versus β-lactams (Reference)

eFigure 15. Weighted Risk Ratio Estimates of Any Cardiac Malformation in Infants Born to Individuals Treated with a UTI-related Antibiotic for Any Indication in the First Trimester: Comparison of Different UTI-related Antibiotics Versus β-lactams

© 2025 Osmundson SS et al. *JAMA Network Open*.

(Reference)

eFigure 16. Selection Bias-Corrected Corrected Weighted Risk Ratio Estimates of (A) Any Malformation and (B) Any Cardiac Malformation in Infants Born to Individuals Treated for Urinary Tract Infection (UTI) in the First Trimester: TMP-SMX Versus β-lactam (Reference)

eFigure 17. Selection Bias-Corrected Weighted Risk Ratio Estimates of (A) Any Malformation and (B) Any Cardiac Malformation in Infants Born to Individuals Treated for Urinary Tract Infection (UTI) in the First Trimester: Fluoroquinolone Versus β-lactam (Reference)

This supplemental material has been provided by the authors to give readers additional information about their work.

© 2025 Osmundson SS et al. *JAMA Network Open*.

eMethods.

Identification and adjudication of pregnancy outcomes

Identification of pregnancy outcomes

We extracted pregnancy outcome codes for the outcomes of livebirth, stillbirth, multiple gestation with live and stillbirth, unknown delivery, ectopic, spontaneous abortion, induced abortion, and unspecified abortion from inpatient and outpatient claims (eTable 1).[1,2] From the first pregnancy outcome date per person, we grouped pregnancy outcome claims within ≤30 days as part of a single pregnancy outcome *episode* for further adjudication. A pregnancy outcome *episode* containing one or more pregnancy outcome codes identified during an inpatient admission was considered an inpatient pregnancy outcome episode. A pregnancy outcome code was classified as inpatient if located in: (a) the inpatient file; (b) the facility header file and linked to an inpatient claim; or (c) the outpatient file or facility header file and had a service date between the admission and discharge dates of a hospitalization from the inpatient file. A pregnancy outcome code was classified as an outpatient claim if it did not meet any of those criteria. For inpatient pregnancy outcomes, we used the admission date as the pregnancy outcome date; the date of service served as the pregnancy outcome date for outpatient pregnancy outcomes.

Assignment of pregnancy outcome type

Next, we established the final pregnancy outcome type based on the pregnancy outcome codes: livebirth, stillbirth, mixed birth, spontaneous abortion, induced abortion, unspecified abortion, or ectopic. This was determined using a published algorithm[3] and adapted to include ICD-10-CM diagnosis and ICD-10-PCS procedure codes. The algorithm used a hierarchy to assign the most likely pregnancy outcome type based on other corroborating codes on claims within 30 days.

Confirmatory evidence for pregnancy outcome and assignment of pregnancy outcome date:

After identifying and classifying pregnancy outcomes, we reviewed each potential pregnancy outcome episode for confirmatory evidence that indicated a pregnancy took place. Our goals were to: 1) exclude pregnancy outcomes lacking confirmatory evidence, since if the pregnancy outcome did not occur then the individual was not pregnant; and 2) determine the date of the pregnancy outcome, which is used in subsequent algorithms to determine timing of other pregnancy dates such as the date of the last menstrual period (LMP).

Specifically, we used 7 pieces of confirmatory evidence that indicated a pregnancy: (1) both a provider and facility pregnancy outcome code (eTable 1); (2) both a diagnosis and procedure pregnancy outcome code (eTable 1); (3) revenue code for labor and delivery (eTable 2); (4) gestational age diagnosis code (eTable 3); (5) pregnancy-related care code (eTable 2); (6) infant birth claim (eTable 2); (7) infant insurance enrollment and linkage to a birthing parent (see “Linkage of birthing parent to infant”). Evidence of pregnancy-related care was assessed in the 30 days before each pregnancy outcome date within the pregnancy episode. All other pieces of confirmatory evidence (besides enrollment information) were assessed during the inpatient admission (for inpatient episodes) and on the service date of each potential pregnancy outcome date (for outpatient pregnancy outcome episodes).

While a pregnancy outcome episode could contain more than one potential pregnancy outcome date, results were aggregated at the pregnancy episode-level (e.g., if at least one date had confirmatory evidence it was considered a confirmed pregnancy outcome and a confirmed pregnancy episode). In the event of more than one pregnancy outcome date being corroborated by confirmatory evidence, we selected a pregnancy outcome date based upon a hierarchy of the 7 pieces of confirmatory evidence, where the first confirmatory piece of evidence (“both a provider and facility pregnancy outcome code”) had highest priority. For example, if a potential pregnancy outcome date met the second piece of confirmatory evidence (“both a diagnosis and procedure pregnancy outcome code”), that date would be established as the pregnancy outcome date over another potential pregnancy outcome date in which the date matched the third piece of confirmatory evidence (“revenue code for labor and delivery”). In the event of a tie between multiple dates at the same priority level, the earliest date was selected as the pregnancy outcome date. If a pregnancy outcome did not have at least 1 of the 7 pieces of confirmatory evidence, the pregnancy outcome was removed from the cohort.

© 2025 Osmundson SS et al. *JAMA Network Open*.

Final adjudication of pregnancy outcomes
After excluding pregnancy outcome episodes that did not have confirmatory evidence, we examined anomalous pregnancy outcome timing to determine the final pregnancy outcomes per individual based on MacDonald 2019.[3] Two livebirth/stillbirth/mixed birth pregnancy outcome dates less than 211 days apart and spontaneous/induced/unspecified abortions less than 61 days apart were considered biologically implausible. A hierarchical list of prioritized pregnancy outcomes was used to determine which pregnancy outcome should be used (e.g., for two livebirths, an infant-linked livebirth was prioritized over an unlinked livebirth). This process was iterated until no abnormally-timed pregnancy outcomes remained.

Gestational age and last menstrual period date assignment
We determined the gestational age at the end of pregnancy to calculate the date of LMP. LMP (day 0 of pregnancy) was used to establish the timing of the index antibiotic during pregnancy and define a lookback window for variables during and before pregnancy. Gestational age at the end of pregnancy was determined using two methods. In the first method, ICD-9-CM/ICD-10-CM diagnosis codes indicating gestational age at time of the pregnancy outcome were captured (eTable 3)[4]; for inpatient pregnancy outcomes, inpatient codes ±7 days of pregnancy outcome date were used; for outpatient pregnancy outcomes, outpatient codes ±7 days of pregnancy outcome date were used. If more than one code indicating gestational age was available, we used a hierarchy to assign final gestational age.[4] If a gestational age diagnosis code was not available, we used a second method that used dates of pregnancy-related screening tests that are performed at specific gestational ages to estimate gestational age (ICD-9-CM/ICD-10-CM diagnosis or CPT codes; eTable 3).[5] In the event of multiple screening test types and/or screening test dates, we used a published hierarchy to choose a final gestational age at pregnancy outcome.[5] Finally, if the gestational age at pregnancy outcome was still not assigned, we assigned a default of 273 days gestational age at delivery for liveborn, stillborn, or mixed birth outcomes[4] or 84 days for ectopic, spontaneous abortion, induced abortion, or unspecified abortion. The gestational age at the end of pregnancy (in days) was subtracted from the end of pregnancy date to determine the LMP date.

Linkage of birthing parent to infant
Infants were linked to the birthing parent using a family identification number in the insurance enrollment files and linked to a particular liveborn delivery based on a reported or imputed date of birth ± 15 days from the delivery date. The date of an infant birth claim (per eTable 2) was used as the reported date of birth. We also used the enrollment files to impute a date of birth using two methods. The first month of enrollment for an infant with an age of 0 years was considered a month of birth if it corresponded with a birthing parent delivery claim in the same month or the month prior; the date of delivery was assigned as the birth date. Second, infant month and year of birth were estimated via the month the child age changed from 0 to 1 year (or 1-2, 3-4, or 4-5 years) in the monthly insurance enrollment data, and the 16th of the month was assigned as the birth date. Finally, for the purposes of creating a linked birthing parent-infant cohort (but not done for the purposes of delivery verification), we required that infant enrollment started within 60 days of birth.

Diagnostic and rule-out claim exclusion
For diagnosis codes other than those found on inpatient facility claims, we ignored diagnosis codes on claim lines with CPT/HCPCS codes that would suggest a diagnostic/rule-out diagnosis or a diagnosis unlikely to have been made by a provider. Specifically, we ignored diagnosis codes on claim lines with the following CPT codes: 36400–36425, 36591, 36592, 36600, 70000–77086, 80000–87999, 88400, 88720–89399, 90460–90779, 99000–99002, 99080–99082 which encompass blood collection, diagnostic radiology, radiologic guidance, mammography, bone/joint studies, laboratory besides pathology, vaccines, and specimen handling/transfer and the following HCPCS codes reflecting transportation services, administrative services, temporary codes, laboratory, and diagnostic radiology: A0021–A0999, A9150–A9999, G0008–G9987, P2028–P9615, Q0035–Q9992, R0070–R0076.

© 2025 Osmundson SS et al. *JAMA Network Open*.

eReferences

1. Ailes EC, Zhu W, Clark EA, et al. Identification of pregnancies and their outcomes in healthcare claims data, 2008-2019: An algorithm. *PloS one*. 2023;18(4):e0284893. doi:10.1371/journal.pone.0284893
2. Palmsten K, Huybrechts KF, Mogun H, et al. Harnessing the Medicaid Analytic eXtract (MAX) to Evaluate Medications in Pregnancy: Design Considerations. *PloS one*. 2013;8(6):e67405. doi:10.1371/journal.pone.0067405
3. MacDonald SC, Cohen JM, Panchaud A, McElrath TF, Huybrechts KF, Hernández-Díaz S. Identifying pregnancies in insurance claims data: Methods and application to retinoid teratogenic surveillance. *Pharmacoepidemiology and drug safety*. Sep 2019;28(9):1211-1221. doi:10.1002/pds.4794
4. Sentinel Initiative. Characterizing Pregnant Women With and Without Evidence of Heart Failure and Non-Pregnant Women With Heart Failure: A Propensity Score Matched Analysis. Accessed April 4, 2024, https://dev.sentinelsystem.org/projects/AP/repos/sentinel-analytic-packages/browse?at=refs%2Fheads%2Fcder_mpl2p_wp029
5. Zhu Y, Thai TN, Hernandez-Diaz S, et al. Development and Validation of Algorithms to Estimate Live Birth Gestational Age in Medicaid Analytic eXtract Data. *Epidemiology*. Jan 1 2023;34(1):69-79. doi:10.1097/EDE.0000000000001559
6. Suarez EA, Landi SN, Conover MM, Jonsson Funk M. Bias from restricting to live births when estimating effects of prescription drug use on pregnancy complications: A simulation. *Pharmacoepidemiology and drug safety*. Mar 2018;27(3):307-314. doi:10.1002/pds.4387
7. Lash TF, M; MacLehose, R;. *Applying Quantitative Bias Analysis to Epidemiologic Data*. Second ed. Springer International Publishing; 2021.
8. Huybrechts KF, Hernández-Díaz S, Straub L, et al. Association of Maternal First-Trimester Ondansetron Use With Cardiac Malformations and Oral Clefts in Offspring. *Jama*. Dec 18 2018;320(23):2429-2437. doi:10.1001/jama.2018.18307

eTable 1. Codes to Identify Pregnancy Outcomes[a]

Description	Code type(s)
Liveborn delivery	ICD-9-CM diagnosis<br>ICD-10-CM diagnosis
Stillbirth delivery	ICD-9-CM diagnosis<br>ICD-10-CM diagnosis
Mixed birth delivery	ICD-9-CM diagnosis<br>ICD-10-CM diagnosis
Unspecified delivery	CPT<br>ICD-9-CM diagnosis<br>ICD-10-CM diagnosis<br>ICD-9-CM procedure<br>ICD-10-PCS procedure
Spontaneous abortion	CPT<br>ICD-9-CM diagnosis<br>ICD-10-CM diagnosis
Induced abortion	CPT<br>HCPCS<br>ICD-9-CM diagnosis<br>ICD-10-CM diagnosis<br>ICD-9-CM procedure<br>ICD-10-PCS procedure
Unspecified abortion	ICD-9-CM diagnosis<br>ICD-10-CM diagnosis
Ectopic	CPT<br>ICD-9-CM diagnosis<br>ICD-10-CM diagnosis<br>ICD-9-CM procedure<br>ICD-10-PCS procedure

Abbreviations: CPT, Current Procedural Terminology; HCPCS, Healthcare Common Procedure Coding System; ICD-9-CM, International Classification of Diseases, Ninth Revision, Clinical Modification; ICD-10-CM, International Classification of Diseases, Tenth Revision, Clinical Modification; ICD-10-PCS, International Classification of Diseases, Tenth Revision, Procedure Coding System.

[a] For code lists, see supplemental spreadsheet tab named eTable 23 in Supplement 2.

© 2025 Osmundson SS et al. *JAMA Network Open*.

eTable 2. Codes to Verify Pregnancy Outcome[a]

Description	Timing	Code type(s)
Labor and delivery	During delivery admission or ± 7 days from outpatient pregnancy outcome date	UB-04 revenue code
Pregnancy-related care	-30 to 0 from pregnancy outcome date	CPT-4<br>ICD-9-CM diagnosis<br>ICD-10-CM diagnosis<br>ICD-9-CM procedure<br>ICD-10-PCS procedure
Newborn claim	During delivery admission or ± 7 days from outpatient pregnancy outcome date	ICD-9-CM diagnosis<br>ICD-10-CM diagnosis

Abbreviations: CPT, Current Procedural Terminology; ICD-9-CM, International Classification of Diseases, Ninth Revision, Clinical Modification; ICD-10-CM, International Classification of Diseases, Tenth Revision, Clinical Modification; ICD-10-PCS, International Classification of Diseases, Tenth Revision, Procedure Coding System; UB-04, uniform billing.

[a] For code lists, see supplemental spreadsheet tab named eTable 24 in Supplement 2.

© 2025 Osmundson SS et al. *JAMA Network Open*.

eTable 3. Codes to Determine Gestational Age[a]

Description	Timing	Code type(s)
Gestational age codes	± 7 days from pregnancy outcome date, using only inpatient codes if inpatient pregnancy outcome	ICD-9-CM diagnosis<br>ICD-10-CM diagnosis
Screening tests	During pregnancy[b]	CPT<br>ICD-9-CM diagnosis<br>ICD-10-CM diagnosis

Abbreviations: CPT, Current Procedural Terminology; ICD-9-CM, International Classification of Diseases, Ninth Revision, Clinical Modification; ICD-10-CM, International Classification of Diseases, Tenth Revision, Clinical Modification.

[a] For code lists, see supplemental spreadsheet tab named eTable 25 in Supplement 2.
[b] During pregnancy defined as last menstrual period date through pregnancy outcome date.

© 2025 Osmundson SS et al. *JAMA Network Open*.

eTable 4. Oral Antibiotic Categories for Agents Used to Treat Urinary Tract Infection

Antibiotic agent/class	Generic names
Nitrofurantoin	nitrofurantoin; nitrofurantoin monohydrate/nitrofurantoin, macro; nitrofurantoin, macrocrystals
Trimethoprim-sulfamethoxazole	sulfamethoxazole/trimethoprim<br><br>trimethoprim[a]; trimethoprim hydrochloride[a]
Fluoroquinolone	ciprofloxacin; ciprofloxacin hydrochloride; ciprofloxacin/ciprofloxacin hydrochloride; levofloxacin; ofloxacin<br><br>delafloxacin[a]; enoxacin[a]; gatifloxacin[a]; gemifloxacin mesylate[a]; grepafloxacin hydrochloride[a]; lomefloxacin hydrochloride[a]; moxifloxacin hydrochloride[a]; norfloxacin[a]; sparfloxacin[a]; trovafloxacin mesylate[a]
β-lactam (cephalosporins and penicillins)	amoxicillin[b]; amoxicillin/clavulanate potassium; ampicillin[b]; cefaclor; cefdinir; cefpodoxime proxetil; cephalexin; cephalexin hydrochloride<br><br>cefadroxil[a]; cefditoren pivoxil[a]; cefixime[a]; ceftibuten[a]; cefuroxime axetil[a]; cephradine[a]; loracarbef[a]
Fosfomycin[c]	fosfomycin tromethamine

[a] These agents were not included in the study population as they are not specifically recommended in urinary tract infection (UTI) guidelines, but belong to the same class as recommended agents. They were used as an exclusion when requiring exposure to a single UTI antibiotic class during the first trimester.
[b] Amoxicillin-alone and ampicillin-alone are not recommended for the treatment of urinary tract infection, however they are used in practice.
[c] Fosfomycin was not included in the study population due to very rare use. However, fosfomycin use was used as an exclusion when requiring exposure to a single UTI antibiotic class during the first trimester.

© 2025 Osmundson SS et al. *JAMA Network Open*.

eTable 5. Codes to Identify Possible Antibiotic Indication(s) ± 7 Days from the Index Antibiotic Date[a,b]

**Description**	**Code type(s)**
**UTI**	
UTI diagnosis	ICD-9-CM diagnosis<br>ICD-10-CM diagnosis
UTI symptom	ICD-9-CM diagnosis<br>ICD-10-CM diagnosis
Evidence of positive urine culture[c]	CPT
**Other infection**	
Abdominal infection	ICD-9-CM diagnosis<br>ICD-10-CM diagnosis
Dental infection	ICD-9-CM diagnosis<br>ICD-10-CM diagnosis
Lower respiratory infection[d]	ICD-9-CM diagnosis<br>ICD-10-CM diagnosis
Upper respiratory infection[e]	ICD-9-CM diagnosis<br>ICD-10-CM diagnosis
Pyelonephritis	ICD-9-CM diagnosis<br>ICD-10-CM diagnosis
Skin and soft tissue infection	ICD-9-CM diagnosis<br>ICD-10-CM diagnosis

Abbreviations: CPT, Current Procedural Terminology; ICD-9-CM, International Classification of Diseases, Ninth Revision, Clinical Modification; ICD-10-CM, International Classification of Diseases, Tenth Revision, Clinical Modification; UTI, urinary tract infection.

[a] For code lists, see supplemental spreadsheet tab named eTable 26 in Supplement 2. An index antibiotic could be associated with >1 indication.
[b] For UTI, outpatient UTI diagnosis, symptom, and evidence of positive urine culture were captured separately from inpatient UTI diagnosis or symptom in order to identify the outpatient UTI cohort for the primary analyses. All other conditions include claims from the inpatient and outpatient setting combined.
[c] To meet the “evidence of positive urine culture” criterion, codes for both a urine culture test and a susceptibility test (only performed on positive cultures) were required.
[d] Lower respiratory infection included acute bronchitis and pneumonia.
[e] Upper respiratory infection included acute otitis media, pharyngitis, sinusitis, viral upper respiratory infection.

© 2025 Osmundson SS et al. *JAMA Network Open*.

eTable 6. Codes to Identify Individuals for Exclusion[a,b]

Description	Timing	Code type(s)
**Pregnancy-related**		
Multiple gestation	During pregnancy[c]	ICD-9-CM diagnosis<br>ICD-10-CM diagnosis
Chromosomal abnormality	During pregnancy[c] including newborn claims during delivery admission	ICD-9-CM diagnosis<br>ICD-10-CM diagnosis
**Immune suppression-related**		
HIV	Baseline[d]	ICD-9-CM diagnosis<br>ICD-10-CM diagnosis
Cancer (solid tumor, metastases, lymphoma, leukemia)	Baseline[d]	ICD-9-CM diagnosis<br>ICD-10-CM diagnosis
Hereditary anemia	Baseline[d]	ICD-9-CM diagnosis<br>ICD-10-CM diagnosis
Aplastic anemia	Baseline[d]	ICD-9-CM diagnosis<br>ICD-10-CM diagnosis
Hereditary immunodeficiency	Baseline[d]	ICD-9-CM diagnosis<br>ICD-10-CM diagnosis
Coagulation/hemorrhagic	Baseline[d]	ICD-9-CM diagnosis<br>ICD-10-CM diagnosis
Leukopenia	Baseline[d]	ICD-9-CM diagnosis<br>ICD-10-CM diagnosis
Hemophagocytic syndrome	Baseline[d]	ICD-9-CM diagnosis<br>ICD-10-CM diagnosis
Sarcoidosis	Baseline[d]	ICD-9-CM diagnosis<br>ICD-10-CM diagnosis
Polyarteritis nodosa and related condition	Baseline[d]	ICD-9-CM diagnosis<br>ICD-10-CM diagnosis
Diffuse diseases of connective tissue	Baseline[d]	ICD-9-CM diagnosis<br>ICD-10-CM diagnosis
Transplantation	Baseline[d]	ICD-9-CM diagnosis<br>ICD-10-CM diagnosis
Rheumatologic condition	Baseline[d]	ICD-9-CM diagnosis<br>ICD-10-CM diagnosis
Inflammatory bowel disease	Baseline[d]	ICD-9-CM diagnosis<br>ICD-10-CM diagnosis
Asplenia	Baseline[d]	ICD-9-CM diagnosis<br>ICD-10-CM diagnosis
Autoimmune medications, short-acting	-30 to 0 before index antibiotic date	HCPCS<br>NDC
Autoimmune medications, other	Baseline[d]	HCPCS<br>NDC
Spinal cord injury	Baseline[d]	ICD-9-CM diagnosis<br>ICD-10-CM diagnosis
**Known teratogenic medication**		
Antineoplastic agent	Days' supply overlapping the first trimester or service date during the first trimester	HCPCS<br>NDC
Isotretinoin	Days' supply overlapping the first trimester	NDC
Lithium	Days' supply overlapping the first trimester	NDC

© 2025 Osmundson SS et al. *JAMA Network Open*.

Description	Timing	Code type(s)
Misoprostol	Days' supply overlapping the first trimester or service date during the first trimester	HCPCS<br>NDC
Thalidomide	Days' supply overlapping the first trimester	NDC
Warfarin	Days' supply overlapping the first trimester	NDC
**Antibiotic exposure**		
Index antibiotic, systemic non-oral	First trimester	HCPCS<br>NDC
Infection-related hospitalization[e]	Overlapping the first trimester	Hospitalization per MarketScan inpatient file<br>ICD-9-CM diagnosis<br>ICD-10-CM diagnosis

Abbreviations: HCPCS, Healthcare Common Procedure Coding System; HIV, human immunodeficiency virus; ICD-9-CM, International Classification of Diseases, Ninth Revision, Clinical Modification; ICD-10-CM, International Classification of Diseases, Tenth Revision, Clinical Modification; NDC, national drug code.

[a] For code lists, see supplemental spreadsheet tab named eTable 27 in Supplement 2 for ICD-9-CM/ICD-10-CM diagnosis and HCPCS codes; see tab named eTable 28 in Supplement 2 for medication names derived from NDC codes.
[b] All medications restricted to systemic routes of administration (i.e., buccal mucosa, implantation, injection, intradermal, intramuscular, intravenous, oral, oromucosal, subcutaneous, sublingual, transdermal).
[c] During pregnancy defined as last menstrual period date through pregnancy outcome date.
[d] Baseline period for diagnosis codes was defined as last menstrual period date -180 through index antibiotic date. Baseline period for medications was defined as last menstrual period date -90 through index antibiotic date.
[e] Infection-related hospitalization included abdominal infection, bone infection, clinical sepsis, dental infection, lower and upper respiratory infection, miscellaneous bacterial infection, organ infection, otitis, pelvic infection, septicemia, skin and soft tissue infection, surgical site infection, tonsilitis, and urinary tract infection (including pyelonephritis).

© 2025 Osmundson SS et al. *JAMA Network Open*.

eTable 7. Outcome Definitions for Congenital Malformations Based on Validated Algorithms by Kharbanda et al[a]

Organ system	Specific malformation group	ICD-9-CM diagnosis codes	ICD-10-CM diagnosis codes	(Primary definition) Modified algorithm
Central nervous system[b]	Neural tube defects: encephalocele, cranial meningocele, encephalomyelocele, spina bifida	741.0x, 741.9x, 742.0	Q01.x, Q05.x, Q07.01, Q07.03	1 inpatient diagnosis or 2 outpatient diagnoses
	Holoprosencephaly	742.2	Q04.2	2 outpatient diagnoses
Eye	Anophthalmia, microphthalmia, cataracts and other lens defects	743.00, 743.10–743.12, 743.2x, 743.30–743.36,	Q11.1, Q11.2, Q12.0, Q12.3, Q12.4, Q12.8	2 outpatient diagnosis
Ear	Anotia, microtia	744.01, 744.23	Q16.0, Q16.1, Q17.2	2 outpatient diagnoses
Cardiac	Severe cardiac defects: single ventricle, tricuspid atresia, ebstein anomaly, hypoplastic left heart, hypoplastic right heart, common truncus, transposition, atrioventricular septal defects, tetralogy of fallot, aortic valve atresia or stenosis, coarctation, total anomalous pulmonary venous return[c]	745.0, 745.1x, 745.2–745.3, 745.6x, 745.7, 746.2, 746.3, 746.7, 747.1x, 747.22, 747.41	Q20.0, Q20.1, Q20.2, Q20.3–Q20.5, Q21.2–Q21.4, Q22.5–Q22.6, Q23.0, Q23.4, Q25.1, Q25.2x, Q25.3, Q25.41, Q25.42, Q25.5, Q26.2	2 inpatient diagnoses or 1 inpatient and 1 outpatient diagnosis
	Other cardiac defects: septal defects, heterotaxy, pulmonary valve atresia, tricuspid stenosis, partial anomalous pulmonary venous return[d]	745.4, 745.8, 745.9, 746.00, 746.01, 746.1, 747.42, 759.3, 746.9	Q20.8, Q20.9, Q21.0, Q21.8, Q21.9, Q22.0, Q22.3, Q22.4, Q26.3, Q26.4, Q89.3	2 diagnoses
Orofacial/Respiratory	Choanal atresia	748.0	Q30.0	2 outpatient diagnoses

Organ system	Specific malformation group	ICD-9-CM diagnosis codes	ICD-10-CM diagnosis codes	(Primary definition) Modified algorithm
	Cleft lip and/or cleft palate	749.00–749.04, 749.10–749.14, 749.20–749.25	Q35.1–Q35.5, Q35.9, Q36.x, Q37.x	1 inpatient diagnosis or 2 outpatient diagnoses
Gastrointestinal	Biliary atresia	751.61	Q44.2	1 inpatient diagnosis or 2 outpatient diagnoses
	Intestinal atresia or stenosis	751.1, 751.2	Q41.x, Q42.x	1 inpatient diagnosis or 2 outpatient diagnoses
	Esophageal atresia with or without tracheoesophageal fistula	750.3	Q39.0–Q39.3	2 outpatient diagnoses or (1 inpatient and 1 outpatient diagnosis)
	Pyloric stenosis	750.5	Q40.0	1 inpatient diagnosis
	Bladder exstrophy	753.5	Q64.1x	1 inpatient diagnosis by 3 months of age and 1 outpatient diagnosis by 1 year
Genitourinary/Renal	Hypospadias	752.61	Q54.0–Q54.3, Q54.8, Q54.9	2 outpatient diagnoses; males only
	Renal dysplasia	753.15	Q61.4	2 outpatient diagnoses
	Renal agenesis or hypoplasia	753.0	Q60.0–Q60.6	1 inpatient diagnosis and 1 outpatient diagnosis
	Posterior urethral valve[e]	753.6	Q64.2	2 outpatient diagnoses; males only
	Prune belly[e]	756.71	Q79.4	2 outpatient diagnoses
Musculoskeletal[f]	Gastroschisis[g]	756.73	Q79.3	1 inpatient diagnosis or 2 outpatient diagnoses
	Omphalocele[g]	756.72	Q79.2	1 inpatient diagnosis by 3 months of age
	Congenital diaphragmatic hernia	756.6	Q79.0	1 inpatient diagnosis by 3 months of age
	Limb deficiency[h]	755.2–755.62, 755.64–755.9	Q71.0x–Q71.6x, Q71.89x, Q71.9x, Q72.0x–Q72.7x,	(1 inpatient or 2 outpatient diagnoses) and 1 diagnosis within 3 months

© 2025 Osmundson SS et al. *JAMA Network Open*.

Organ system	Specific malformation group	ICD-9-CM diagnosis codes	ICD-10-CM diagnosis codes	(Primary definition) Modified algorithm
			Q72.89x, Q72.9x, Q73.x	

Abbreviations: ICD-9-CM, International Classification of Diseases, Ninth Revision, Clinical Modification; ICD-10-CM, International Classification of Diseases, Tenth Revision, Clinical Modification.

[a] The original algorithm for each specific malformation group had a qualifying criterion of 1 diagnosis code and infant death in the first year of life. Our data source did not include death, so this criterion was not used. The algorithm citations are as follows: (1) Kharbanda EO, Vazquez-Benitez G, Romitti PA, et al. Identifying birth defects in automated data sources in the Vaccine Safety Datalink. *Pharmacoepidemiology and drug safety*. Apr 2017;26(4):412-420. doi:10.1002/pds.4153; and (2) Kharbanda EO, Vazquez-Benitez G, DeSilva MB, et al. Developing algorithms for identifying major structural birth defects using automated electronic health data. *Pharmacoepidemiology and drug safety*. Feb 2021;30(2):266-274. doi:10.1002/pds.5177. For code lists with full descriptions, see supplemental spreadsheet tab named eTable 29 in Supplement 2.
[b] Specific malformation group for microcephaly in the central nervous system was dropped due to inability to capture head circumference <5th percentile in claims data (Kharbanda et al. 2021).
[c] Severe cardiac category dropped anomalous congenital artery (746.85), since no ICD-10-CM equivalent. Removed ICD-9-CM codes for congenital pulmonary valve anomaly, unspecified (746.00), pulmonary valve atresia (746.01), and tricuspid stenosis (746.1). They were in the severe cardiac category in ICD-9-CM but conditions were considered other cardiac in ICD-10-CM.
[d] Added ICD-9-CM codes for congenital pulmonary valve anomaly, unspecified (746.00), pulmonary valve atresia (746.01), and tricuspid stenosis (746.1). These codes were in the severe cardiac category in ICD-9-CM but conditions were conditions were considered other cardiac in ICD-10-CM. Added ICD-9-CM 746.9 to match Q20.9 listed in other cardiac.
[e] ICD-9-CM category considered congenital hydronephrosis, posterior urethral valve, and prune belly as a single specific malformation group. ICD-10-CM only included posterior urethral valve. We kept posterior urethral valve as a separate category and added ICD-10-CM codes for prune belly (ICD-10-CM Q79.4) as a separate category for completeness. We dropped congenital hydronephrosis (ICD-9-CM 753.2x) because mild congenital hydronephrosis is common and does not often result in clinically important sequalae.
[f] ICD-9-CM musculoskeletal had a specific malformation group for sacral agenesis (ICD-9-CM 756.13), but it was not included in ICD-10-CM algorithm. There is no equivalent specific ICD-10-CM code, therefore the category was dropped.
[g] Gastroschisis/omphalocele were combined as a specific malformation group in ICD-9-CM but separate in ICD-10-CM. We split the ICD-9-CM codes into specific malformation groups for gastroschisis and omphalocele and dropped the vague code for other congenital anomalies of abdominal wall (756.79).
[h] We found an implausibly high absolute risk of limb deficiency when implementing the Kharbanda diagnosis code list. A very high proportion of the infants meeting this code were coded with a single ICD-9-CM code: 755.63, other congenital deformity of hip (joint). Based on clinical input, we dropped this code from our code list and suspect the coding might be related to fetal positioning during pregnancy.

© 2025 Osmundson SS et al. *JAMA Network Open*.

eTable 8. Codes to Identify Covariates[a,b]

Description	Timing	Code type(s)
**Demographics**		
Age of birthing parent	Index	N/A
Region of residence	Index	N/A
Urbanicity	Index	N/A
Year	Index	N/A
**Comorbidities**		
Pre-gestational diabetes or insulin	Baseline[c]	ICD-9-CM diagnosis<br>ICD-10-CM diagnosis<br>NDC
Chronic heart disease	Baseline[c]	ICD-9-CM diagnosis<br>ICD-10-CM diagnosis
Chronic hypertension	Baseline[c]	ICD-9-CM diagnosis<br>ICD-10-CM diagnosis
Obesity	Baseline[c]	ICD-9-CM diagnosis<br>ICD-10-CM diagnosis
Pelvic inflammatory disease or sexually transmitted infection	Baseline[c]	ICD-9-CM diagnosis<br>ICD-10-CM diagnosis
Alcohol use disorder	Baseline[c]	ICD-9-CM diagnosis<br>ICD-10-CM diagnosis
Other substance use disorder	Baseline[c]	ICD-9-CM diagnosis<br>ICD-10-CM diagnosis
Tobacco use	Baseline[c]	CPT<br>HCPCS<br>ICD-9-CM diagnosis<br>ICD-10-CM diagnosis
**Suspected teratogenic medication**		
Aminoglycoside	Days' supply overlapping the first trimester	NDC
Angiotensin-converting enzyme inhibitor	Days' supply overlapping the first trimester	NDC
Angiotensin receptor blocker	Days' supply overlapping the first trimester	NDC
Benzodiazepine	Days' supply overlapping the first trimester	NDC
Danazol	Days' supply overlapping the first trimester	NDC
Fluconazole[d]	Days' supply overlapping the first trimester	NDC
Folic acid antagonist	Days' supply overlapping the first trimester	HCPCS<br>NDC
Methimazole	Days' supply overlapping the first trimester	NDC
Molnupiravir	Days' supply overlapping the first trimester	NDC
Mycophenolate	Days' supply overlapping the first trimester	NDC
Potassium iodide	Days' supply overlapping the first trimester	NDC
Propylthiouracil	Days' supply overlapping the first trimester	NDC
Ribavirin	Days' supply overlapping the first trimester	NDC

© 2025 Osmundson SS et al. *JAMA Network Open*.

Description	Timing	Code type(s)
Selective serotonin reuptake inhibitor	Days' supply overlapping the first trimester	NDC
Statin	Days' supply overlapping the first trimester	NDC
Tetracycline	Days' supply overlapping the first trimester	NDC
**Other medications**		
Non-index antibiotic	First trimester	HCPCS<br>NDC
Oral antidiabetic	Baseline[c]	NDC
**Pre-pregnancy health care utilization intensity**		
Hospitalization	Pre-pregnancy[e]	MarketScan inpatient file
Emergency department visit	Pre-pregnancy[e]	CPT<br>UB-04 revenue code
No. outpatient office visits	Pre-pregnancy[e]	CPT
No. prescription therapeutic groups	Pre-pregnancy and during pregnancy[f]	N/A

Abbreviations: CPT, Current Procedural Terminology; HCPCS, Healthcare Common Procedure Coding System; ICD-9-CM, International Classification of Diseases, Ninth Revision, Clinical Modification; ICD-10-CM, International Classification of Diseases, Tenth Revision, Clinical Modification; N/A, not applicable; NDC, national drug code; UB-04, uniform billing.

[a] For code lists, see supplemental spreadsheet tab named eTable 30 in Supplement 2 for ICD-9-CM/ICD-10-CM diagnosis, CPT codes, HCPCS codes and UB-04 revenue codes; see tab named eTable 31 in Supplement 2 for medication names derived from NDC codes.
[b] All medications restricted to systemic routes of administration (i.e., buccal mucosa, implantation, injection, intradermal, intramuscular, intravenous, oral, oromucosal, subcutaneous, sublingual, transdermal).
[c] Baseline period for diagnosis codes was defined as last menstrual period date -180 through index antibiotic date. Baseline period for medications was defined as last menstrual period date -90 through index antibiotic date.
[d] For fluconazole, we required 12,000mg during exposure period using the dose, quantity, and days' supply of the prescription(s).
[e] Pre-pregnancy defined as last menstrual period (LMP) date -90 through LMP date.
[f] Pre-pregnancy and during pregnancy defined as LMP date -90 days through pregnancy outcome date; the index antibiotic prescription did not count.

© 2025 Osmundson SS et al. *JAMA Network Open*.

eTable 9. Distribution of Urinary Tract Infection-related Oral Antibiotic Combinations that Resulted in Exclusion from the Study Cohort[a]

First	Second	Third	Fourth	N
nitrofurantoin	β-lactam			3364
β-lactam	nitrofurantoin			2352
fluoroquinolone	β-lactam			353
TMP-SMX	nitrofurantoin			343
nitrofurantoin	TMP-SMX			338
TMP-SMX	β-lactam			337
fluoroquinolone	nitrofurantoin	β-lactam		333
fluoroquinolone	nitrofurantoin			305
TMP-SMX	nitrofurantoin	β-lactam		239
β-lactam	TMP-SMX			223
nitrofurantoin	fluoroquinolone			219
fluoroquinolone	β-lactam	nitrofurantoin		218
nitrofurantoin	TMP-SMX	β-lactam		179
TMP-SMX	β-lactam	nitrofurantoin		168
nitrofurantoin	fluoroquinolone	β-lactam		157
β-lactam	fluoroquinolone			154
β-lactam	TMP-SMX	nitrofurantoin		148
β-lactam	nitrofurantoin	TMP-SMX		146
β-lactam	fluoroquinolone	nitrofurantoin		141
nitrofurantoin	β-lactam	TMP-SMX		139
TMP-SMX	fluoroquinolone			111
β-lactam	nitrofurantoin	fluoroquinolone		75
TMP-SMX	fluoroquinolone	β-lactam		63
fluoroquinolone	TMP-SMX			50
nitrofurantoin	β-lactam	fluoroquinolone		47
fluoroquinolone	TMP-SMX	nitrofurantoin		45
TMP-SMX	fluoroquinolone	nitrofurantoin	β-lactam	36
fluoroquinolone	TMP-SMX	β-lactam	nitrofurantoin	33
fluoroquinolone	TMP-SMX	β-lactam		32
TMP-SMX	fluoroquinolone	nitrofurantoin		31
TMP-SMX	fluoroquinolone	β-lactam	nitrofurantoin	28
fluoroquinolone	nitrofurantoin	TMP-SMX		26
fluoroquinolone	TMP-SMX	nitrofurantoin	β-lactam	24
β-lactam	TMP-SMX	fluoroquinolone	nitrofurantoin	23

Abbreviations: TMP-SMX, trimethoprim/sulfamethoxazole.

© 2025 Osmundson SS et al. *JAMA Network Open*.

[a] N= 10,804 excluded for having >1 oral UTI-related antibiotic class during the first trimester. N=10,480 shown here, representing antibiotic combinations with at least 20 pregnancies.

© 2025 Osmundson SS et al. *JAMA Network Open*.

eTable 10. Distribution of UTI Indication Criterion Qualification among Individuals Treated for Urinary Tract Infection in the First Trimester of Pregnancy[a]

UTI indication criterion	N (%)
UTI diagnosis code	33,744 (47.1)
UTI-related symptom diagnosis code	6894 (9.6)
Urine culture and susceptibility test	44,320 (61.9)

Abbreviations: UTI, urinary tract infection.

[a] The criteria are not mutually exclusive, therefore a single pregnancy can qualify for the UTI indication based on >1 criterion.

© 2025 Osmundson SS et al. *JAMA Network Open*.

eTable 11. Distribution of Index Oral Antibiotic Agents among Individuals Treated for Urinary Tract Infection in the First Trimester of Pregnancy[a, b]

Antibiotic agent/class	Generic names	UTI cohort N= 71,604 N (%)
Nitrofurantoin	nitrofurantoin	42,402 (59.2)
Trimethoprim-sulfamethoxazole	sulfamethoxazole/trimethoprim	3494 (4.9)
Fluoroquinolone	ciprofloxacin	3394 (4.7)
	levofloxacin	267 (0.4)
	ofloxacin	2 (0.0)
β-lactam (cephalosporins and penicillins)	amoxicillin[a]	5248 (7.3)
	amoxicillin/clavulanate potassium	2476 (3.5)
	ampicillin[a]	2548 (3.6)
	cefaclor	32 (0.0)
	cefdinir	447 (0.6)
	cefpodoxime proxetil	149 (0.2)
	cephalexin	11,145 (15.6)

Abbreviations: UTI, urinary tract infection.

[a] Treatment guidelines specify that amoxicillin-alone and ampicillin-alone are not recommended for the treatment of urinary tract infection, however they are commonly used in practice.
[b] Distribution represents the first index oral antibiotic agent per pregnancy.

© 2025 Osmundson SS et al. *JAMA Network Open*.

eTable 12. Characteristics of Pregnancies Among Individuals Treated for Urinary Tract Infection in the First Trimester by Index Antibiotic Agent, After Propensity Score Weighting

Characteristic	Nitrofurantoin vs β-lactam		TMP-SMX vs β-lactam		Fluoroquinolone vs β-lactam	
	Weighted Nitrofurantoin (%)	Weighted β-lactam (%)	Weighted TMP-SMX (%)	Weighted β-lactam (%)	Weighted Fluoroquinolone (%)	Weighted β-lactam (%)
Sum of weights	22,051	22,042	22,263	22,032	22,363	22,014
Number of pregnancies	42,398	22,042	3494	22,032	3662	22,014
Number of birthing parents	41,961	21,925	3489	21,915	3660	21,897
Age of birthing parent at index, years						
15–22	3.6	3.7	3.8	3.7	3.3	3.7
23–29	36.5	36.6	36.1	36.6	35.3	36.6
30–34	37.4	37.2	37.4	37.2	37.7	37.2
35–39	18.8	18.8	19.1	18.8	19.3	18.8
40–49	3.6	3.7	3.5	3.7	4.3	3.6
Region of residence						
Northeast	15.4	15.2	15.5	15.2	15.2	15.2
Midwest	22.9	22.9	23.0	22.9	23.3	22.9
South	40.0	40.1	40.8	40.1	41.0	40.1
West	21.0	21.1	20.1	21.1	19.7	21.1
Unknown	0.7	0.7	0.7	0.7	0.7	0.7
Urbanicity						
Urban	84.1	84.2	83.5	84.1	83.4	84.1
Rural	10.8	10.7	11.5	10.7	11.1	10.7
Missing	5.1	5.2	5.0	5.2	5.5	5.2
Year of index antibiotic fill						
2006–2010	21.1	21.4	20.8	21.4	20.8	21.4
2011–2016	38.5	38.6	37.5	38.6	38.1	38.6
2017–2022	40.5	40.1	41.7	40.0	41.1	40.0
Comorbidities[a]						
Pre-gestational diabetes or insulin	1.6	1.6	1.6	1.6	2.1	1.6
Chronic heart disease	0.0	0.0	0.0	0.0	0.2	0.0
Chronic hypertension	3.2	3.2	3.2	3.2	3.5	3.2
Obesity	5.5	5.4	5.3	5.4	6.3	5.4

© 2025 Osmundson SS et al. *JAMA Network Open.*

Characteristic	Nitrofurantoin vs β-lactam		TMP-SMX vs β-lactam		Fluoroquinolone vs β-lactam	
	**Weighted Nitrofurantoin (%)**	**Weighted β-lactam (%)**	**Weighted TMP-SMX (%)**	**Weighted β-lactam (%)**	**Weighted Fluoroquinolone (%)**	**Weighted β-lactam (%)**
Pelvic inflammatory disease or sexually transmitted infection	14.3	14.3	17.1	14.3	16.7	14.2
Alcohol use disorder	0.2	0.1	0.2	0.1	0.2	0.1
Other substance use disorder	0.3	0.3	0.3	0.3	0.2	0.3
Tobacco use	2.4	2.4	2.5	2.4	2.0	2.4
Suspected teratogenic medication[b]						
Benzodiazepine	2.5	2.5	2.7	2.5	2.3	2.5
Fluconazole[c]	0.7	0.6	0.8	0.6	0.8	0.7
Folic acid antagonist	0.7	0.7	0.7	0.7	0.6	0.7
Selective serotonin reuptake inhibitor	7.2	7.3	6.7	7.3	6.4	7.3
Other suspected teratogen[d]	1.0	1.0	0.9	1.0	1.3	1.0
Other medications						
Non-index antibiotic[e]	10.6	10.6	12.4	10.5	11.4	10.6
Oral antidiabetic[a]	3.5	3.5	3.5	3.4	3.7	3.4
Pre-pregnancy health care utilization intensity[f]						
Any hospitalization	0.8	0.8	0.7	0.8	0.7	0.8
Any emergency department visit	6.5	6.6	6.5	6.6	6.2	6.6
No. of outpatient office visits, mean (SD)	1.3 (1.3)	1.3 (1.7)	1.3 (4.3)	1.3 (1.7)	1.3 (4.0)	1.3 (1.7)
No. of prescription therapeutic groups, mean (SD)	3.6 (1.7)	3.6 (2.4)	3.6 (5.9)	3.6 (2.4)	3.6 (5.9)	3.6 (2.4)

Abbreviations: SD, standard deviation; TMP-SMX, trimethoprim/sulfamethoxazole.

[a] Identified during the baseline period. For diagnosis codes, this was defined as last menstrual period date -180 through index antibiotic date. Baseline period for medications was defined as last menstrual period date -90 through index antibiotic date.
[b] Identified as days' supply overlapping the first trimester.
[c] 12,000 mg of fluconazole exposure during the exposure period was required based on prescription days' supply, dose, and quantity.
[d] Other suspected teratogenic medications were as follows: aminoglycoside, angiotensin-converting enzyme inhibitor, angiotensin receptor blocker, danazol, methimazole, molnupiravir, mycophenolate, potassium iodide, propylthiouracil, ribavirin, statin, tetracycline.
[e] Identified during the first trimester.
[f] Hospitalizations, ED visits, and outpatient office visits identified pre-pregnancy (LMP -90 days through LMP date). Prescriptions identified pre-pregnancy and during pregnancy defined as LMP date -90 days through pregnancy outcome date; the index antibiotic prescription did not count.

© 2025 Osmundson SS et al. *JAMA Network Open*.

eTable 13. Absolute Risk of Congenital Malformations Among Infants Born to Individuals Treated for Urinary tract infection in the First Trimester With an Antibiotic Agent by Organ-Specific Malformation Group and Outcome Definition

Organ system	Specific malformation group	No. of events (absolute risk per 1000)	
		Primary definition (≤ 365 days, Kharbanda algorithm)[a]	Sensitivity analysis (≤ 365 days, ≥ 1 code)[a]
Any congenital malformation		1518 (21.2)	2264 (31.6)
Cardiac	Severe cardiac defect: single ventricle, tricuspid atresia, ebstein anomaly, hypoplastic left heart, hypoplastic right heart, common truncus, transposition, atrioventricular septal defects, tetralogy of fallot, aortic valve atresia or stenosis, coarctation, total anomalous pulmonary venous return	190 (2.7)	292 (4.1)
	Other cardiac defect: septal defects, heterotaxy, pulmonary valve atresia, tricuspid stenosis, partial anomalous pulmonary venous return	655 (9.1)	917 (12.8)
	Any cardiac defect[b]	729 (10.2)	1049 (14.7)
Central nervous system	Neural tube defect: encephalocele, cranial meningocele, encephalomyelocele, spina bifida	42 (0.6)	56 (0.8)
	Holoprosencephaly	13 (0.2)	32 (0.4)
	Any central nervous system defect[b]	54 (0.8)	85 (1.2)
Eye	Anophthalmia, microphthalmia, cataracts and other lens defects	34 (0.5)	57 (0.8)
Ear	Anotia, microtia	9 (0.1)	22 (0.3)
Orofacial/Respiratory	Choanal atresia	8 (0.1)	18 (0.3)
	Cleft lip and/or cleft palate	95 (1.3)	103 (1.4)
	Any orofacial/respiratory defect[b]	103 (1.4)	121 (1.7)
Gastrointestinal	Biliary atresia	3 (0.0)	5 (0.1)
	Intestinal atresia or stenosis	79 (1.1)	90 (1.3)
	Esophageal atresia with or without tracheoesophageal fistula	15 (0.2)	20 (0.3)
	Pyloric stenosis	107 (1.5)	132 (1.8)
	Bladder exstrophy	2 (0.0)	5 (0.1)
	Any gastrointestinal defect[b]	202 (2.8)	247 (3.4)
Genitourinary/Renal	Hypospadias (male infants only)	284 (7.8)	475 (13.1)
	Renal dysplasia	21 (0.3)	37 (0.5)
	Renal agenesis or hypoplasia	29 (0.4)	85 (1.2)
	Posterior urethral valve (male infants only)	9 (0.2)	20 (0.6)
	Prune belly	0 (0.0)	0 (0.0)

© 2025 Osmundson SS et al. *JAMA Network Open*.

Organ system	Specific malformation group	No. of events (absolute risk per 1000)	
		Primary definition (≤ 365 days, Kharbanda algorithm)[a]	Sensitivity analysis (≤ 365 days, ≥ 1 code)[a]
	Any genitourinary/renal defect (male infants only)[b]	324 (8.9)	549 (15.1)
	Any genitourinary/renal defect[b]	341 (4.8)	607 (8.5)
Musculoskeletal	Gastroschisis	8 (0.1)	9 (0.1)
	Omphalocele	17 (0.2)	49 (0.7)
	Congenital diaphragmatic hernia	21 (0.3)	25 (0.3)
	Limb deficiency	73 (1.0)	129 (1.8)
	Any musculoskeletal defect[b]	117 (1.6)	207 (2.9)

[a] Primary definition per modified Kharbanda algorithm, which varied the number of diagnoses, settings, and timing of diagnoses depending on the organ system specific malformation group to qualify as a congenital malformation; claims queried included birthing parent claims (≤ 30 days after delivery) and infant claims (≤ 365 days after delivery); eTable 7. The sensitivity analysis definition included the same claims timeframe as the primary definition, but relaxed the algorithm to allow any single diagnosis code within the specific malformation group (after excluding diagnostic/rule-out codes, see eMethods) to qualify as a congenital malformation. Considering only enrollment up to a maximum of 365 days after birth, mean duration of insurance enrollment among infants was 300 days (standard deviation [SD] 109 days) for nitrofurantoin, 299 days (SD 109 days) for trimethoprim-sulfamethoxazole, 303 days (SD 107 days) for fluoroquinolones, and 296 days (SD 111 days) for β-lactams.

[b] Within each organ system, the "any" defect indicated that an infant met the algorithm for one or more specific malformation groups within the organ system.

© 2025 Osmundson SS et al. *JAMA Network Open*.

eTable 14. Absolute Risk of Congenital Malformations Among Infants Born to Individuals Treated for Urinary Tract Infection in the First Trimester by Index Antibiotic Agent[a,b]

Treatment	Total no. of pregnancies	Any congenital malformations		Any cardiac malformations	
		No. of events	Absolute risk per 1000 (95% CI)[c]	No. of events	Absolute risk per 1000 (95% CI)[c]
Nitrofurantoin	42,402	901	21.2 (19.9, 22.7)	437	10.3 (9.4, 11.3)
TMP-SMX	3494	94	26.9 (21.8, 32.8)	47	13.5 (9.9, 17.8)
Fluoroquinolone	3663	86	23.5 (18.8, 28.9)	38	10.4 (7.4, 14.2)
β-lactam	22,045	437	19.8 (18.0, 21.8)	207	9.4 (8.2, 10.8)

Abbreviations: CI, confidence interval; TMP-SMX, trimethoprim/sulfamethoxazole.

[a] The true risks of congenital malformations may be slightly underestimated, given a small proportion of fetuses with congenital malformations were not included in the cohort of live births due to spontaneous abortion, termination, or stillbirth.
[b] The absolute risk of congenital malformations by year ranged from 17.1 per 1000 (95% CI, 13.0, 22.2) in 2007 to 30.9 per 1000 (95% CI, 22.3, 41.5) in 2022.
[c] 95% confidence intervals calculated using the Clopper-Pearson method

© 2025 Osmundson SS et al. *JAMA Network Open*.

eTable 15. Crude and Weighted Risk Difference and Risk Ratio Estimates of Any Congenital Malformation in Infants Born to Individuals Treated for Urinary Tract Infection (UTI) in the First Trimester: Comparison of Different UTI-related Antibiotics Versus β-lactams (Reference)[a,b]

Outcome	RD per 1000 (95% CI) Untrimmed	RD per 1000 (95% CI) Trimmed & Weighted	RR (95% CI) Untrimmed	RR (95% CI) Trimmed & Weighted
**Primary analysis**				
Nitrofurantoin vs. β-lactam	1.43 (-0.87, 3.72)	2.41 (0.00, 4.82)	1.07 (0.96, 1.20)	1.12 (1.00, 1.26)
TMP-SMX vs. β-lactam	7.08 (1.41, 12.75)	6.88 (0.11, 13.65)	1.36 (1.09, 1.69)	1.35 (1.04, 1.75)
Fluoroquinolone vs. β-lactam	3.65 (-1.58, 8.89)	3.63 (-3.43, 10.70)	1.18 (0.94, 1.49)	1.18 (0.87, 1.60)
**Sensitivity analysis**				
Alternative reference group				
Nitrofurantoin vs. amoxicillin alone or cephalexin	2.72 (0.17, 5.27)	3.65 (0.98, 6.31)	1.15 (1.00, 1.31)	1.20 (1.04, 1.37)
TMP-SMX vs. amoxicillin alone or cephalexin	8.37 (2.60, 14.15)	9.02 (1.74, 16.30)	1.45 (1.15, 1.83)	1.49 (1.13, 1.97)
Fluoroquinolone vs. amoxicillin alone or cephalexin	4.95 (-0.40, 10.30)	4.80 (-2.52, 12.13)	1.27 (1.00, 1.61)	1.26 (0.91, 1.74)
Alternative outcome definition				
Nitrofurantoin vs. β-lactam	2.66 (-0.14, 5.46)	3.04 (0.14, 5.93)	1.09 (0.99, 1.20)	1.10 (1.00, 1.21)
TMP-SMX vs. β-lactam	8.29 (1.59, 15.00)	5.30 (-2.14, 12.73)	1.28 (1.07, 1.54)	1.18 (0.95, 1.47)
Fluoroquinolone vs. β-lactam	3.00 (-3.16, 9.16)	0.89 (-6.83, 8.62)	1.10 (0.91, 1.34)	1.03 (0.80, 1.33)
Restricted to symptomatic UTI				
Nitrofurantoin vs. β-lactam	0.44 (-3.14, 4.02)	1.42 (-2.38, 5.21)	1.02 (0.86, 1.22)	1.07 (0.89, 1.28)
TMP-SMX vs. β-lactam	7.28 (0.07, 14.49)	7.66 (-1.40, 16.71)	1.36 (1.03, 1.80)	1.38 (0.98, 1.94)
Fluoroquinolone vs. β-lactam	3.87 (-2.25, 10.00)	4.27 (-4.57, 13.11)	1.19 (0.91, 1.55)	1.21 (0.84, 1.75)
Restricted to ICD-10 era				
Nitrofurantoin vs. β-lactam	4.79 (1.30, 8.28)	5.35 (1.72, 8.97)	1.25 (1.06, 1.47)	1.28 (1.08, 1.51)
TMP-SMX vs. β-lactam	12.20 (0.96, 23.44)	10.27 (-0.90, 21.44)	1.63 (1.12, 2.36)	1.54 (1.04, 2.29)
Fluoroquinolone vs. β-lactam	3.75 (-6.35, 13.86)	1.85 (-9.10, 12.80)	1.19 (0.77, 1.86)	1.09 (0.65, 1.83)
Alternative exposure window 2–13 weeks[c]				
Nitrofurantoin vs. β-lactam	1.56 (-0.77, 3.90)	2.55 (0.09, 5.01)	1.08 (0.96, 1.21)	1.13 (1.00, 1.27)

© 2025 Osmundson SS et al. *JAMA Network Open.*

Outcome	RD per 1000 (95% CI) Untrimmed	RD per 1000 (95% CI) Trimmed & Weighted	RR (95% CI) Untrimmed	RR (95% CI) Trimmed & Weighted
TMP-SMX vs. β-lactam	5.47 (-0.87, 11.80)	4.20 (-2.86, 11.27)	1.28 (0.99, 1.65)	1.21 (0.90, 1.64)
FQ vs. β-lactam	4.71 (-1.89, 11.30)	2.94 (-5.15, 11.02)	1.24 (0.94, 1.63)	1.15 (0.80, 1.64)
Adjusted for gestational age at exposure				
Nitrofurantoin vs. β-lactam	1.43 (-0.87, 3.72)	2.34 (-0.07, 4.75)	1.07 (0.96, 1.20)	1.12 (1.00, 1.26)
TMP-SMX vs. β-lactam	7.08 (1.41, 12.75)	6.08 (-1.46, 13.62)	1.36 (1.09, 1.69)	1.31 (0.97, 1.76)
FQ vs. β-lactam	3.65 (-1.58, 8.89)	14.43 (-8.96, 37.81)	1.18 (0.94, 1.49)	1.73 (0.87, 3.44)

Abbreviations: ICD-10-CM, International Classification of Diseases, Tenth Revision, Clinical Modification; RR, risk ratio; RD, risk difference; TMP-SMX, trimethoprim-sulfamethoxazole; UTI, urinary tract infection.

[a] Propensity score weighted analysis accounted for all potential confounders listed in eTable 8. We required ≥5 events in each exposure group to estimate the treatment effect.
[b] RD and RR estimates and accompanying 95% CIs were very similar (and often identical) in the untrimmed/unweighted population and trimmed/unweighted population (data not shown).
[c] 2-13 weeks gestation defined as days 14-97 of gestation.

© 2025 Osmundson SS et al. *JAMA Network Open*.

eTable 16. Crude and Weighted Risk Difference and Risk Ratio Estimates of Any Cardiac Malformation in Infants Born to Individuals Treated for Urinary Tract Infection (UTI) in the First Trimester: Comparison of Different UTI-related Antibiotics Versus β-lactams (Reference)[a]

Outcome	RD per 1000 (95% CI) Untrimmed	RD per 1000 (95% CI) Trimmed & Weighted	RR (95% CI) Untrimmed	RR (95% CI) Trimmed & Weighted
**Primary analysis**				
Nitrofurantoin vs. β-lactam	0.92 (-0.68, 2.51)	1.44 (-0.24, 3.11)	1.10 (0.93, 1.29)	1.15 (0.97, 1.37)
TMP-SMX vs. β-lactam	4.06 (0.04, 8.09)	4.20 (-0.71, 9.11)	1.43 (1.05, 1.96)	1.45 (1.00, 2.10)
Fluoroquinolone vs. β-lactam	0.98 (-2.53, 4.50)	2.15 (-3.15, 7.45)	1.10 (0.78, 1.56)	1.23 (0.77, 1.96)
**Sensitivity analysis**				
Alternative reference group				
Nitrofurantoin vs. amoxicillin alone or cephalexin	1.27 (-0.51, 3.06)	1.74 (-0.13, 3.61)	1.14 (0.94, 1.38)	1.19 (0.98, 1.45)
TMP-SMX vs. amoxicillin alone or cephalexin	4.42 (0.31, 8.52)	4.97 (-0.35, 10.30)	1.49 (1.07, 2.07)	1.56 (1.04, 2.33)
Fluoroquinolone vs. amoxicillin alone or cephalexin	1.34 (-2.27, 4.95)	2.43 (-3.07, 7.94)	1.15 (0.80, 1.64)	1.27 (0.78, 2.07)
Alternative outcome definition				
Nitrofurantoin vs. β-lactam	1.46 (-0.46, 3.39)	1.68 (-0.32, 3.67)	1.11 (0.97, 1.27)	1.12 (0.98, 1.29)
TMP-SMX vs. β-lactam	2.95 (-1.56, 7.45)	2.19 (-3.01, 7.38)	1.22 (0.92, 1.61)	1.16 (0.83, 1.62)
Fluoroquinolone vs. β-lactam	-0.28 (-4.30, 3.75)	-0.07 (-5.59, 5.46)	0.98 (0.73, 1.32)	1.00 (0.66, 1.49)
Restricted to symptomatic UTI				
Nitrofurantoin vs. β-lactam	0.05 (-2.38, 2.47)	0.40 (-2.17, 2.97)	1.00 (0.78, 1.30)	1.04 (0.80, 1.37)
TMP-SMX vs. β-lactam	2.50 (-2.29, 7.30)	1.51 (-3.83, 6.86)	1.27 (0.83, 1.94)	1.16 (0.70, 1.93)
Fluoroquinolone vs. β-lactam	2.22 (-2.02, 6.47)	3.58 (-3.05, 10.21)	1.24 (0.84, 1.82)	1.38 (0.81, 2.35)
Restricted to ICD-10 era				
Nitrofurantoin vs. β-lactam	1.13 (-1.34, 3.60)	1.84 (-0.77, 4.44)	1.11 (0.88, 1.40)	1.18 (0.93, 1.50)
TMP-SMX vs. β-lactam	7.14 (-1.23, 15.51)	6.48 (-1.83, 14.78)	1.70 (1.03, 2.82)	1.66 (0.98, 2.83)
Fluoroquinolone vs. β-lactam	1.94 (-5.41, 9.29)	2.59 (-6.18, 11.35)	1.19 (0.64, 2.20)	1.25 (0.63, 2.49)
Alternative exposure window (4–9 weeks)[b]				
Nitrofurantoin vs. β-lactam	-0.12 (-2.37, 2.12)	0.30 (-2.10, 2.69)	0.99 (0.79, 1.23)	1.03 (0.82, 1.29)
TMP-SMX vs. β-lactam	1.20 (-5.55, 7.94)	-0.83 (-7.33, 5.67)	1.12 (0.62, 2.01)	0.92 (0.47, 1.81)
Fluoroquinolone vs. β-lactam	1.70 (-6.34, 9.74)	7.04 (-6.46, 20.55)	1.16 (0.59, 2.28)	1.69 (0.76, 3.74)

© 2025 Osmundson SS et al. *JAMA Network Open.*

Outcome	RD per 1000 (95% CI) Untrimmed	RD per 1000 (95% CI) Trimmed & Weighted	RR (95% CI) Untrimmed	RR (95% CI) Trimmed & Weighted
Adjusted for gestational age at exposure				
Nitrofurantoin vs. β-lactam	0.92 (-0.68, 2.51)	1.43 (-0.24, 3.11)	1.10 (0.93, 1.29)	1.15 (0.97, 1.37)
TMP-SMX vs. β-lactam	4.06 (0.04, 8.09)	6.15 (-0.13, 12.44)	1.43 (1.05, 1.96)	1.66 (1.09, 2.52)
Fluoroquinolone vs. β-lactam	0.98 (-2.53, 4.50)	8.80 (-9.65, 27.26)	1.10 (0.78, 1.56)	1.93 (0.70, 5.36)

Abbreviations: ICD-10-CM, International Classification of Diseases, Tenth Revision, Clinical Modification; RR, risk ratio; RD, risk difference; TMP-SMX, trimethoprim-sulfamethoxazole; UTI, urinary tract infection.

[a] Propensity score weighted analysis accounted for all potential confounders listed in eTable 8. We required ≥5 events in each exposure group to estimate the treatment effect.
[b] 4-9 weeks gestation defined as days 28-69 of gestation.

© 2025 Osmundson SS et al. *JAMA Network Open*.

eTable 17. Crude and Weighted Risk Difference and Risk Ratio Estimates of Other Organ-Specific Malformations in Infants Born to Individuals Treated for Urinary Tract Infection (UTI) in the First Trimester: Comparison of Different UTI-related Antibiotics Versus β-lactams (Reference)[a]

Outcome	RD per 1000 (95% CI) Untrimmed	RD per 1000 (95% CI) Trimmed & Weighted	RR (95% CI) Untrimmed	RR (95% CI) Trimmed & Weighted
**Central nervous system**				
Nitrofurantoin vs. β-lactam	-0.07 (-0.51, 0.38)	-0.05 (-0.51, 0.42)	0.91 (0.48, 1.72)	0.93 (0.49, 1.80)
TMP-SMX vs. β-lactam	NE	NE	NE	NE
Fluoroquinolone vs. β-lactam	1.73 (0.08, 3.38)	1.01 (-0.61, 2.63)	3.39 (1.47, 7.80)	2.39 (0.84, 6.80)
**Orofacial/Respiratory malformations**				
Nitrofurantoin vs. β-lactam	0.03 (-0.56, 0.62)	0.16 (-0.48, 0.79)	1.02 (0.65, 1.60)	1.12 (0.70, 1.78)
TMP-SMX vs. β-lactam	1.83 (-0.09, 3.75)	2.49 (-0.24, 5.23)	2.39 (1.20, 4.79)	2.89 (1.31, 6.41)
Fluoroquinolone vs. β-lactam	0.32 (-1.07, 1.72)	0.90 (-1.62, 3.42)	1.25 (0.52, 3.00)	1.68 (0.52, 5.43)
**Gastrointestinal malformations**				
Nitrofurantoin vs. β-lactam	0.15 (-0.70, 1.00)	0.02 (-0.84, 0.87)	1.06 (0.77, 1.44)	1.01 (0.73, 1.39)
TMP-SMX vs. β-lactam	0.47 (-1.51, 2.45)	-0.74 (-2.21, 0.72)	1.18 (0.62, 2.24)	0.72 (0.35, 1.48)
Fluoroquinolone vs. β-lactam	0.60 (-1.37, 2.57)	1.76 (-1.46, 4.98)	1.22 (0.66, 2.27)	1.66 (0.78, 3.52)
**Genitourinary malformations**				
Nitrofurantoin vs. β-lactam	-0.14 (-1.27, 1.00)	0.43 (-0.78, 1.65)	0.97 (0.77, 1.23)	1.09 (0.86, 1.38)
TMP-SMX vs. β-lactam	-0.03 (-2.52, 2.45)	0.64 (-2.56, 3.85)	0.99 (0.60, 1.65)	1.13 (0.63, 2.03)
Fluoroquinolone vs. β-lactam	-1.08 (-3.28, 1.12)	-1.72 (-4.36, 0.92)	0.78 (0.45, 1.36)	0.65 (0.29, 1.44)
**Musculoskeletal malformations**				
Nitrofurantoin vs. β-lactam	0.31 (-0.31, 0.94)	0.15 (-0.46, 0.76)	1.23 (0.80, 1.88)	1.11 (0.72, 1.72)
TMP-SMX vs. β-lactam	0.36 (-1.10, 1.81)	-0.38 (-1.35, 0.58)	1.26 (0.53, 3.03)	0.71 (0.27, 1.87)
Fluoroquinolone vs. β-lactam	1.37 (-0.39, 3.13)	1.42 (-1.15, 3.99)	2.01 (0.98, 4.10)	2.04 (0.77, 5.41)

Abbreviations: NE, not estimable; RR, risk ratio; RD, risk difference; TMP-SMX, trimethoprim-sulfamethoxazole.

[a] Propensity score weighted analysis accounted for all potential confounders listed in eTable 8. We required ≥5 events in each exposure group to estimate the treatment effect.

© 2025 Osmundson SS et al. *JAMA Network Open*.

eTable 18. Crude and Weighted Risk Difference and Risk Ratio Estimates of Specific Malformation Groups in Infants Born to Individuals Treated for Urinary Tract Infection (UTI) in the First Trimester: Comparison of Different UTI-related Antibiotics Versus β-lactams (Reference)[a]

Outcome	RD per 1000 (95% CI) Untrimmed	RD per 1000 (95% CI) Trimmed & Weighted	RR (95% CI) Untrimmed	RR (95% CI) Trimmed & Weighted
**Severe cardiac defects**				
Nitrofurantoin vs. β-lactam	0.36 (-0.46, 1.17)	0.49 (-0.37, 1.35)	1.15 (0.83, 1.59)	1.20 (0.86, 1.68)
TMP-SMX vs. β-lactam	2.18 (-0.16, 4.51)	2.62 (-0.40, 5.64)	1.90 (1.09, 3.33)	2.09 (1.09, 3.99)
Fluoroquinolone vs. β-lactam	NE	NE	NE	NE
**Other cardiac defects**				
Nitrofurantoin vs. β-lactam	0.90 (-0.61, 2.41)	1.22 (-0.36, 2.80)	1.11 (0.93, 1.32)	1.15 (0.96, 1.37)
TMP-SMX vs. β-lactam	3.96 (0.11, 7.81)	4.32 (-0.48, 9.11)	1.47 (1.06, 2.05)	1.52 (1.02, 2.25)
Fluoroquinolone vs. β-lactam	1.48 (-1.93, 4.89)	2.38 (-2.76, 7.51)	1.18 (0.83, 1.68)	1.28 (0.79, 2.09)
**Neural tube defects**				
Nitrofurantoin vs. β-lactam	-0.30 (-0.73, 0.12)	-0.26 (-0.70, 0.18)	0.58 (0.29, 1.17)	0.64 (0.31, 1.32)
TMP-SMX vs. β-lactam	NE	NE	NE	NE
Fluoroquinolone vs. β-lactam	1.19 (-0.28, 2.65)	0.64 (-0.89, 2.17)	2.63 (1.07, 6.50)	1.88 (0.56, 6.26)
**Cleft lip and/or cleft palate**				
Nitrofurantoin vs. β-lactam	0.05 (-0.52, 0.61)	0.14 (-0.46, 0.74)	1.04 (0.65, 1.66)	1.12 (0.69, 1.82)
TMP-SMX vs. β-lactam	1.97 (0.06, 3.88)	2.63 (-0.10, 5.36)	2.67 (1.32, 5.40)	3.23 (1.44, 7.22)
Fluoroquinolone vs. β-lactam	0.46 (-0.93, 1.84)	1.04 (-1.48, 3.55)	1.39 (0.57, 3.37)	1.88 (0.58, 6.10)
**Intestinal atresia or stenosis**				
Nitrofurantoin vs. β-lactam	0.07 (-0.47, 0.60)	-0.04 (-0.57, 0.49)	1.06 (0.65, 1.75)	0.96 (0.57, 1.61)
TMP-SMX vs. β-lactam	NE	NE	NE	NE
Fluoroquinolone vs. β-lactam	0.59 (-0.78, 1.97)	0.54 (-0.83, 1.91)	1.57 (0.64, 3.85)	1.52 (0.61, 3.80)
**Pyloric stenosis**				
Nitrofurantoin vs. β-lactam	0.22 (-0.39, 0.82)	0.16 (-0.45, 0.77)	1.17 (0.75, 1.80)	1.12 (0.72, 1.75)
TMP-SMX vs. β-lactam	0.69 (-0.87, 2.25)	-0.07 (-1.24, 1.10)	1.52 (0.67, 3.47)	0.95 (0.37, 2.40)
Fluoroquinolone vs. β-lactam	0.32 (-1.07, 1.72)	1.54 (-1.37, 4.45)	1.25 (0.52, 3.00)	2.17 (0.75, 6.31)
**Hypospadias (male infants only)**				

© 2025 Osmundson SS et al. *JAMA Network Open.*

Outcome	RD per 1000 (95% CI) Untrimmed	RD per 1000 (95% CI) Trimmed & Weighted	RR (95% CI) Untrimmed	RR (95% CI) Trimmed & Weighted
Nitrofurantoin vs. β-lactam	0.60 (-1.40, 2.61)	1.50 (-0.65, 3.65)	1.08 (0.83, 1.40)	1.20 (0.92, 1.56)
TMP-SMX vs. β-lactam	0.26 (-4.11, 4.64)	1.05 (-4.50, 6.60)	1.03 (0.59, 1.82)	1.14 (0.59, 2.19)
Fluoroquinolone vs. β-lactam	-1.68 (-5.48, 2.13)	-2.29 (-7.22, 2.65)	0.78 (0.42, 1.45)	0.70 (0.28, 1.73)
**Limb deficiency**				
Nitrofurantoin vs. β-lactam	0.06 (-0.44, 0.56)	-0.09 (-0.56, 0.39)	1.07 (0.62, 1.82)	0.90 (0.53, 1.55)
TMP-SMX vs. β-lactam	0.81 (-0.62, 2.24)	0.07 (-0.85, 0.99)	1.89 (0.76, 4.71)	1.08 (0.40, 2.95)
Fluoroquinolone vs. β-lactam	0.73 (-0.64, 2.10)	0.08 (-0.82, 0.97)	1.81 (0.73, 4.49)	1.09 (0.43, 2.74)

Abbreviations: NE, not estimable; RR, risk ratio; RD, risk difference; TMP-SMX, trimethoprim-sulfamethoxazole.

[a] Propensity score weighted analysis accounted for all potential confounders listed in eTable 8. We required ≥5 events in each exposure group to estimate the treatment effect.

© 2025 Osmundson SS et al. *JAMA Network Open*.

eTable 19. E-values and Associated Weighted Risk Ratio Estimates of Any Congenital Malformation, Any Cardiac Malformation, Other Organ-Specific Congenital Malformations, and Specific Congenital Malformations in Infants Born to Individuals Treated for Urinary Tract Infection (UTI) in the First Trimester: Comparison of Different UTI-related Antibiotics Versus β-lactams (Reference)[a]

	Weighted RR (95% CI)	E-value (Point estimate)	E-value (Confidence limit[b])
**Any congenital malformation**			
Nitrofurantoin vs. β-lactam	1.12 (1.00, 1.26)	1.49	N/A
TMP-SMX vs. β-lactam	1.35 (1.04, 1.75)	2.03	1.24
Fluoroquinolone vs. β-lactam	1.18 (0.87, 1.60)	1.65	N/A
**Any cardiac malformation**			
Nitrofurantoin vs. β-lactam	1.15 (0.97, 1.37)	1.57	N/A
TMP-SMX vs. β-lactam	1.45 (1.00, 2.10)	2.25	N/A
Fluoroquinolone vs. β-lactam	1.23 (0.77, 1.96)	1.76	N/A
**Central nervous system**			
Nitrofurantoin vs. β-lactam	0.93 (0.49, 1.80)	1.35	N/A
TMP-SMX vs. β-lactam	NE	NE	NE
FQ vs. β-lactam	2.39 (0.84, 6.80)	4.21	N/A
**Orofacial/Respiratory malformations**			
Nitrofurantoin vs. β-lactam	1.12 (0.70, 1.78)	1.48	N/A
TMP-SMX vs. β-lactam	2.89 (1.31, 6.41)	5.24	1.94
FQ vs. β-lactam	1.68 (0.52, 5.43)	2.75	N/A
**Gastrointestinal malformations**			
Nitrofurantoin vs. β-lactam	1.01 (0.73, 1.39)	1.09	N/A
TMP-SMX vs. β-lactam	0.72 (0.35, 1.48)	2.12	N/A
FQ vs. β-lactam	1.66 (0.78, 3.52)	2.70	N/A
**Genitourinary/Renal malformations**			
Nitrofurantoin vs. β-lactam	1.09 (0.86, 1.38)	1.40	N/A
TMP-SMX vs. β-lactam	1.13 (0.63, 2.03)	1.52	N/A
FQ vs. β-lactam	0.65 (0.29, 1.44)	2.45	N/A
**Musculoskeletal malformations**			
Nitrofurantoin vs. β-lactam	1.11 (0.72, 1.72)	1.46	N/A
TMP-SMX vs. β-lactam	0.71 (0.27, 1.87)	2.17	N/A
FQ vs. β-lactam	2.04 (0.77, 5.41)	3.50	N/A
**Severe cardiac defects**			
Nitrofurantoin vs. β-lactam	1.20 (0.86, 1.68)	1.70	N/A
TMP-SMX vs. β-lactam	2.09 (1.09, 3.99)	3.60	1.42
FQ vs. β-lactam	NE	NE	NE

© 2025 Osmundson SS et al. *JAMA Network Open.*

	Weighted RR (95% CI)	E-value (Point estimate)	E-value (Confidence limit[b])
**Other cardiac defects**			
Nitrofurantoin vs. β-lactam	1.15 (0.96, 1.37)	1.55	N/A
TMP-SMX vs. β-lactam	1.52 (1.02, 2.25)	2.40	1.18
FQ vs. β-lactam	1.28 (0.79, 2.09)	1.89	N/A
**Neural tube defects**			
Nitrofurantoin vs. β-lactam	0.64 (0.31, 1.32)	2.50	N/A
TMP-SMX vs. β-lactam	NE	NE	NE
FQ vs. β-lactam	1.88 (0.56, 6.26)	3.16	N/A
**Cleft lip and/or cleft palate**			
Nitrofurantoin vs. β-lactam	1.12 (0.69, 1.82)	1.48	N/A
TMP-SMX vs. β-lactam	3.23 (1.44, 7.22)	5.91	2.24
FQ vs. β-lactam	1.88 (0.58, 6.10)	3.16	N/A
**Intestinal atresia or stenosis**			
Nitrofurantoin vs. β-lactam	0.96 (0.57, 1.61)	1.25	N/A
TMP-SMX vs. β-lactam	NE	NE	NE
FQ vs. β-lactam	1.52 (0.61, 3.80)	2.40	N/A
**Pyloric stenosis**			
Nitrofurantoin vs. β-lactam	1.12 (0.72, 1.75)	1.49	N/A
TMP-SMX vs. β-lactam	0.95 (0.37, 2.40)	1.30	N/A
FQ vs. β-lactam	2.17 (0.75, 6.31)	3.76	N/A
**Hypospadias (male infants only)**			
Nitrofurantoin vs. β-lactam	1.20 (0.92, 1.56)	1.69	N/A
TMP-SMX vs. β-lactam	1.14 (0.59, 2.19)	1.54	N/A
FQ vs. β-lactam	0.70 (0.28, 1.73)	2.22	N/A
**Limb deficiency**			
Nitrofurantoin vs. β-lactam	0.90 (0.53, 1.55)	1.45	N/A
TMP-SMX vs. β-lactam	1.08 (0.40, 2.95)	1.38	N/A
FQ vs. β-lactam	1.09 (0.43, 2.74)	1.39	N/A

Abbreviations: ICD-10-CM, International Classification of Diseases, Tenth Revision, Clinical Modification; N/A, not applicable; NE, not estimable; RR, risk ratio; TMP-SMX, trimethoprim-sulfamethoxazole.

[a] Propensity score weighted analysis accounted for all potential confounders listed in eTable 8. We required ≥5 events in each exposure group to estimate the treatment effect.
[b] E-value for the limit of the 95% confidence interval closest to the null, i.e., the lower limit for risk ratios > 1 and the upper limit for risk ratios < 1. Treatment effect estimates whose confidence intervals include 1 were assigned an E-value for the limit of 'N/A' to indicate that no confounding was needed to nullify the effect.

© 2025 Osmundson SS et al. *JAMA Network Open*.

eTable 20. Characteristics of Pregnancies Among Individuals Treated with a UTI-related Antibiotic for Any Indication in the First Trimester by Index Antibiotic Agent (N=256,686)

Characteristic	Nitrofurantoin N (%)	TMP-SMX N (%)	Fluoroquinolone N (%)	β-lactam N (%)
Number of pregnancies	79,827	8963	9970	157,926
Number of birthing parents	78,642	8944	9948	154,693
Age of birthing parent at index, years				
15–22	3163 (4.0)	460 (5.1)	338 (3.4)	4426 (2.8)
23–29	31,216 (39.1)	3620 (40.4)	3669 (36.8)	56,242 (35.6)
30–34	29,282 (36.7)	3109 (34.7)	3657 (36.7)	62,167 (39.4)
35–39	13,522 (16.9)	1486 (16.6)	1897 (19.0)	29,774 (18.9)
40–49	2644 (3.3)	288 (3.2)	409 (4.1)	5317 (3.4)
Region of residence				
Northeast	10,506 (13.2)	1056 (11.8)	1413 (14.2)	23,076 (14.6)
Midwest	16,038 (20.1)	2152 (24.0)	2090 (21.0)	38,329 (24.3)
South	37,382 (46.8)	4211 (47.0)	4629 (46.4)	68,509 (43.4)
West	15,156 (19.0)	1454 (16.2)	1731 (17.4)	26,610 (16.9)
Unknown	745 (0.9)	90 (1.0)	107 (1.1)	1402 (0.9)
Urbanicity				
Urban	68,556 (85.9)	7334 (81.8)	8642 (86.7)	132,219 (83.7)
Rural	8440 (10.6)	1348 (15.0)	1084 (10.9)	19,196 (12.2)
Missing	2831 (3.5)	281 (3.1)	244 (2.5)	6511 (4.1)
Year of index antibiotic fill				
2006–2010	27,521 (34.5)	3661 (40.9)	4352 (43.6)	51,582 (32.7)
2011–2016	33,968 (42.5)	3735 (41.7)	4439 (44.5)	64,617 (40.9)
2017–2022	18,338 (23.0)	1567 (17.5)	1179 (11.8)	41,727 (26.4)
Gestational age of index antibiotic fill, weeks				
0–2	5899 (7.4)	3805 (42.5)	5731 (57.5)	26,500 (16.8)
3–5	9133 (11.4)	2119 (23.6)	2908 (29.2)	27,386 (17.3)
6–8	21,387 (26.8)	1045 (11.7)	674 (6.8)	37,046 (23.5)
9–11	28,268 (35.4)	1173 (13.1)	416 (4.2)	42,628 (27.0)
12–13	15,140 (19.0)	821 (9.2)	241 (2.4)	24,366 (15.4)
Comorbidities[a]				
Pre-gestational diabetes or insulin	1171 (1.5)	157 (1.8)	155 (1.6)	2170 (1.4)
Chronic heart disease	16 (0.0)	5 (0.1)	3 (0.0)	44 (0.0)
Chronic hypertension	2265 (2.8)	289 (3.2)	310 (3.1)	4875 (3.1)

© 2025 Osmundson SS et al. *JAMA Network Open*.

Characteristic	Nitrofurantoin N (%)	TMP-SMX N (%)	Fluoroquinolone N (%)	β-lactam N (%)
Obesity	3075 (3.9)	380 (4.2)	316 (3.2)	6797 (4.3)
Pelvic inflammatory disease or sexually transmitted infection	9229 (11.6)	771 (8.6)	846 (8.5)	11,956 (7.6)
Alcohol use disorder	123 (0.1)	22 (0.2)	20 (0.2)	235 (0.1)
Other substance use disorder	173 (0.2)	27 (0.3)	31 (0.3)	336 (0.2)
Tobacco use	1363 (1.7)	207 (2.3)	164 (1.6)	3052 (1.9)
Suspected teratogenic medication[b]				
Benzodiazepine	1909 (2.4)	299 (3.3)	500 (5.0)	4783 (3.0)
Fluconazole[c]	591 (0.7)	180 (2.0)	246 (2.5)	1589 (1.0)
Folic acid antagonist	499 (0.6)	72 (0.8)	106 (1.1)	1110 (0.7)
Selective serotonin reuptake inhibitor	5013 (6.3)	714 (8.0)	829 (8.3)	12,932 (8.2)
Other suspected teratogen[d]	767 (1.0)	127 (1.4)	166 (1.7)	1712 (1.1)
Other medications				
Non-index antibiotic[e]	8186 (10.2)	1317 (14.7)	2016 (20.2)	19,860 (12.6)
Oral antidiabetic[a]	2766 (3.5)	299 (3.3)	330 (3.3)	5391 (3.4)
Pre-pregnancy health care utilization intensity[f]				
Any hospitalization	618 (0.8)	95 (1.1)	109 (1.1)	1278 (0.8)
Any emergency department visit	4949 (6.2)	631 (7.0)	687 (6.9)	9082 (5.8)
No. of outpatient office visits, mean (SD)	1.2 (1.7)	1.3 (1.6)	1.5 (1.8)	1.3 (1.7)
No. of prescription therapeutic groups, mean (SD)	3.6 (2.3)	3.9 (2.3)	4.2 (2.4)	3.9 (2.4)
Possible antibiotic indication[g]				
Abdominal infection	43 (0.0)	26 (0.3)	119 (1.2)	200 (0.1)
Dental infection	7 (0.0)	8 (0.1)	3 (0.0)	307 (0.2)
Lower respiratory infection	64 (0.1)	70 (0.8)	356 (3.6)	3742 (2.4)
Upper respiratory infection	891 (1.1)	677 (7.6)	1280 (12.8)	54,655 (34.6)
Pyelonephritis	121 (0.1)	30 (0.3)	194 (2.0)	242 (0.1)
Skin and soft tissue infection	44 (0.1)	894 (10.0)	99 (1.0)	3662 (2.3)
Urinary tract infection, inpatient	11 (0.0)	1 (0.0)	0 (0.0)	10 (0.0)
Urinary tract infection, outpatient	42,402 (53.1)	3494 (39.0)	3663 (36.7)	22,045 (14.0)
None of the above	36,959 (46.3)	3944 (44.0)	4622 (46.4)	76,109 (48.2)

Abbreviations: SD, standard deviation; TMP-SMX, trimethoprim-sulfamethoxazole; UTI, urinary tract infection.

[a] Identified during the baseline period. For diagnosis codes, this was defined as last menstrual period date -180 through index antibiotic date. Baseline period for medications was defined as last menstrual period date -90 through index antibiotic date.

[b] Identified as days' supply overlapping the first trimester.

© 2025 Osmundson SS et al. *JAMA Network Open*.

[c] 12,000 mg of fluconazole exposure during the exposure period was required based on prescription days' supply, dose, and quantity.

[d] Other suspected teratogenic medications were as follows: aminoglycoside, angiotensin-converting enzyme inhibitor, angiotensin receptor blocker, danazol, methimazole, molnupiravir, mycophenolate, potassium iodide, propylthiouracil, ribavirin, statin, tetracycline.

[e] Identified during the first trimester.

[f] Hospitalizations, ED visits, and outpatient office visits identified pre-pregnancy (LMP-90 days through LMP date). Prescriptions identified pre-pregnancy and during pregnancy defined as LMP date-90 days through pregnancy outcome date; the index antibiotic prescription did not count.

[g] Identified ± 7 days from the index antibiotic date.

© 2025 Osmundson SS et al. *JAMA Network Open*.

eTable 21. Characteristics of Pregnancies Among Individuals Treated with a UTI-related Antibiotic for Any Indication in the First Trimester by Index Antibiotic Agent, After Propensity Score Weighting

Characteristic	Nitrofurantoin vs β-lactam		TMP-SMX vs β-lactam		Fluoroquinolone vs β-lactam	
	Weighted Nitrofurantoin (%)	Weighted β-lactam (%)	Weighted TMP-SMX (%)	Weighted β-lactam (%)	Weighted Fluoroquinolone (%)	Weighted β-lactam (%)
Sum of weights	158,025	157,915	157,693	157,757	157,241	157,884
Number of pregnancies	79,826	157,915	8962	157,757	9970	157,884
Number of birthing parents	78,641	154,682	8943	154,528	9948	154,651
Age of birthing parent at index, years						
15–22	2.9	2.8	2.8	2.8	2.8	2.8
23–29	35.3	35.6	35.6	35.6	36.2	35.6
30–34	39.7	39.4	39.5	39.4	39.1	39.4
35–39	18.8	18.9	18.7	18.8	18.5	18.9
40–49	3.3	3.4	3.3	3.4	3.5	3.4
Region of residence						
Northeast	14.7	14.6	14.6	14.6	14.7	14.6
Midwest	24.2	24.3	24.2	24.3	24.0	24.2
South	43.3	43.4	43.6	43.4	43.8	43.4
West	16.9	16.9	16.6	16.9	16.6	16.9
Unknown	0.9	0.9	0.9	0.9	0.9	0.9
Urbanicity						
Urban	83.7	83.7	83.4	83.7	84.0	83.7
Rural	12.2	12.2	12.3	12.2	12.0	12.1
Missing	4.1	4.1	4.2	4.1	4.1	4.1
Year of index antibiotic fill						
2006–2010	32.5	32.7	32.5	32.7	32.6	32.7
2011–2016	40.8	40.9	41.3	41.0	41.6	40.9
2017–2022	26.8	26.4	26.2	26.4	25.8	26.4
Comorbidities[a]						
Pre-gestational diabetes or insulin	1.4	1.4	1.4	1.4	1.4	1.4
Chronic heart disease	0.0	0.0	0.0	0.0	0.0	0.0
Chronic hypertension	3.1	3.1	3.1	3.1	3.0	3.1
Obesity	4.3	4.3	4.2	4.3	4.2	4.3

© 2025 Osmundson SS et al. *JAMA Network Open.*

Characteristic	Nitrofurantoin vs β-lactam		TMP-SMX vs β-lactam		Fluoroquinolone vs β-lactam	
	Weighted Nitrofurantoin (%)	Weighted β-lactam (%)	Weighted TMP-SMX (%)	Weighted β-lactam (%)	Weighted Fluoroquinolone (%)	Weighted β-lactam (%)
Pelvic inflammatory disease or sexually transmitted infection	7.5	7.6	7.8	7.6	7.9	7.6
Alcohol use disorder	0.1	0.1	0.2	0.1	0.1	0.1
Other substance use disorder	0.2	0.2	0.2	0.2	0.2	0.2
Tobacco use	1.9	1.9	2.0	1.9	1.9	1.9
Suspected teratogenic medication[b]						
Benzodiazepine	3.1	3.0	3.1	3.0	3.0	3.0
Fluconazole[c]	1.0	1.0	1.1	1.0	1.1	1.0
Folic acid antagonist	0.7	0.7	0.7	0.7	0.7	0.7
Selective serotonin reuptake inhibitor	8.2	8.2	8.2	8.2	7.7	8.2
Other suspected teratogen[d]	1.1	1.1	1.2	1.1	1.2	1.1
Other medications						
Non-index antibiotic[e]	12.7	12.6	12.7	12.6	13.1	12.6
Oral antidiabetic[a]	3.4	3.4	3.5	3.4	3.4	3.4
Pre-pregnancy health care utilization intensity[f]						
Any hospitalization	0.8	0.8	0.8	0.8	0.8	0.8
Any emergency department visit	5.8	5.8	5.7	5.8	5.8	5.8
No. of outpatient office visits, mean (SD)	1.3 (2.4)	1.3 (1.7)	1.3 (7.5)	1.3 (1.7)	1.3 (6.6)	1.3 (1.7)
No. of prescription therapeutic groups, mean (SD)	3.9 (3.4)	3.9 (2.4)	3.9 (10.0)	3.9 (2.4)	3.9 (9.4)	3.9 (2.4)

Abbreviations: SD, standard deviation; TMP-SMX, trimethoprim-sulfamethoxazole; UTI, urinary tract infection.

[a] Identified during the baseline period. For diagnosis codes, this was defined as last menstrual period date -180 through index antibiotic date. Baseline period for medications was defined as last menstrual period date -90 through index antibiotic date.

[b] Identified as days' supply overlapping the first trimester.

[c] 12,000 mg of fluconazole exposure during the exposure period was required based on prescription days' supply, dose, and quantity.

[d] Other suspected teratogenic medications were as follows: aminoglycoside, angiotensin-converting enzyme inhibitor, angiotensin receptor blocker, danazol, methimazole, molnupiravir, mycophenolate, potassium iodide, propylthiouracil, ribavirin, statin, tetracycline.

© 2025 Osmundson SS et al. *JAMA Network Open*.

[e] Identified during the first trimester.

[f] Hospitalizations, ED visits, and outpatient office visits identified pre-pregnancy (LMP-90 days through LMP date). Prescriptions identified pre-pregnancy and during pregnancy defined as LMP date-90 days through pregnancy outcome date; the index antibiotic prescription did not count.

[g] Identified ± 7 days from the index antibiotic date.

© 2025 Osmundson SS et al. *JAMA Network Open*.

eFigure 1. Derivation of Cohort of Pregnant Individuals Treated for Urinary Tract Infection (UTI) in the First Trimester with an Antibiotic Agent in the MarketScan Commercial Database

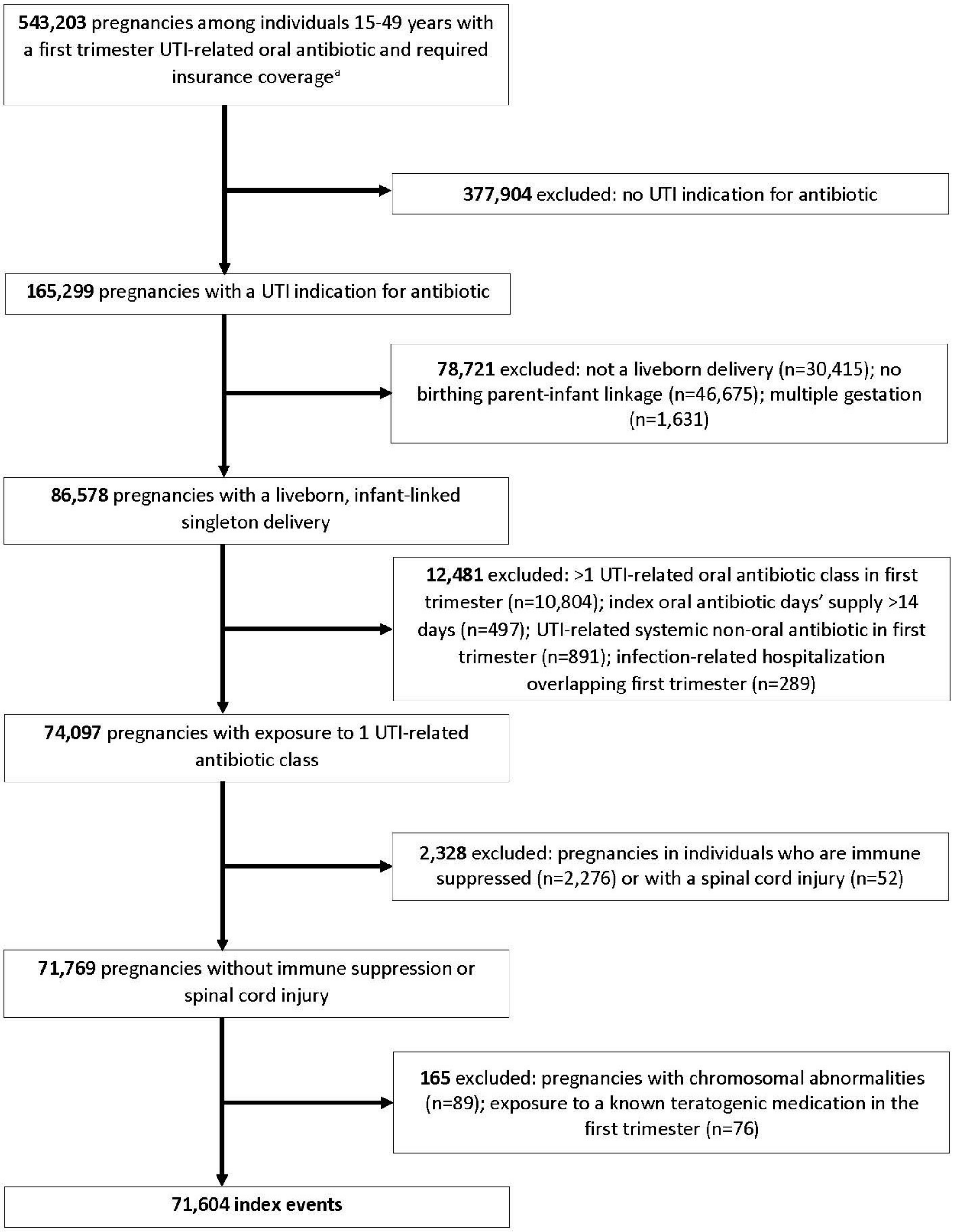

[a] Required insurance coverage: last menstrual period date -90 days through end of pregnancy +30 days

© 2025 Osmundson SS et al. *JAMA Network Open*.

eFigure 2. Utilization of Antibiotic Agents for the Treatment of Urinary Tract Infection During the First Trimester of Pregnancy by Calendar Year

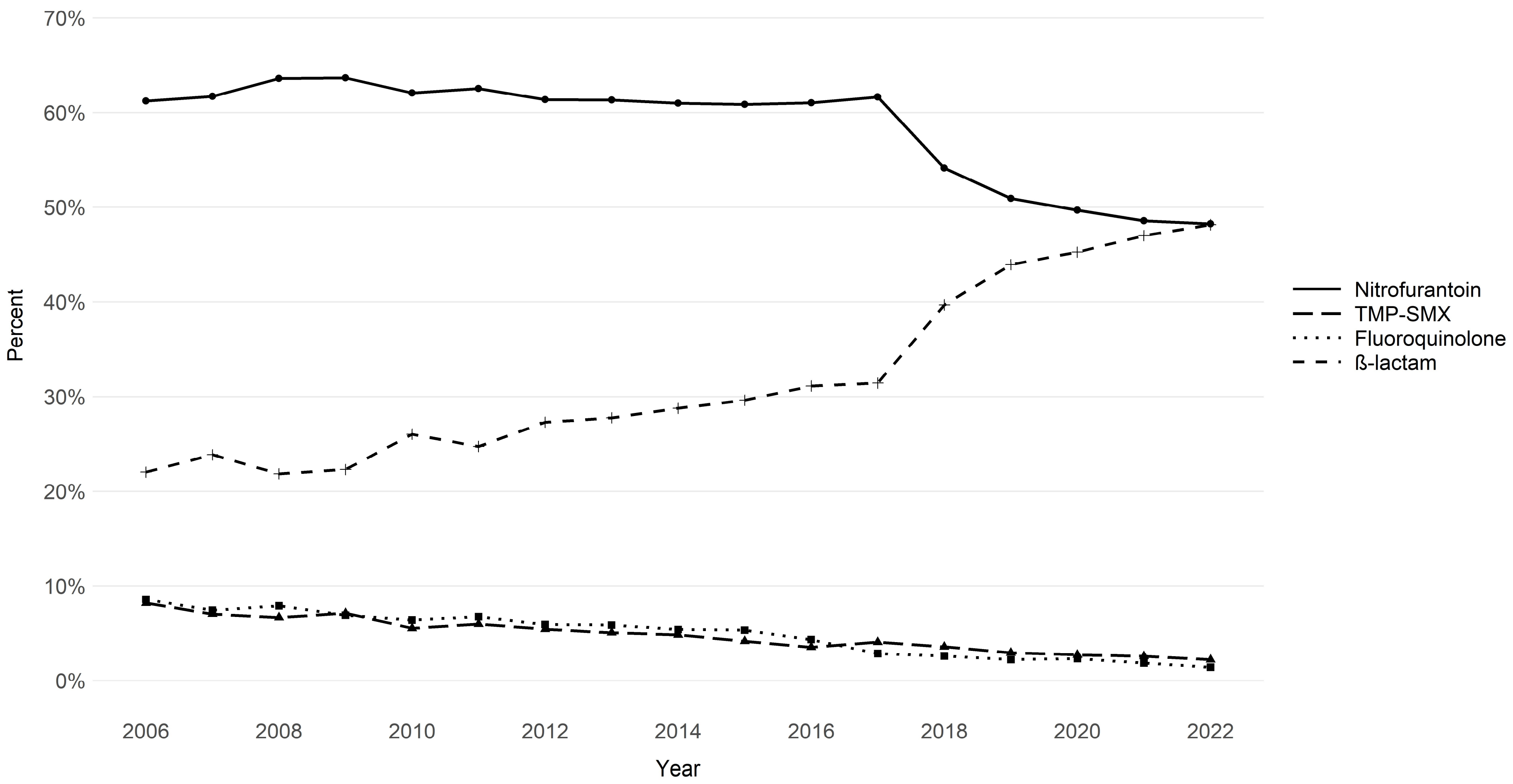

Abbreviations: TMP-SMX, trimethoprim-sulfamethoxazole.

© 2025 Osmundson SS et al. *JAMA Network Open*.

eFigure 3. Standardized Mean Differences of Patient Characteristics Among Pregnant Individuals Treated for Urinary Tract Infection in the First Trimester Between Each Antibiotic Exposure Group versus β-lactam Users (Reference Group), in the Unweighted and Weighted Populations, Primary Analysis[a, b, c]

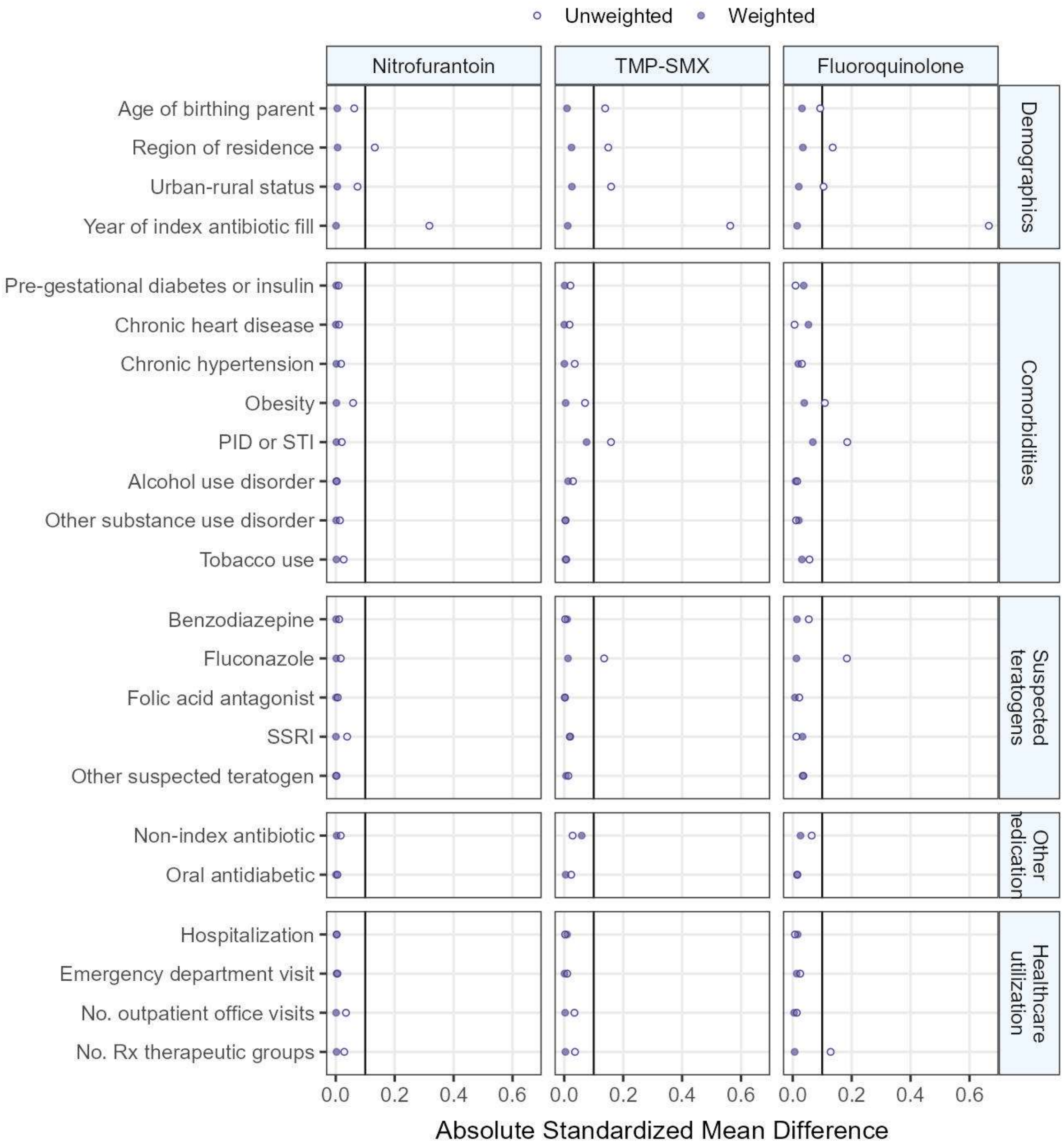

Abbreviations: PID, pelvic inflammatory disease; SSRI, selective serotonin reuptake inhibitor; STI, sexually transmitted infection; TMP-SMX, trimethoprim/sulfamethoxazole.

[a] Unweighted population (N=71,604); weighted population for each treatment comparison reported in eTable 12 (sum of weights).
[b] Standardized mean difference (SMD) calculated as the difference in means or proportions of each covariate (listed on far left of the plot) divided by the pooled standard deviation of the covariate. All SMD estimates compare individuals who received a β-lactam (reference) versus a comparator antibiotic treatment. SMD estimates are reported in the: a) unweighted population (outlined circle) and b) weighted population after cohort-specific trimming (solid circle). SMDs <0.10 (denoted by the black vertical line in each plot) indicate balance, i.e., no substantial difference in means or proportions between groups. In the standardized mortality ratio-weighted population, all measured baseline characteristics were well-balanced between exposure groups for all analyses including sensitivity analyses (i.e., all SMD <0.10).

© 2025 Osmundson SS et al. *JAMA Network Open*.

[c] SMD <0.10 for all primary analyses after weighting. SMD <0.10 for all sensitivity analyses with exceptions in the following UTI cohort analyses: exposure between 4-9 weeks, fluoroquinolone vs β-lactam: baseline PID or STI (0.1038); symptomatic UTI, fluoroquinolone vs β-lactam: baseline PID or STI (0.1095); symptomatic UTI, TMP-SMX vs β-lactam: baseline PID or STI (0.1150); adjusted for gestational age at exposure, fluoroquinolone vs β-lactam: region (0.1742), baseline oral antidiabetics (0.1586), baseline obesity (0.1294), and baseline diabetes diagnosis or insulin (0.1140).

© 2025 Osmundson SS et al. *JAMA Network Open*.

eFigure 4. Weighted Risk Difference Estimates of Any Congenital Malformation in Infants Born to Individuals Treated for Urinary Tract Infection (UTI) in the First Trimester: Comparison of Different UTI-related Antibiotics Versus β-lactams (Reference)[a,b]

	No. of events (comparator)	Total no. of infants (comparator)	No. of events (reference)	Total no. of infants (reference)	Weighted RD per 1000 (95% CI)
**Primary analysis**					
Nitrofurantoin	901	42,398	437	22,042	2.41 (0.00, 4.82)
TMP-SMX	94	3494	436	22,032	6.88 (0.11, 13.65)
Fluoroquinolone	86	3662	437	22,014	3.63 (-3.43, 10.70)
**Sensitivity analysis**					
Alternative reference group					
Nitrofurantoin	901	42,394	281	15,164	3.65 (0.98, 6.31)
TMP-SMX	94	3494	278	15,143	9.02 (1.74, 16.30)
Fluoroquinolone	86	3659	281	15,142	4.80 (-2.52, 12.13)
Alternative outcome definition					
Nitrofurantoin	1363	42,398	650	22,042	3.04 (0.14, 5.93)
TMP-SMX	132	3494	649	22,032	5.30 (-2.14, 12.73)
Fluoroquinolone	119	3662	650	22,014	0.89 (-6.83, 8.62)
Restricted to symptomatic UTI					
Nitrofurantoin	372	17,980	184	9087	1.42 (-2.38, 5.21)
TMP-SMX	65	2362	182	9054	7.66 (-1.40, 16.71)
Fluoroquinolone	74	3108	183	9036	4.27 (-4.57, 13.11)
Restricted to ICD-10 era					
Nitrofurantoin	389	16,081	219	11,285	5.35 (1.72, 8.97)
TMP-SMX	30	978	210	11,037	10.27 (-0.90, 21.44)
Fluoroquinolone	21	901	219	11,166	1.85 (-9.10, 12.80)
Alternative exposure window 2–13 weeks					
Nitrofurantoin	862	40,443	424	21,466	2.55 (0.09, 5.01)
TMP-SMX	65	2578	423	21,458	4.20 (-2.86, 11.27)
Fluoroquinolone	55	2287	424	21,430	2.94 (-5.15, 11.02)
Adjusted for gestational age at exposure					
Nitrofurantoin	901	42,398	437	22,042	2.34 (-0.07, 4.75)
TMP-SMX	94	3494	435	22,003	6.08 (-1.46, 13.62)
Fluoroquinolone	85	3659	435	21,985	14.43 (-8.96, 37.81)

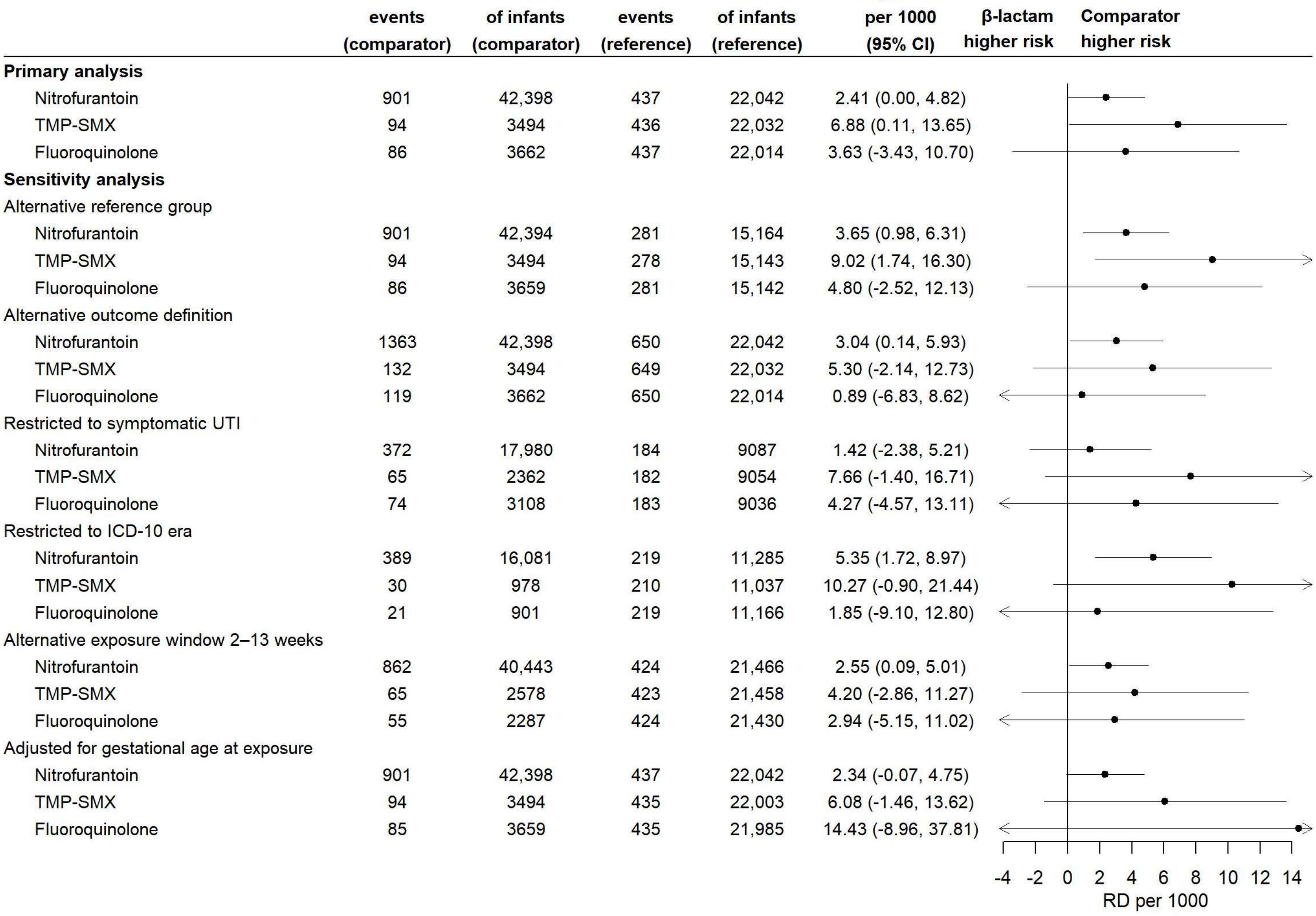

© 2025 Osmundson SS et al. *JAMA Network Open*.

Abbreviations: CI, confidence interval; ICD-10-CM, International Classification of Diseases, Tenth Revision, Clinical Modification; RD, risk difference; TMP-SMX, trimethoprim-sulfamethoxazole; UTI, urinary tract infection.

[a] Sensitivity analysis alternative reference group subsetted to amoxicillin alone or cephalexin (rather than all UTI-related β-lactam antibiotics). Sensitivity analysis alternative outcome definition relaxed the congenital malformation algorithm to allow any single diagnosis code within the specific malformation group (after excluding diagnostic/rule-out codes). Sensitivity analysis symptomatic UTI restricted to symptomatic UTIs (rather than including asymptomatic bacteriuria); symptomatic UTI defined as meeting ≥1 of the following criteria: urine culture on the same date as the antibiotic; no urine culture ± 7 days of the antibiotic; a UTI symptom diagnosis code ± 7 days of the antibiotic. Sensitivity analysis ICD-10 era restricted to pregnancies with delivery dates on or after October 1, 2015. Sensitivity analysis alternative exposure window restricted the antibiotic exposure timeframe from 0-13 weeks to 2-13 weeks gestation (14-97 days of gestation). Sensitivity analysis adjusted for gestational age at exposure using gestational age distribution tertiles (0-6 weeks, 7-9 weeks, 10-13 weeks).

[b] Propensity score weighted analysis accounted for all potential confounders listed in eTable 8. We required ≥5 events in each exposure group to estimate the treatment effect.

© 2025 Osmundson SS et al. *JAMA Network Open*.

eFigure 5. Weighted Risk Difference Estimates of Any Cardiac Malformation in Infants Born to Individuals Treated for Urinary Tract Infection (UTI) in the First Trimester: Comparison of Different UTI-related Antibiotics Versus β-lactams (Reference)[a,b]

	No. of events (comparator)	Total no. of infants (comparator)	No. of events (reference)	Total no. of infants (reference)	Weighted RD per 1000 (95% CI)
**Primary analysis**					
Nitrofurantoin	437	42,398	207	22,042	1.44 (-0.24, 3.11)
TMP-SMX	47	3494	207	22,032	4.20 (-0.71, 9.11)
Fluoroquinolone	38	3662	207	22,014	2.15 (-3.15, 7.45)
**Sensitivity analysis**					
Alternative reference group					
Nitrofurantoin	437	42,394	137	15,164	1.74 (-0.13, 3.61)
TMP-SMX	47	3494	135	15,143	4.97 (-0.35, 10.30)
Fluoroquinolone	38	3659	137	15,142	2.43 (-3.07, 7.94)
Alternative outcome definition					
Nitrofurantoin	641	42,398	301	22,042	1.68 (-0.32, 3.67)
TMP-SMX	58	3494	301	22,032	2.19 (-3.01, 7.38)
Fluoroquinolone	49	3662	301	22,014	-0.07 (-5.59, 5.46)
Restricted to symptomatic UTI					
Nitrofurantoin	169	17,980	85	9087	0.40 (-2.17, 2.97)
TMP-SMX	28	2362	84	9054	1.51 (-3.83, 6.86)
Fluoroquinolone	36	3108	85	9036	3.58 (-3.05, 10.21)
Restricted to ICD-10 era					
Nitrofurantoin	182	16,081	115	11,285	1.84 (-0.77, 4.44)
TMP-SMX	17	978	108	11,037	6.48 (-1.83, 14.78)
Fluoroquinolone	11	901	115	11,166	2.59 (-6.18, 11.35)
Alternative exposure window 4–9 weeks					
Nitrofurantoin	222	21,677	126	12,152	0.30 (-2.10, 2.69)
TMP-SMX	12	1036	126	12,106	-0.83 (-7.33, 5.67)
Fluoroquinolone	9	745	123	12,036	7.04 (-6.46, 20.55)
Adjusted for gestational age at exposure					
Nitrofurantoin	437	42,398	207	22,042	1.43 (-0.24, 3.11)
TMP-SMX	47	3494	206	22,003	6.15 (-0.13, 12.44)
Fluoroquinolone	38	3659	207	21,985	8.80 (-9.65, 27.26)

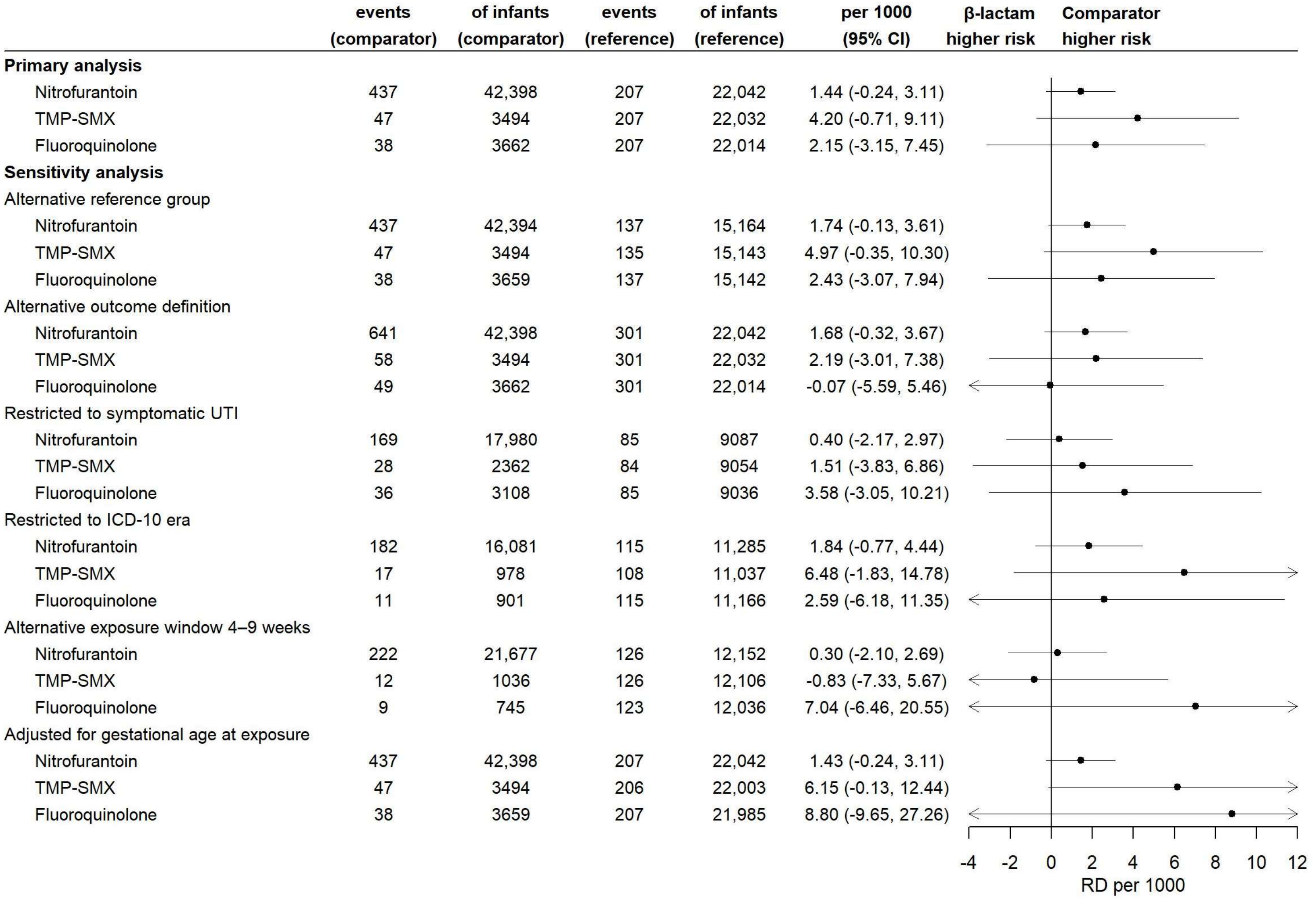

© 2025 Osmundson SS et al. *JAMA Network Open*.

Abbreviations: CI, confidence interval; ICD-10-CM, International Classification of Diseases, Tenth Revision, Clinical Modification; RD, risk difference; TMP-SMX, trimethoprim-sulfamethoxazole; UTI, urinary tract infection.

[a] Sensitivity analysis alternative reference group subsetted to amoxicillin alone or cephalexin (rather than all UTI-related β-lactam antibiotics). Sensitivity analysis alternative outcome definition relaxed the congenital malformation algorithm to allow any single diagnosis code within the specific malformation group (after excluding diagnostic/rule-out codes). Sensitivity analysis symptomatic UTI restricted to symptomatic UTIs (rather than including asymptomatic bacteriuria); symptomatic UTI defined as meeting ≥1 of the following criteria: urine culture on the same date as the antibiotic; no urine culture ± 7 days of the antibiotic; a UTI symptom diagnosis code ± 7 days of the antibiotic. Sensitivity analysis ICD-10 era restricted to pregnancies with delivery dates on or after October 1, 2015. Sensitivity analysis alternative exposure window restricted the antibiotic exposure timeframe from 0-13 weeks to 4-9 weeks gestation (28-69 days of gestation). Sensitivity analysis adjusted for gestational age at exposure using gestational age distribution tertiles (0-6 weeks, 7-9 weeks, 10-13 weeks).

[b] Propensity score weighted analysis accounted for all potential confounders listed in eTable 8. We required ≥5 events in each exposure group to estimate the treatment effect.

© 2025 Osmundson SS et al. *JAMA Network Open*.

eFigure 6. Weighted Risk Ratio Estimates of Other Organ-Specific Malformations in Infants Born to Individuals Treated for Urinary Tract Infection (UTI) in the First Trimester: Comparison of Different UTI-related Antibiotics Versus β-lactams (Reference)[a]

	No. of events (comparator)	Total no. of infants (comparator)	No. of events (reference)	Total no. of infants (reference)	Weighted RR (95% CI)
**Central nervous system**					
Nitrofurantoin	28	42,398	16	22,042	0.93 (0.49, 1.80)
TMP-SMX	1	3494	16	22,032	NE
Fluoroquinolone	9	3662	16	22,014	2.39 (0.84, 6.80)
**Orofacial/Respiratory malformations**					
Nitrofurantoin	57	42,398	29	22,042	1.12 (0.70, 1.78)
TMP-SMX	11	3494	29	22,032	2.89 (1.31, 6.41)
Fluoroquinolone	6	3662	29	22,014	1.68 (0.52, 5.43)
**Gastrointestinal malformations**					
Nitrofurantoin	120	42,398	59	22,042	1.01 (0.73, 1.39)
TMP-SMX	11	3494	59	22,032	0.72 (0.35, 1.48)
Fluoroquinolone	12	3662	59	22,014	1.66 (0.78, 3.52)
**Genitourinary/Renal malformations**					
Nitrofurantoin	202	42,398	108	22,042	1.09 (0.86, 1.38)
TMP-SMX	17	3494	108	22,032	1.13 (0.63, 2.03)
Fluoroquinolone	14	3662	108	22,014	0.65 (0.29, 1.44)
**Musculoskeletal malformations**					
Nitrofurantoin	71	42,398	30	22,042	1.11 (0.72, 1.72)
TMP-SMX	6	3494	29	22,032	0.71 (0.27, 1.87)
Fluoroquinolone	10	3662	30	22,014	2.04 (0.77, 5.41)

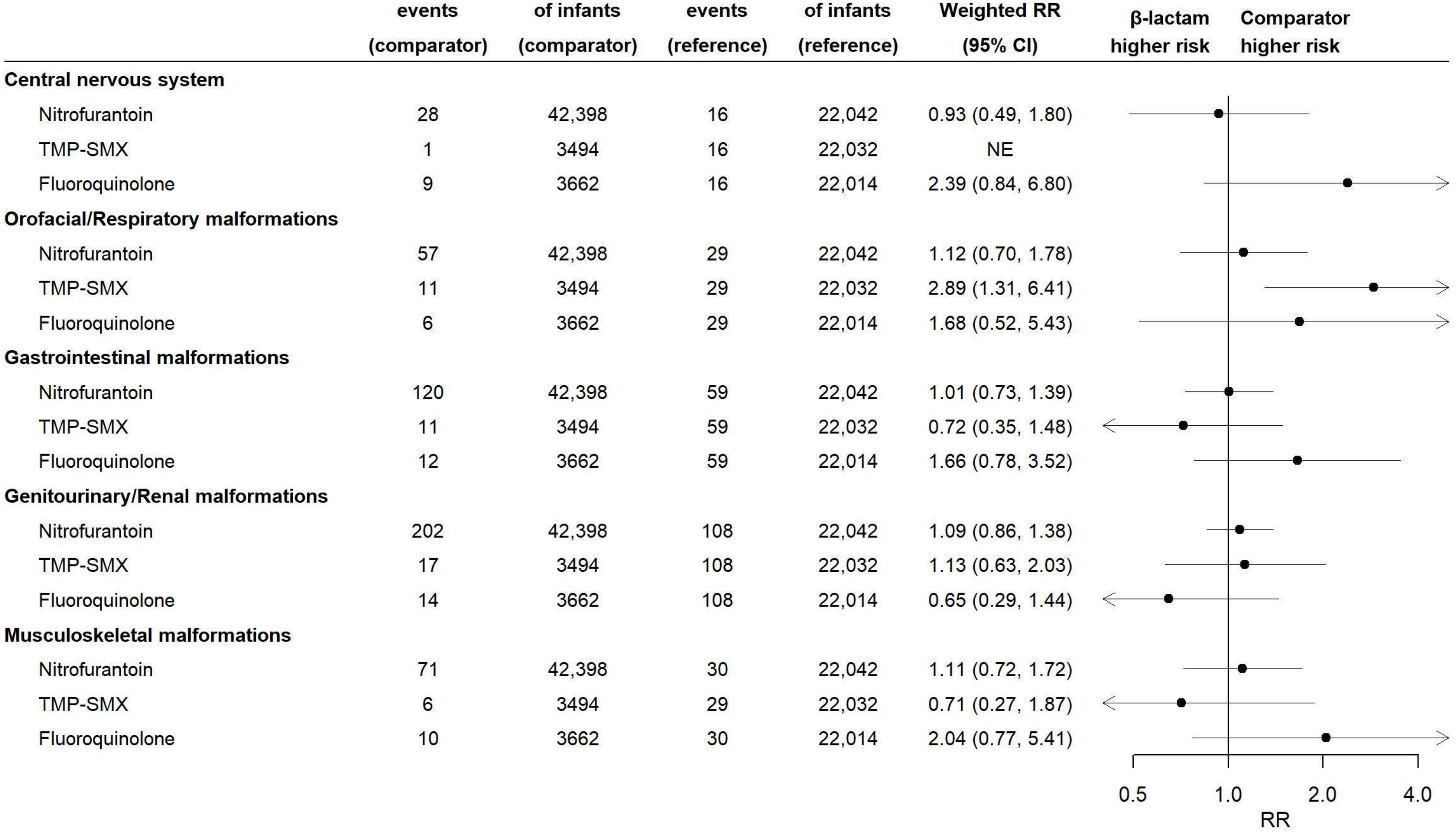

Abbreviations: CI, confidence interval; NE, not estimable; RR, risk ratio; TMP-SMX, trimethoprim-sulfamethoxazole.

© 2025 Osmundson SS et al. *JAMA Network Open*.

[a] Propensity score weighted analysis accounted for all potential confounders listed in eTable 8. We required ≥50 events in the UTI cohort to perform organ-specific malformation analyses. We required ≥5 events in each exposure group to estimate the treatment effect.

© 2025 Osmundson SS et al. *JAMA Network Open*.

eFigure 7. Weighted Risk Difference Estimates of Other Organ-Specific Malformations in Infants Born to Individuals Treated for Urinary Tract Infection (UTI) in the First Trimester: Comparison of Different UTI-related Antibiotics Versus β-lactams (Reference)[a]

	No. of events (comparator)	Total no. of infants (comparator)	No. of events (reference)	Total no. of infants (reference)	Weighted RD per 1000 (95% CI)
**Central nervous system**					
Nitrofurantoin	28	42,398	16	22,042	-0.05 (-0.51, 0.42)
TMP-SMX	1	3494	16	22,032	NE
Fluoroquinolone	9	3662	16	22,014	1.01 (-0.61, 2.63)
**Orofacial/Respiratory malformations**					
Nitrofurantoin	57	42,398	29	22,042	0.16 (-0.48, 0.79)
TMP-SMX	11	3494	29	22,032	2.49 (-0.24, 5.23)
Fluoroquinolone	6	3662	29	22,014	0.90 (-1.62, 3.42)
**Gastrointestinal malformations**					
Nitrofurantoin	120	42,398	59	22,042	0.02 (-0.84, 0.87)
TMP-SMX	11	3494	59	22,032	-0.74 (-2.21, 0.72)
Fluoroquinolone	12	3662	59	22,014	1.76 (-1.46, 4.98)
**Genitourinary/Renal malformations**					
Nitrofurantoin	202	42,398	108	22,042	0.43 (-0.78, 1.65)
TMP-SMX	17	3494	108	22,032	0.64 (-2.56, 3.85)
Fluoroquinolone	14	3662	108	22,014	-1.72 (-4.36, 0.92)
**Musculoskeletal malformations**					
Nitrofurantoin	71	42,398	30	22,042	0.15 (-0.46, 0.76)
TMP-SMX	6	3494	29	22,032	-0.38 (-1.35, 0.58)
Fluoroquinolone	10	3662	30	22,014	1.42 (-1.15, 3.99)

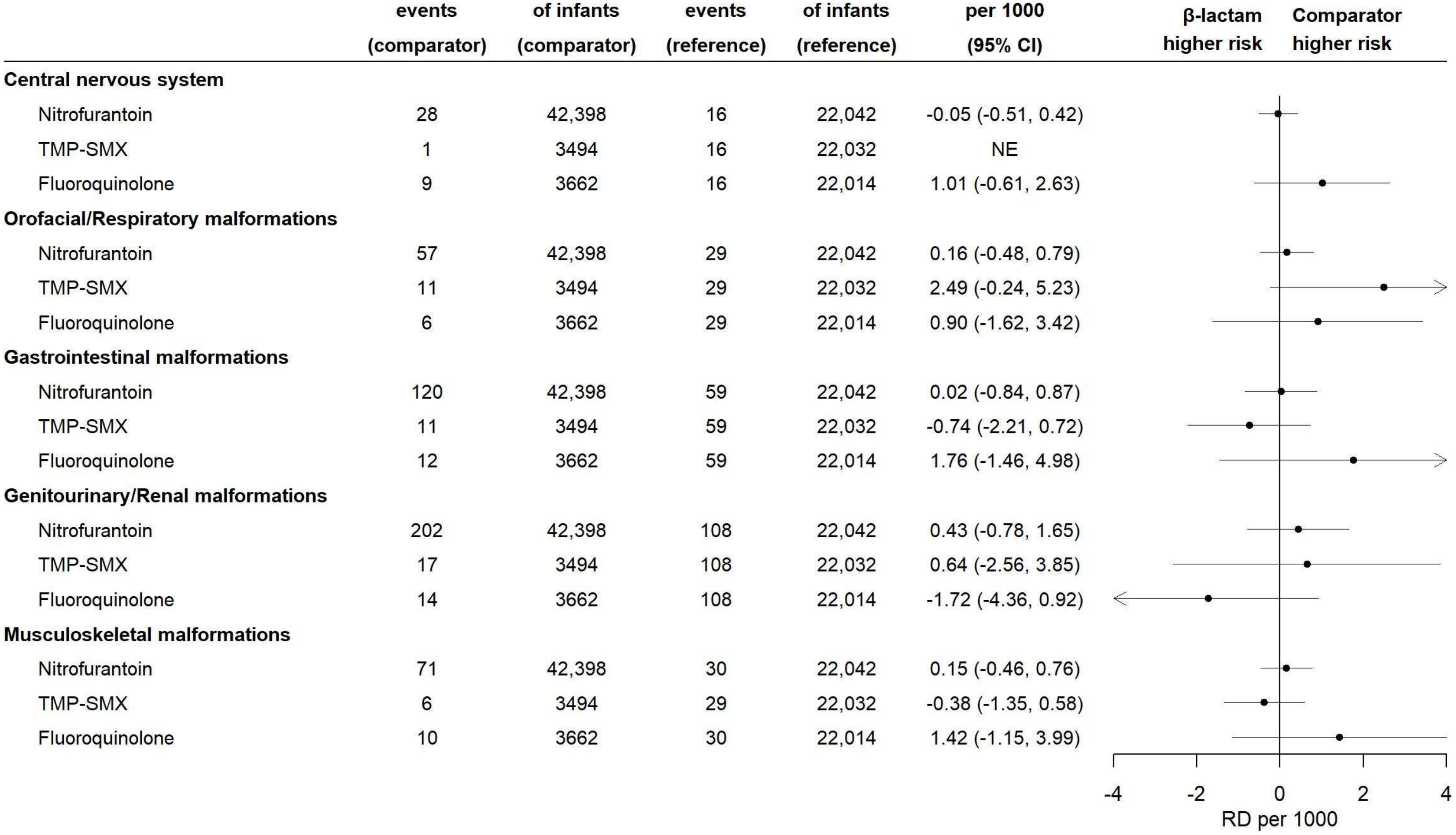

Abbreviations: CI, confidence interval; NE, not estimable; RD, risk difference; TMP-SMX, trimethoprim-sulfamethoxazole.

© 2025 Osmundson SS et al. *JAMA Network Open*.

[a] Propensity score weighted analysis accounted for all potential confounders listed in eTable 8. We required ≥50 events in the UTI cohort to perform organ-specific malformation analyses. We required ≥5 events in each exposure group to estimate the treatment effect.

© 2025 Osmundson SS et al. *JAMA Network Open*.

eFigure 8. Weighted Risk Difference Estimates of Specific Malformation Groups in Infants Born to Individuals Treated for Urinary Tract Infection (UTI) in the First Trimester: Comparison of Different UTI-related Antibiotics Versus β-lactams (Reference)[a]

	No. of events (comparator)	Total no. of infants (comparator)	No. of events (reference)	Total no. of infants (reference)	Weighted RD per 1000 (95% CI)
**Severe cardiac defects**					
Nitrofurantoin	117	42,398	53	22,042	0.49 (-0.37, 1.35)
TMP-SMX	16	3494	53	22,032	2.62 (-0.40, 5.64)
Fluoroquinolone	4	3662	53	22,014	NE
**Other cardiac defects**					
Nitrofurantoin	392	42,398	184	22,042	1.22 (-0.36, 2.80)
TMP-SMX	43	3494	184	22,032	4.32 (-0.48, 9.11)
Fluoroquinolone	36	3662	184	22,014	2.38 (-2.76, 7.51)
**Neural tube defects**					
Nitrofurantoin	18	42,398	16	22,042	-0.26 (-0.70, 0.18)
TMP-SMX	1	3494	16	22,032	NE
Fluoroquinolone	7	3662	16	22,014	0.64 (-0.89, 2.17)
**Cleft lip and/or cleft palate**					
Nitrofurantoin	52	42,398	26	22,042	0.14 (-0.46, 0.74)
TMP-SMX	11	3494	26	22,032	2.63 (-0.10, 5.36)
Fluoroquinolone	6	3662	26	22,014	1.04 (-1.48, 3.55)
**Intestinal atresia or stenosis**					
Nitrofurantoin	47	42,398	23	22,042	-0.04 (-0.57, 0.49)
TMP-SMX	3	3494	23	22,032	NE
Fluoroquinolone	6	3662	23	22,014	0.54 (-0.83, 1.91)
**Pyloric stenosis**					
Nitrofurantoin	65	42,398	29	22,042	0.16 (-0.45, 0.77)
TMP-SMX	7	3494	29	22,032	-0.07 (-1.24, 1.10)
Fluoroquinolone	6	3662	29	22,014	1.54 (-1.37, 4.45)
**Hypospadias (male infants only)**					
Nitrofurantoin	175	42,398	84	22,042	1.50 (-0.65, 3.65)
TMP-SMX	14	3494	84	22,032	1.05 (-4.50, 6.60)
Fluoroquinolone	11	3662	84	22,014	-2.29 (-7.22, 2.65)
**Limb deficiency**					
Nitrofurantoin	41	42,398	20	22,042	-0.09 (-0.56, 0.39)
TMP-SMX	6	3494	19	22,032	0.07 (-0.85, 0.99)
Fluoroquinolone	6	3662	20	22,014	0.08 (-0.82, 0.97)

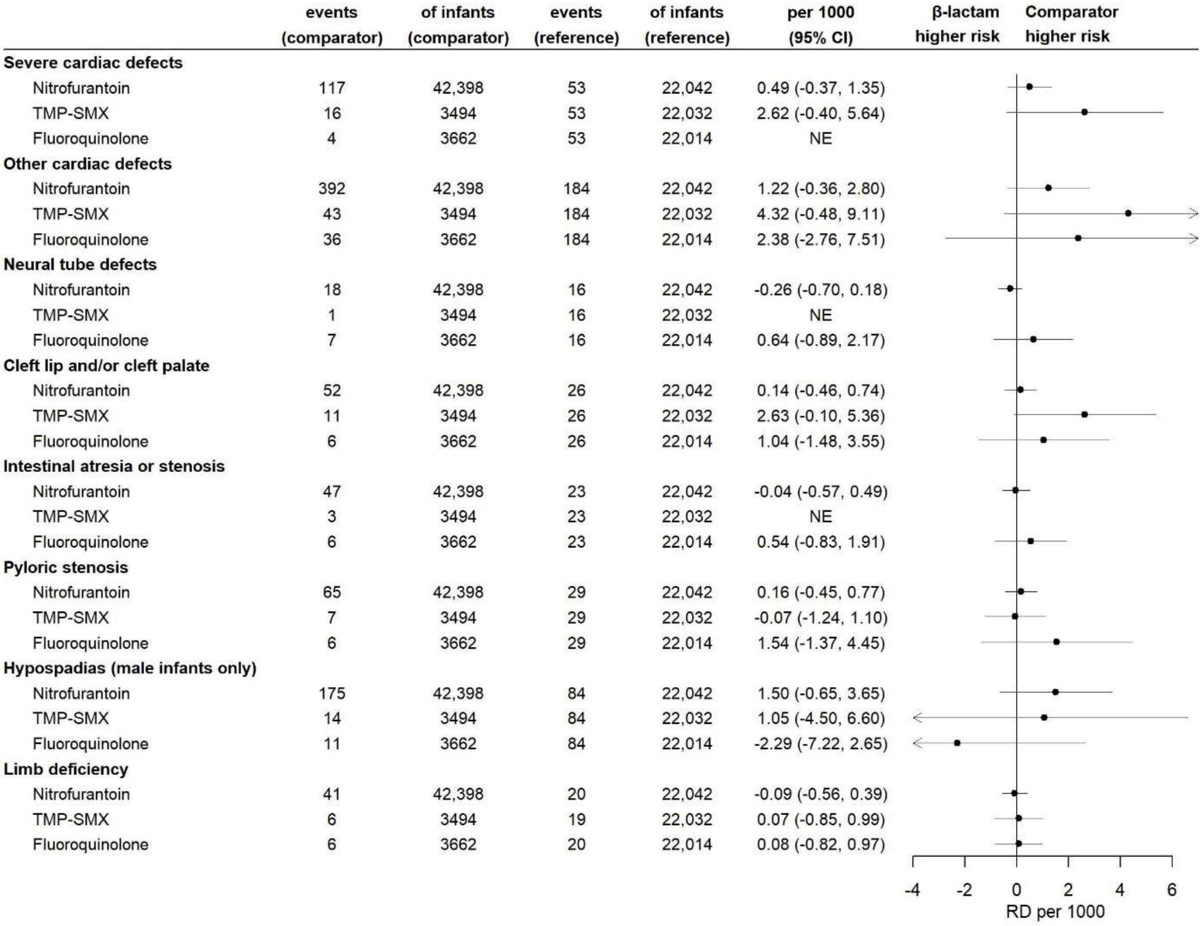

Abbreviations: CI, confidence interval; NE, not estimable; RD, risk difference; TMP-SMX, trimethoprim-sulfamethoxazole.

[a] Propensity score weighted analysis accounted for all potential confounders listed in eTable 8. We required ≥50 events in the UTI cohort to perform specific malformation group analyses. We required ≥5 events in each exposure group to estimate the treatment effect.

© 2025 Osmundson SS et al. *JAMA Network Open*.

eFigure 9. Weighted Risk Ratio Estimates of Severe Cardiac Defect, Other Cardiac Defect, and Cleft Lip and/or Cleft Palate in Infants Born to Individuals Treated for Urinary Tract Infection (UTI) in the First Trimester: Comparison of Different UTI-related Antibiotics Versus β-lactams (Reference)[a]

	No. of events (comparator)	Total no. of infants (comparator)	No. of events (reference)	Total no. of infants (reference)	Weighted RR (95% CI)
**Severe cardiac defects**					
Primary analysis					
Nitrofurantoin	117	42,398	53	22,042	1.20 (0.86, 1.68)
TMP-SMX	16	3494	53	22,032	2.09 (1.09, 3.99)
Fluoroquinolone	4	3662	53	22,014	NE
Alternative exposure window 4–9 weeks					
Nitrofurantoin	54	21,677	33	12,152	0.94 (0.60, 1.48)
TMP-SMX	5	1036	33	12,106	1.38 (0.51, 3.69)
Fluoroquinolone	1	745	32	12,036	NE
**Other cardiac defects**					
Primary analysis					
Nitrofurantoin	392	42,398	184	22,042	1.15 (0.96, 1.37)
TMP-SMX	43	3494	184	22,032	1.52 (1.02, 2.25)
Fluoroquinolone	36	3662	184	22,014	1.28 (0.79, 2.09)
Alternative exposure window 4–9 weeks					
Nitrofurantoin	208	21,677	112	12,152	1.07 (0.84, 1.36)
TMP-SMX	10	1036	112	12,106	0.83 (0.39, 1.76)
Fluoroquinolone	9	745	110	12,036	1.89 (0.85, 4.19)
**Cleft lip and/or cleft palate**					
Primary analysis					
Nitrofurantoin	52	42,398	26	22,042	1.12 (0.69, 1.82)
TMP-SMX	11	3494	26	22,032	3.23 (1.44, 7.22)
Fluoroquinolone	6	3662	26	22,014	1.88 (0.58, 6.10)
Alternative exposure window 7–10 weeks					
Nitrofurantoin	15	19,284	12	10,627	0.66 (0.30, 1.46)
TMP-SMX	0	636	10	10,191	NE
Fluoroquinolone	1	213	10	10,137	NE

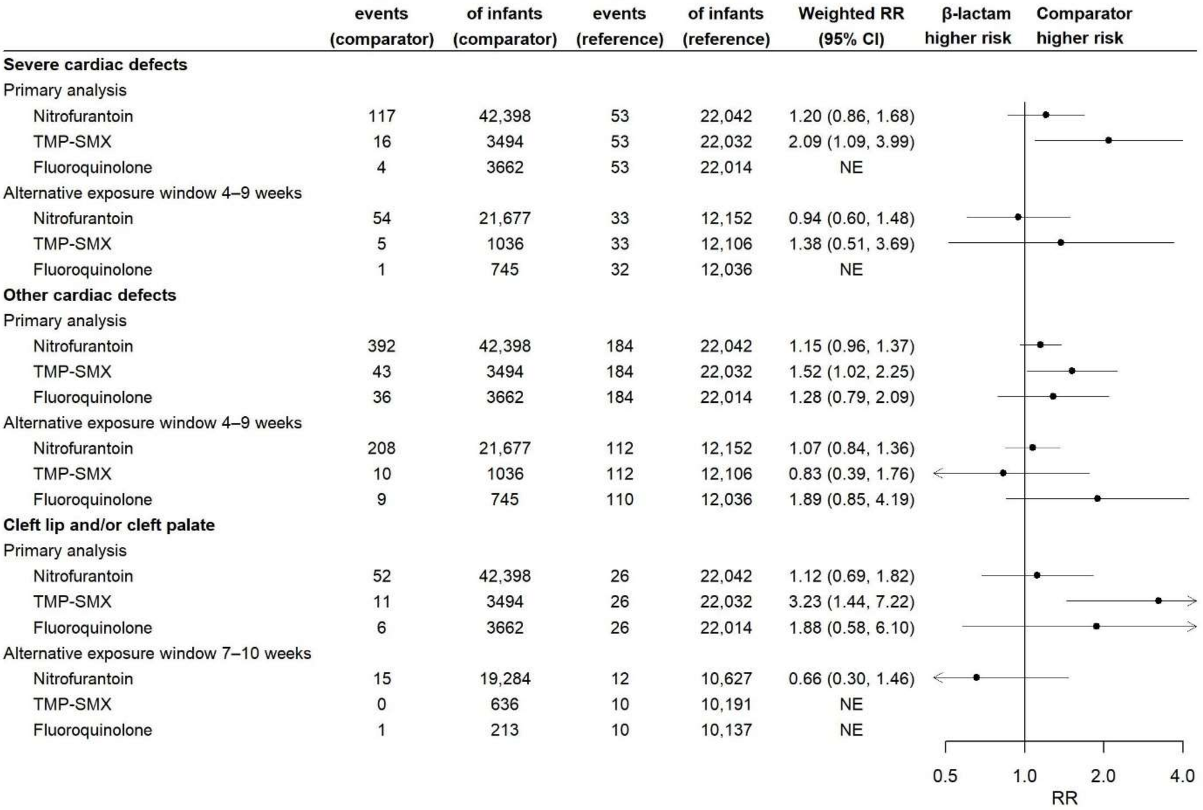

Abbreviations: CI, confidence interval; NE, not estimable; RR, risk ratio; TMP-SMX, trimethoprim-sulfamethoxazole.

[a] Propensity score weighted analysis accounted for all potential confounders listed in eTable 8. We required ≥50 events in the UTI cohort to perform specific malformation group analyses. We required ≥5 events in each exposure group to estimate the treatment effect.

© 2025 Osmundson SS et al. *JAMA Network Open*.

eFigure 10. Weighted Risk Difference Estimates of Severe Cardiac Defect, Other Cardiac Defect, and Cleft Lip and/or Cleft Palate in Infants Born to Individuals Treated for Urinary Tract Infection (UTI) in the First Trimester: Comparison of Different UTI-related Antibiotics Versus β-lactams (Reference)[a]

	No. of events (comparator)	Total no. of infants (comparator)	No. of events (reference)	Total no. of infants (reference)	Weighted RD per 1000 (95% CI)
**Severe cardiac defects**					
Primary analysis					
Nitrofurantoin	117	42,398	53	22,042	0.49 (-0.37, 1.35)
TMP-SMX	16	3494	53	22,032	2.62 (-0.40, 5.64)
Fluoroquinolone	4	3662	53	22,014	NE
Alternative exposure window 4–9 weeks					
Nitrofurantoin	54	21,677	33	12,152	-0.15 (-1.35, 1.05)
TMP-SMX	5	1036	33	12,106	1.02 (-2.57, 4.62)
Fluoroquinolone	1	745	32	12,036	NE
**Other cardiac defects**					
Primary analysis					
Nitrofurantoin	392	42,398	184	22,042	1.22 (-0.36, 2.80)
TMP-SMX	43	3494	184	22,032	4.32 (-0.48, 9.11)
Fluoroquinolone	36	3662	184	22,014	2.38 (-2.76, 7.51)
Alternative exposure window 4–9 weeks					
Nitrofurantoin	208	21,677	112	12,152	0.64 (-1.63, 2.92)
TMP-SMX	10	1036	112	12,106	-1.59 (-7.44, 4.26)
Fluoroquinolone	9	745	110	12,036	8.12 (-5.37, 21.62)
**Cleft lip and/or cleft palate**					
Primary analysis					
Nitrofurantoin	52	42,398	26	22,042	0.14 (-0.46, 0.74)
TMP-SMX	11	3494	26	22,032	2.63 (-0.10, 5.36)
Fluoroquinolone	6	3662	26	22,014	1.04 (-1.48, 3.55)
Alternative exposure window 7–10 weeks					
Nitrofurantoin	15	19,284	12	10,627	-0.39 (-1.15, 0.38)
TMP-SMX	0	636	10	10,191	NE
Fluoroquinolone	1	213	10	10,137	NE

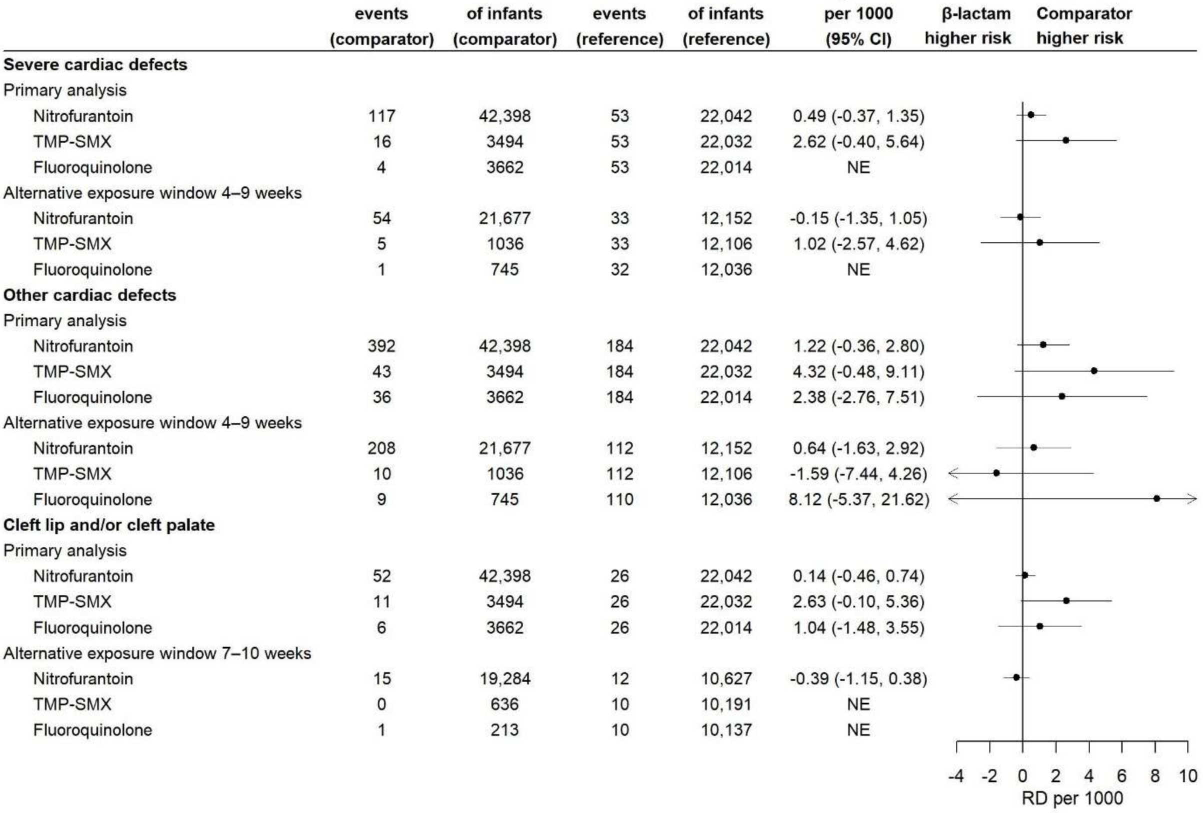

Abbreviations: CI, confidence interval; NE, not estimable; RD, risk difference; TMP-SMX, trimethoprim-sulfamethoxazole.

[a] Propensity score weighted analysis accounted for all potential confounders listed in eTable 8. We required ≥50 events in the UTI cohort to perform specific malformation group analyses. We required ≥5 events in each exposure group to estimate the treatment effect.

© 2025 Osmundson SS et al. *JAMA Network Open*.

eFigure 11. Absolute Risk of Congenital Malformations Among Infants Born to Individuals Treated with a UTI-related Antibiotic for Any Indication in the First Trimester by Index Antibiotic Agent and Indication[a,b]

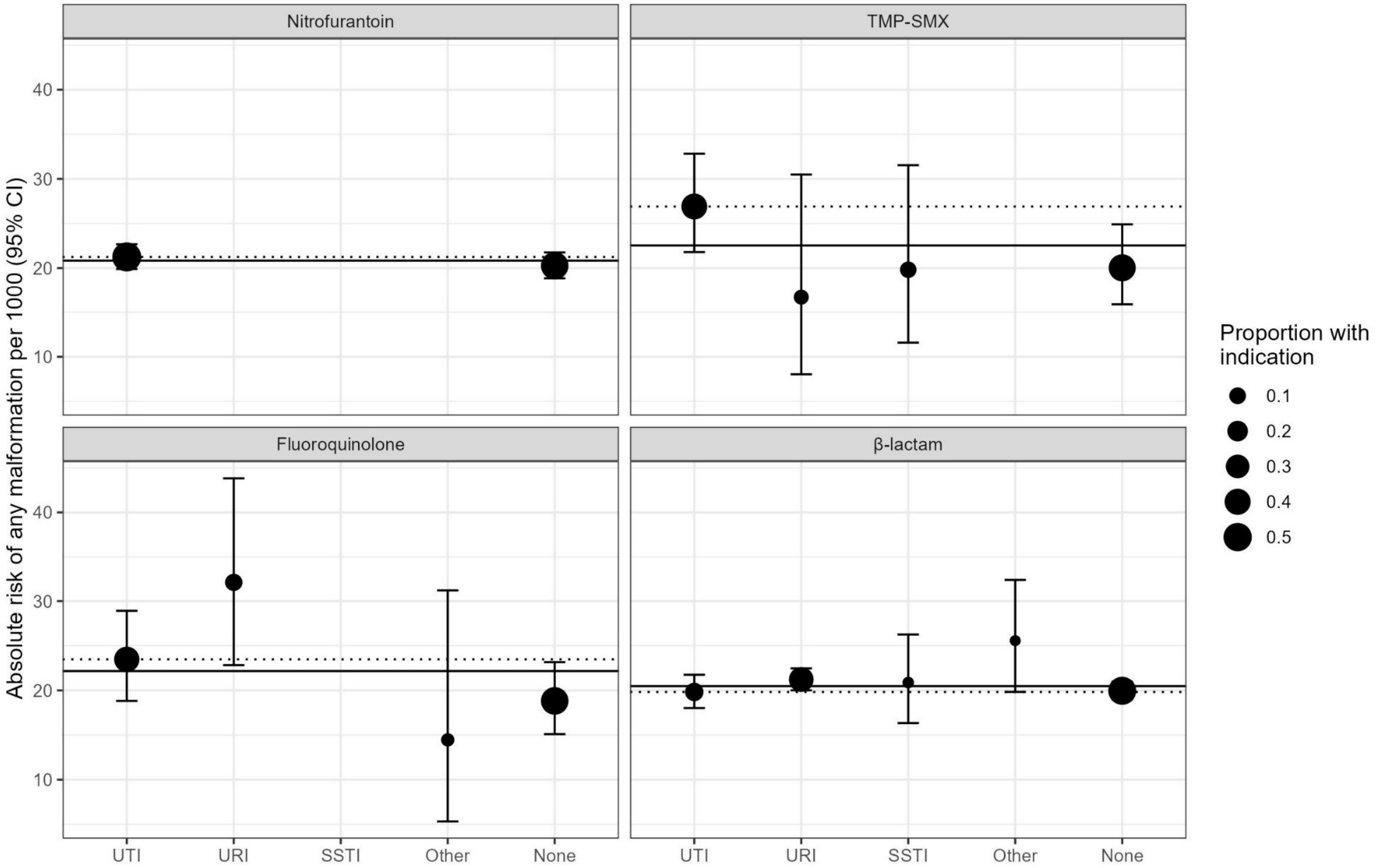

Abbreviations: CI, confidence interval; SSTI, skin and soft tissue infection; TMP-SMX, trimethoprim-sulfamethoxazole; URI, upper respiratory infection; UTI, urinary tract infection.

© 2025 Osmundson SS et al. *JAMA Network Open*.

[a] Indications for the antibiotic included UTI, URI, SSTI, other infection (i.e., abdominal, dental, lower respiratory, pyelonephritis infection, inpatient UTI), and none of the aforementioned infections.
[b] The solid horizontal line shows the absolute risk of any malformation, pooling all indications within each antibiotic exposure group. The dotted horizontal line shows the absolute risk of any malformation for pregnancies with a UTI indication within each antibiotic exposure group. Estimates for indications representing less than 1% of an antibiotic exposure group are not shown. The circles for point estimates are proportional to the number of pregnancies with the given indication within each antibiotic agent.

© 2025 Osmundson SS et al. *JAMA Network Open*.

eFigure 12. Weighted Risk Difference Estimates of Any Congenital Malformation in Infants Born to Individuals Treated with a UTI-related Antibiotic for Any Indication in the First Trimester: Comparison of Different UTI-related Antibiotics Versus β-lactams (Reference)[a,b]

	No. of events (comparator)	Total no. of infants (comparator)	No. of events (reference)	Total no. of infants (reference)	Weighted RD per 1000 (95% CI)
**Primary analysis**					
Nitrofurantoin	1663	79,826	3234	157,915	0.92 (-0.35, 2.19)
TMP-SMX	202	8962	3232	157,757	2.76 (-0.66, 6.19)
Fluoroquinolone	221	9970	3234	157,884	0.63 (-2.58, 3.84)
**Sensitivity analysis**					
Alternative reference group					
Nitrofurantoin	1663	79,820	2099	103,122	0.99 (-0.38, 2.36)
TMP-SMX	202	8961	2099	103,104	3.05 (-0.44, 6.55)
Fluoroquinolone	221	9970	2099	103,097	0.74 (-2.55, 4.02)
Alternative outcome definition					
Nitrofurantoin	2533	79,826	4958	157,915	0.91 (-0.64, 2.46)
TMP-SMX	309	8962	4952	157,757	3.35 (-0.77, 7.47)
Fluoroquinolone	346	9970	4958	157,884	0.99 (-2.88, 4.85)
Restricted to ICD-10 era					
Nitrofurantoin	612	26,735	1182	58,068	2.91 (0.71, 5.12)
TMP-SMX	63	2358	1180	57,940	5.93 (-0.83, 12.69)
Fluoroquinolone	44	2224	1172	57,823	-1.70 (-7.82, 4.42)
Alternative exposure window 2–13 weeks					
Nitrofurantoin	1593	76,466	2855	139,626	0.90 (-0.41, 2.21)
TMP-SMX	141	6458	2855	139,606	2.05 (-1.97, 6.08)
Fluoroquinolone	143	6107	2855	139,592	1.62 (-2.50, 5.74)
Adjusted for gestational age at exposure					
Nitrofurantoin	1663	79,827	3234	157,909	1.12 (-0.21, 2.45)
TMP-SMX	202	8960	3234	157,897	2.92 (-0.96, 6.80)
Fluoroquinolone	221	9970	3233	157,863	5.29 (-1.93, 12.51)

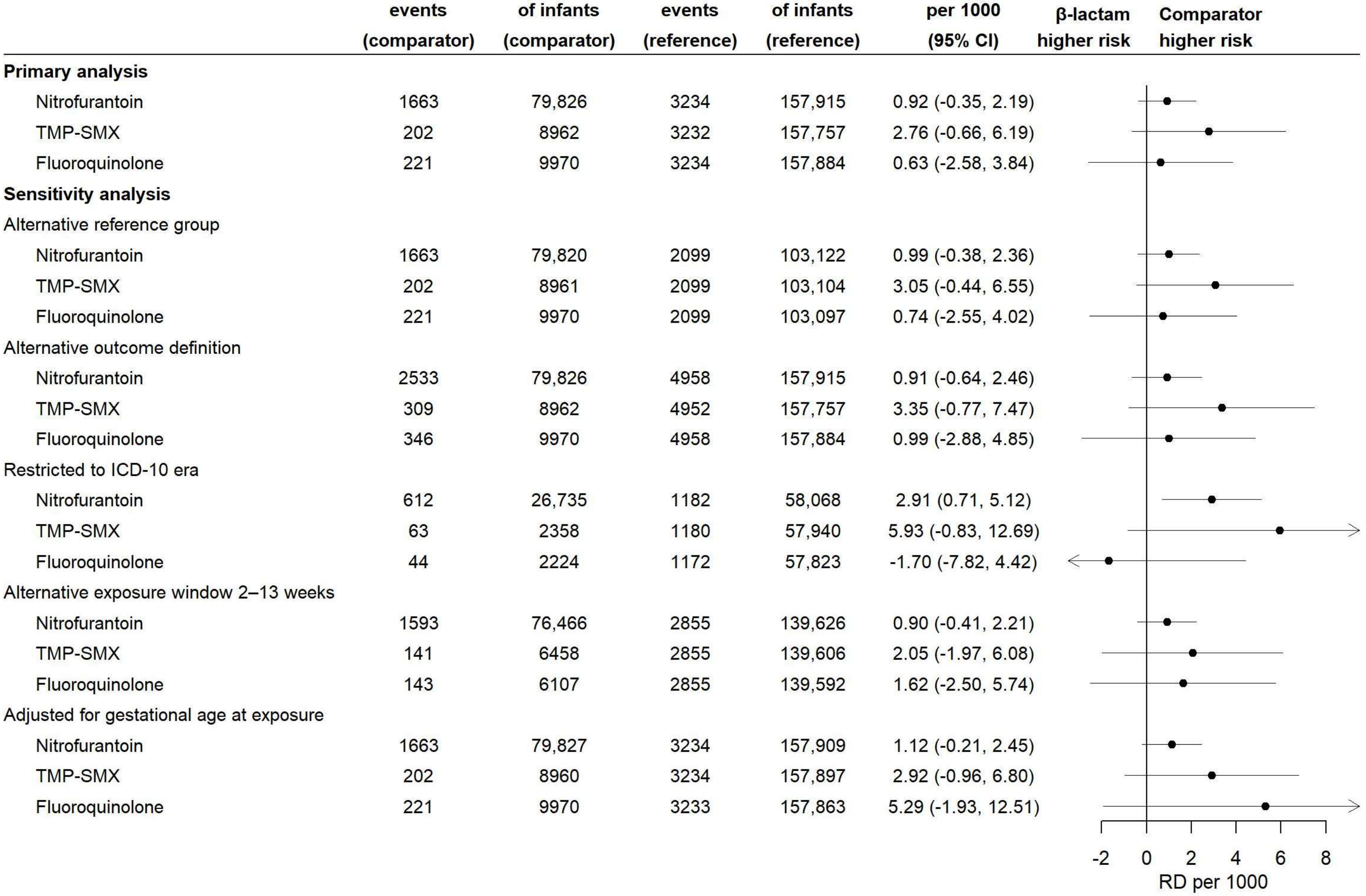

© 2025 Osmundson SS et al. *JAMA Network Open*.

Abbreviations: CI, confidence interval; ICD, International Classification of Diseases; RD, risk difference; TMP-SMX, trimethoprim-sulfamethoxazole; UTI, urinary tract infection.

[a] Sensitivity analysis alternative reference group subsetted to amoxicillin alone or cephalexin (rather than all UTI-related β-lactam antibiotics). Sensitivity analysis alternative outcome definition relaxed the congenital malformation algorithm to allow any single diagnosis code within the specific malformation group (after excluding diagnostic/rule-out codes). Sensitivity analysis symptomatic UTI restricted to symptomatic UTIs (rather than including asymptomatic bacteriuria); symptomatic UTI defined as meeting ≥1 of the following criteria: urine culture on the same date as the antibiotic; no urine culture ± 7 days of the antibiotic; a UTI symptom diagnosis code ± 7 days of the antibiotic. Sensitivity analysis ICD-10 era restricted to pregnancies with delivery dates on or after October 1, 2015. Sensitivity analysis alternative exposure window restricted the antibiotic exposure timeframe from 0-13 weeks to 2-13 weeks gestation (14-97 days of gestation). Sensitivity analysis adjusted for gestational age at exposure using gestational age distribution tertiles (0-6 weeks, 7-9 weeks, 10-13 weeks).
[b] Propensity score weighted analysis accounted for all potential confounders listed in eTable 8. We required ≥5 events in each exposure group to estimate the treatment effect.

© 2025 Osmundson SS et al. *JAMA Network Open*.

eFigure 13. Weighted Risk Ratio Estimates of Any Congenital Malformation in Infants Born to Individuals Treated with a UTI-related Antibiotic for Any Indication in the First Trimester: Comparison of Different UTI-related Antibiotics Versus β-lactams (Reference)[a,b]

	No. of events (comparator)	Total no. of infants (comparator)	No. of events (reference)	Total no. of infants (reference)	Weighted RR (95% CI)
**Primary analysis**					
Nitrofurantoin	1663	79,826	3234	157,915	1.04 (0.98, 1.11)
TMP-SMX	202	8962	3232	157,757	1.13 (0.98, 1.32)
Fluoroquinolone	221	9970	3234	157,884	1.03 (0.89, 1.20)
**Sensitivity analysis**					
Alternative reference group					
Nitrofurantoin	1663	79,820	2099	103,122	1.05 (0.98, 1.12)
TMP-SMX	202	8961	2099	103,104	1.15 (0.99, 1.34)
Fluoroquinolone	221	9970	2099	103,097	1.04 (0.89, 1.21)
Alternative outcome definition					
Nitrofurantoin	2533	79,826	4958	157,915	1.03 (0.98, 1.08)
TMP-SMX	309	8962	4952	157,757	1.11 (0.98, 1.25)
Fluoroquinolone	346	9970	4958	157,884	1.03 (0.92, 1.16)
Restricted to ICD-10 era					
Nitrofurantoin	612	26,735	1182	58,068	1.14 (1.04, 1.26)
TMP-SMX	63	2358	1180	57,940	1.29 (1.00, 1.67)
Fluoroquinolone	44	2224	1172	57,823	0.92 (0.66, 1.27)
Alternative exposure window 2–13 weeks					
Nitrofurantoin	1593	76,466	2855	139,626	1.04 (0.98, 1.11)
TMP-SMX	141	6458	2855	139,606	1.10 (0.92, 1.32)
Fluoroquinolone	143	6107	2855	139,592	1.08 (0.89, 1.30)
Adjusting for gestational age at exposure					
Nitrofurantoin	1663	79,827	3234	157,909	1.05 (0.99, 1.12)
TMP-SMX	202	8960	3234	157,897	1.14 (0.97, 1.35)
Fluoroquinolone	221	9970	3233	157,863	1.26 (0.95, 1.67)

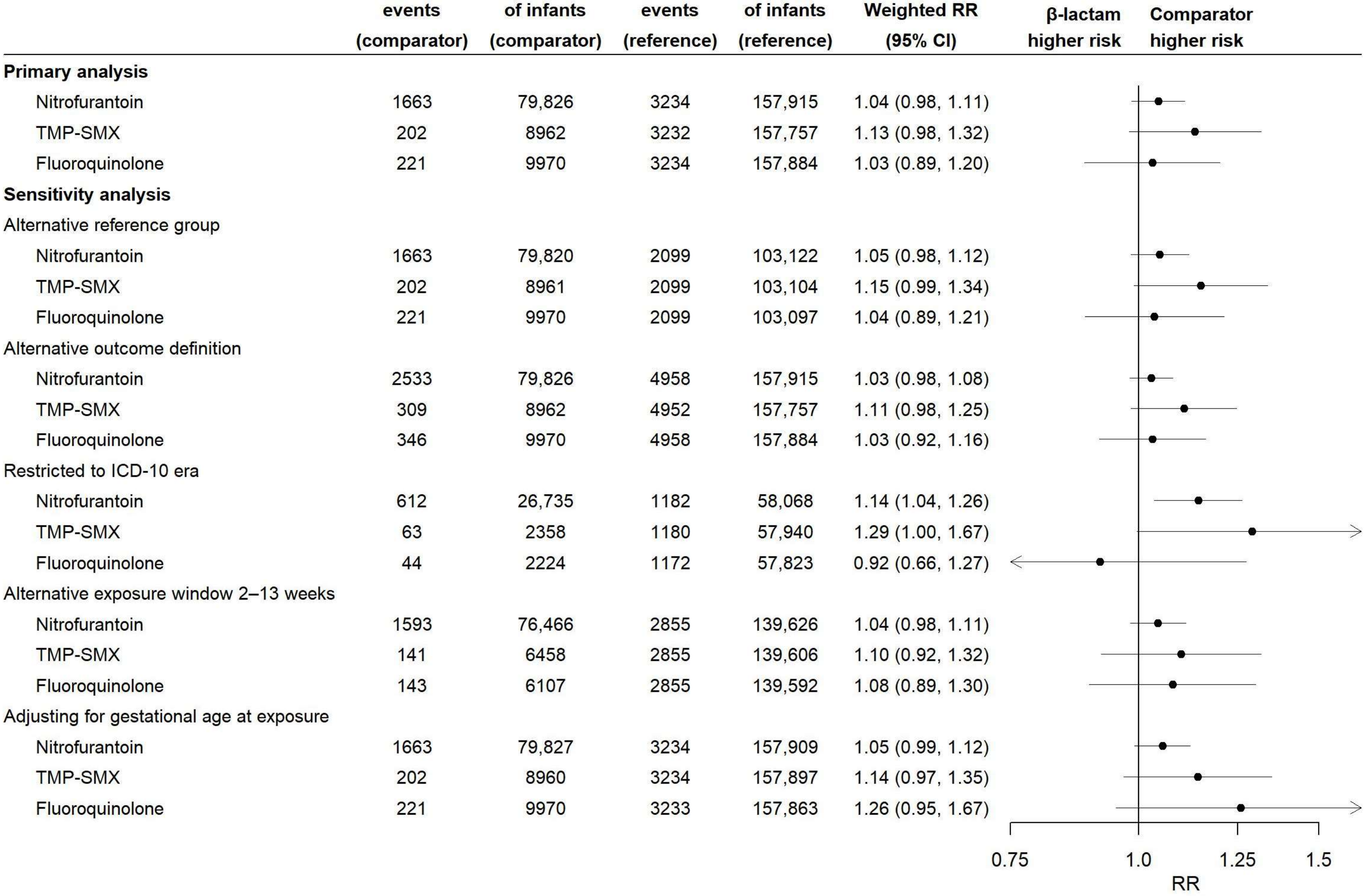

© 2025 Osmundson SS et al. *JAMA Network Open*.

Abbreviations: CI, confidence interval; ICD, International Classification of Diseases; RR, risk ratio; TMP-SMX, trimethoprim-sulfamethoxazole; UTI, urinary tract infection.

[a] Sensitivity analysis alternative reference group subsetted to amoxicillin alone or cephalexin (rather than all UTI-related β-lactam antibiotics). Sensitivity analysis alternative outcome definition relaxed the congenital malformation algorithm to allow any single diagnosis code within the specific malformation group (after excluding diagnostic/rule-out codes). Sensitivity analysis symptomatic UTI restricted to symptomatic UTIs (rather than including asymptomatic bacteriuria); symptomatic UTI defined as meeting ≥1 of the following criteria: urine culture on the same date as the antibiotic; no urine culture ± 7 days of the antibiotic; a UTI symptom diagnosis code ± 7 days of the antibiotic. Sensitivity analysis ICD-10 era restricted to pregnancies with delivery dates on or after October 1, 2015. Sensitivity analysis alternative exposure window restricted the antibiotic exposure timeframe from 0-13 weeks to 2-13 weeks gestation (14-97 days of gestation). Sensitivity analysis adjusted for gestational age at exposure using gestational age distribution tertiles (0-6 weeks, 7-9 weeks, 10-13 weeks).
[b] Propensity score weighted analysis accounted for all potential confounders listed in eTable 8. We required ≥5 events in each exposure group to estimate the treatment effect.

© 2025 Osmundson SS et al. *JAMA Network Open*.

eFigure 14. Weighted Risk Difference Estimates of Any Cardiac Malformation in Infants Born to Individuals Treated with a UTI-related Antibiotic for Any Indication in the First Trimester: Comparison of Different UTI-related Antibiotics Versus β-lactams (Reference)[a,b]

	No. of events (comparator)	Total no. of infants (comparator)	No. of events (reference)	Total no. of infants (reference)	Weighted RD per 1000 (95% CI)
**Primary analysis**					
Nitrofurantoin	794	79,826	1505	157,915	0.79 (-0.10, 1.68)
TMP-SMX	97	8962	1504	157,757	1.77 (-0.65, 4.19)
Fluoroquinolone	98	9970	1505	157,884	0.61 (-1.75, 2.97)
**Sensitivity analysis**					
Alternative reference group					
Nitrofurantoin	794	79,820	976	103,122	0.80 (-0.15, 1.75)
TMP-SMX	97	8961	976	103,104	1.98 (-0.49, 4.46)
Fluoroquinolone	98	9970	976	103,097	0.66 (-1.76, 3.08)
Alternative outcome definition					
Nitrofurantoin	1174	79,826	2235	157,915	0.96 (-0.11, 2.03)
TMP-SMX	138	8962	2233	157,757	1.32 (-1.45, 4.09)
Fluoroquinolone	144	9970	2235	157,884	0.05 (-2.63, 2.72)
Restricted to ICD-10 era					
Nitrofurantoin	285	26,735	588	58,068	0.99 (-0.56, 2.55)
TMP-SMX	31	2358	587	57,940	2.54 (-2.14, 7.22)
Fluoroquinolone	25	2224	583	57,823	1.45 (-3.46, 6.36)
Alternative exposure window 4–9 weeks					
Nitrofurantoin	388	38,205	694	70,770	0.71 (-0.62, 2.03)
TMP-SMX	21	2532	694	70,743	-2.31 (-5.83, 1.22)
Fluoroquinolone	31	2182	692	70,579	5.93 (-0.83, 12.68)
Adjusted for gestational age at exposure					
Nitrofurantoin	794	79,827	1505	157,909	0.80 (-0.12, 1.72)
TMP-SMX	97	8960	1505	157,897	2.24 (-0.59, 5.07)
Fluoroquinolone	98	9970	1504	157,863	5.95 (-0.24, 12.14)

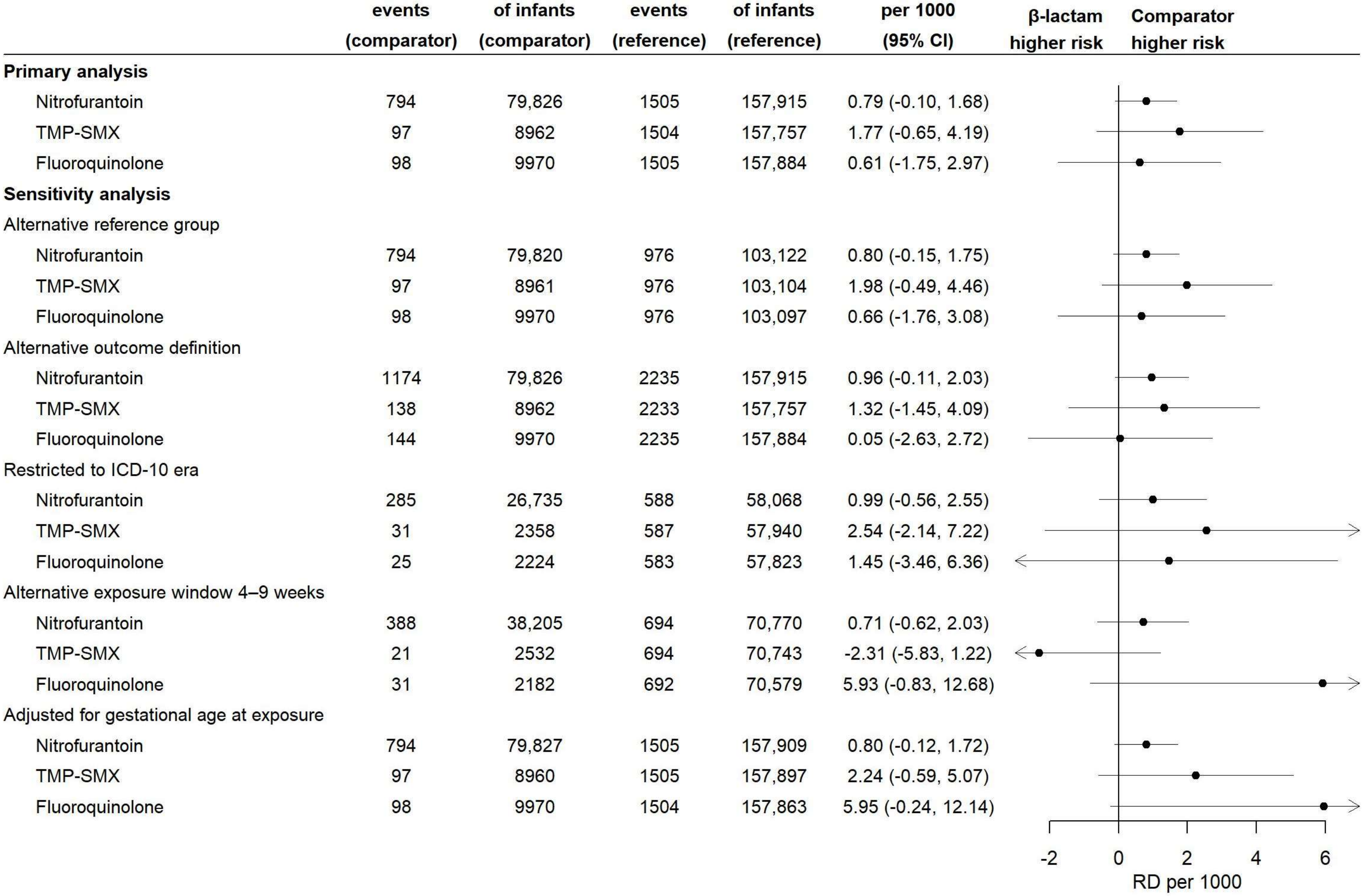

© 2025 Osmundson SS et al. *JAMA Network Open*.

Abbreviations: CI, confidence interval; ICD, International Classification of Diseases; RD, risk difference; TMP-SMX, trimethoprim-sulfamethoxazole; UTI, urinary tract infection.

[a] Sensitivity analysis alternative reference group subsetted to amoxicillin alone or cephalexin (rather than all UTI-related β-lactam antibiotics). Sensitivity analysis alternative outcome definition relaxed the congenital malformation algorithm to allow any single diagnosis code within the specific malformation group (after excluding diagnostic/rule-out codes). Sensitivity analysis symptomatic UTI restricted to symptomatic UTIs (rather than including asymptomatic bacteriuria); symptomatic UTI defined as meeting ≥1 of the following criteria: urine culture on the same date as the antibiotic; no urine culture ± 7 days of the antibiotic; a UTI symptom diagnosis code ± 7 days of the antibiotic. Sensitivity analysis ICD-10 era restricted to pregnancies with delivery dates on or after October 1, 2015. Sensitivity analysis alternative exposure window restricted the antibiotic exposure timeframe from 0-13 weeks to 4-9 weeks gestation (28-69 days of gestation). Sensitivity analysis adjusted for gestational age at exposure using gestational age distribution tertiles (0-6 weeks, 7-9 weeks, 10-13 weeks).
[b] Propensity score weighted analysis accounted for all potential confounders listed in eTable 8. We required ≥5 events in each exposure group to estimate the treatment effect.

© 2025 Osmundson SS et al. *JAMA Network Open*.

eFigure 15. Weighted Risk Ratio Estimates of Any Cardiac Malformation in Infants Born to Individuals Treated with a UTI-related Antibiotic for Any Indication in the First Trimester: Comparison of Different UTI-related Antibiotics Versus β-lactams (Reference)[a,b]

	No. of events (comparator)	Total no. of infants (comparator)	No. of events (reference)	Total no. of infants (reference)	Weighted RR (95% CI)
**Primary analysis**					
Nitrofurantoin	794	79,826	1505	157,915	1.08 (0.99, 1.18)
TMP-SMX	97	8962	1504	157,757	1.19 (0.96, 1.47)
Fluoroquinolone	98	9970	1505	157,884	1.06 (0.84, 1.34)
**Sensitivity analysis**					
Alternative reference group					
Nitrofurantoin	794	79,820	976	103,122	1.08 (0.99, 1.19)
TMP-SMX	97	8961	976	103,104	1.21 (0.97, 1.51)
Fluoroquinolone	98	9970	976	103,097	1.07 (0.84, 1.36)
Alternative outcome definition					
Nitrofurantoin	1174	79,826	2235	157,915	1.07 (0.99, 1.15)
TMP-SMX	138	8962	2233	157,757	1.09 (0.91, 1.31)
Fluoroquinolone	144	9970	2235	157,884	1.00 (0.83, 1.21)
Restricted to ICD-10 era					
Nitrofurantoin	285	26,735	588	58,068	1.10 (0.95, 1.27)
TMP-SMX	31	2358	587	57,940	1.25 (0.86, 1.82)
Fluoroquinolone	25	2224	583	57,823	1.14 (0.75, 1.75)
Alternative exposure window 4–9 weeks					
Nitrofurantoin	388	38,205	694	70,770	1.07 (0.94, 1.22)
TMP-SMX	21	2532	694	70,743	0.76 (0.48, 1.22)
Fluoroquinolone	31	2182	692	70,579	1.60 (1.04, 2.47)
Adjusted for gestational age at exposure					
Nitrofurantoin	794	79,827	1505	157,909	1.08 (0.99, 1.19)
TMP-SMX	97	8960	1505	157,897	1.24 (0.97, 1.57)
Fluoroquinolone	98	9970	1504	157,863	1.62 (1.09, 2.43)

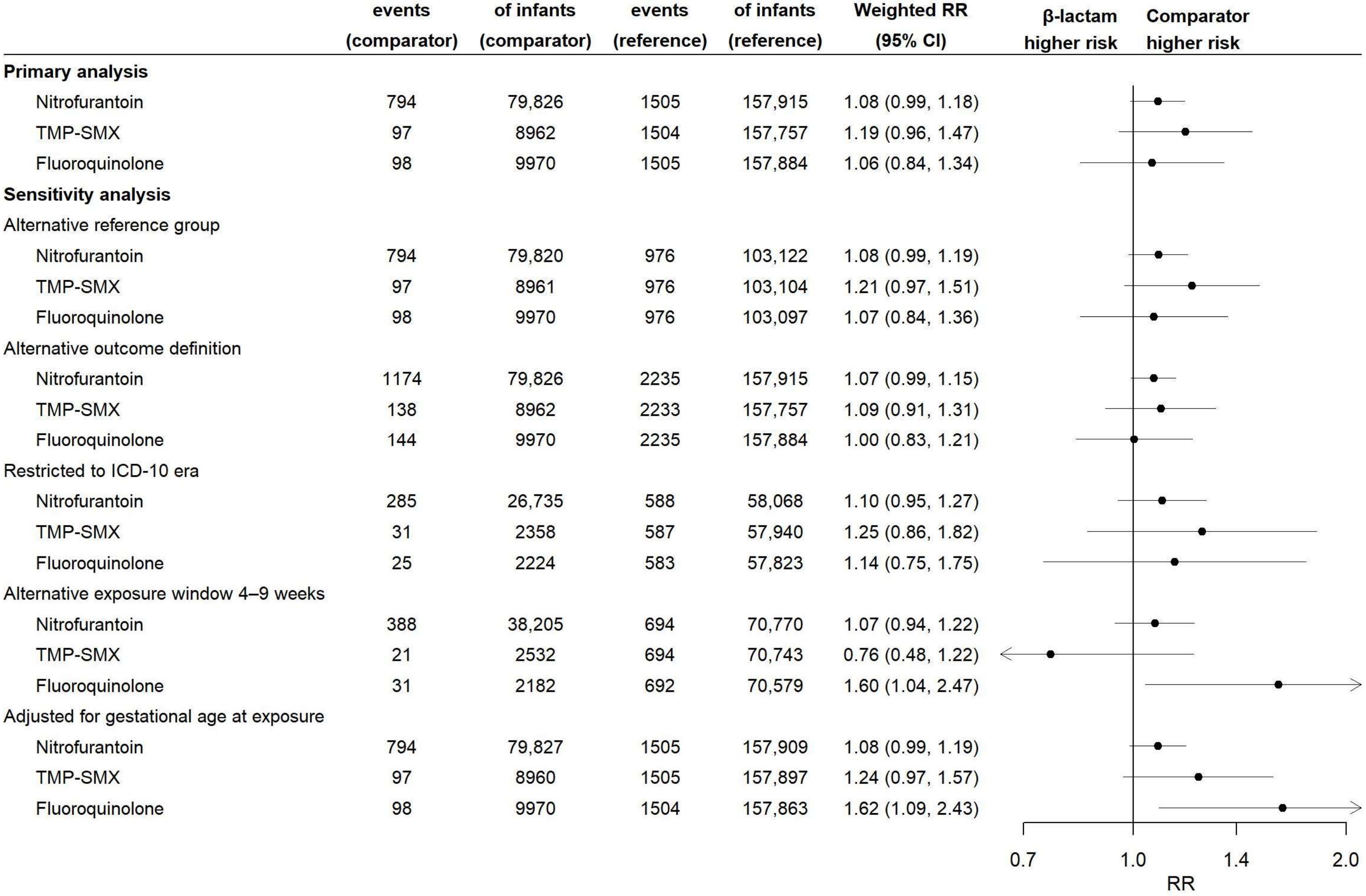

© 2025 Osmundson SS et al. *JAMA Network Open*.

Abbreviations: CI, confidence interval; ICD, International Classification of Diseases; RR, risk ratio; TMP-SMX, trimethoprim-sulfamethoxazole; UTI, urinary tract infection.

[a] Sensitivity analysis alternative reference group subsetted to amoxicillin alone or cephalexin (rather than all UTI-related β-lactam antibiotics). Sensitivity analysis alternative outcome definition relaxed the congenital malformation algorithm to allow any single diagnosis code within the specific malformation group (after excluding diagnostic/rule-out codes). Sensitivity analysis symptomatic UTI restricted to symptomatic UTIs (rather than including asymptomatic bacteriuria); symptomatic UTI defined as meeting ≥1 of the following criteria: urine culture on the same date as the antibiotic; no urine culture ± 7 days of the antibiotic; a UTI symptom diagnosis code ± 7 days of the antibiotic. Sensitivity analysis ICD-10 era restricted to pregnancies with delivery dates on or after October 1, 2015. Sensitivity analysis alternative exposure window restricted the antibiotic exposure timeframe from 0-13 weeks to 4-9 weeks gestation (28-69 days of gestation). Sensitivity analysis adjusted for gestational age at exposure using gestational age distribution tertiles (0-6 weeks, 7-9 weeks, 10-13 weeks).
[b] Propensity score weighted analysis accounted for all potential confounders listed in eTable 8. We required ≥5 events in each exposure group to estimate the treatment effect.

© 2025 Osmundson SS et al. *JAMA Network Open*.

eAppendix.

Quantitative bias analysis of the potential impact of selection bias due to restriction of the study cohort to livebirths

It is possible that restriction of the study population to livebirths could induce selection bias if the probability of livebirth varies by antibiotic agent and malformation outcome status.[6] Following approaches described in Huybrechts et al, we quantified the potential impact of restricting our cohort to livebirths by calculating a corrected risk ratio (RR)[7,8] as follows:

Corrected RR = observed RR * (S_{10}*S_{01} / S_{11}*S_{00})

Where:

- S_{00} is Pr(livebirth | no malformation, β-lactam exposure)
- S_{01} is Pr(livebirth | no malformation, comparator antibiotic exposure)
- S_{10} is Pr(livebirth | malformation, β-lactam exposure)
- S_{11} is Pr(livebirth | malformation, comparator antibiotic exposure)

We considered two approaches to these sensitivity analyses to explore the potential impact of selection bias and applied them to the observed RR comparing first-trimester pregnancy exposure to TMP-SMX vs. β-lactams and to fluoroquinolones vs. β-lactams. The first approach estimated the proportion of live births in each of the exposure groups and assumed that these proportions were equal to the baseline probability of livebirth in the absence of malformation. As β-lactams were assumed to be the safest of the antibiotics under study, we used the estimated proportion of that group as S_{00} and the estimated proportion in the comparator group as S_{01}. We calculated corrected RR allowing the probability in the comparator group to vary further and closer to the probability of the β-lactam group. The second approach used the same estimated proportions but switched the proportions of the β-lactam and comparator groups, thereby reversing the effect of selection bias due to restriction of the cohort to livebirths. In both approaches, we hypothesized a range of possible values for the decrease in the probability of livebirth in the presence of a malformation, which were used to calculate S_{10} and S_{11}.

To estimate the proportion of livebirth by exposure group, we identified all pregnant individuals who met our cohort criteria but did not apply the requirement for a linked livebirth. Specifically, we identified all pregnant individuals 15-49 years with first trimester UTI-related oral antibiotic exposure to a single UTI-related antibiotic agent with a UTI indication for the antibiotic and required insurance coverage from 90 days before LMP through 30 days after delivery. We then calculated the proportion of individuals among this cohort with a livebirth in each exposure group. The observed proportion of livebirth was 83.0% in β-lactam-exposed pregnancies, 71.4% in TMP-SMX-exposed pregnancies, 64.8% in fluoroquinolone-exposed pregnancies, and 82.9% in nitrofurantoin-exposed pregnancies. Because the observed proportions of livebirth in β-lactams and nitrofurantoin were nearly identical, we did not include this comparison in subsequent analyses. We examined scenarios ranging from none (i.e., $S_{01} = S_{00}$) to all of the difference in observed proportions between the β-lactam group and the comparator group being due to selection bias. Furthermore, we varied the probabilities of livebirth among those with a malformation (S_{11} and S_{10}) by assuming they were equal to the probabilities in the absence of a malformation (S_{01} and S_{00}) minus a fixed probability of between 5% and 20%; this absolute decrease in the probability of livebirth due to malformation was applied equally to the β-lactam group and the comparator group (i.e., $S_{00} - S_{10} = S_{01} - S_{11}$). In our second approach, we examined scenarios where selection bias was of the same magnitude but acted in the opposite direction, i.e., that the β-lactam group had a lower probability of livebirth than the comparator group.

We present plots of corrected RR of any malformation and any cardiac malformation for comparisons of TMP-SMX vs. β-lactams (eFigure 16) and fluoroquinolones vs. β-lactams (eFigure 17), with accompanying example calculations used in these sensitivity analyses for the TMP-SMX vs. β-lactam comparison (eTable 22). In our first approach, under any scenario where the β-lactam group was assumed to have a higher probability of livebirth than the comparator group, the presence of any selection bias shifted the RR away from the null; this effect became more pronounced as the magnitude

© 2025 Osmundson SS et al. *JAMA Network Open*.

of the decrease in the probability of livebirth in the presence of a malformation increased. In the most extreme scenario (100% selection bias and 20% absolute decrease in probability of livebirth with a malformation), the RR estimate for the TMP-SMX vs. β-lactam comparison shifted from 1.35 to 1.42 for any malformation and from 1.45 to 1.53 for any cardiac malformation (eFigure 16). For the same extreme scenario, the RR estimate for the fluoroquinolone vs. β-lactam comparison shifted from 1.18 to 1.30 for any malformation and from 1.23 to 1.35 for any cardiac malformation (eFigure 17).

In our second approach, we examined scenarios where selection bias was of the same magnitude but acted in the opposite direction, i.e., that β-lactam had a lower probability of livebirth than the comparator antibiotic. Under these conditions, selection bias shifted the RR estimates towards the null. The most extreme scenario assumed 100% selection bias and a 20% absolute decrease in the probability of livebirths associated with a malformation and shifted the RR estimate for the TMP-SMX vs. β-lactam comparison from 1.35 to 1.28 for any malformation and from 1.45 to 1.37 for any cardiac malformation (eFigure 16). For the same extreme scenario, the RR estimate for the fluoroquinolone vs. β-lactam comparison shifted from 1.18 to 1.08 for any malformation and from 1.23 to 1.12 for any cardiac malformation (eFigure 17).

In conclusion, based on observed proportions of livebirth in our data, if selection bias due to the restriction of the cohort to livebirths is present, then our observed RR are underestimates of the true association between comparator antibiotics versus β-lactam and congenital malformations. Under alternative assumptions where the probability of non-livebirths is lower in the comparator-exposed pregnancies compared to β-lactam-exposed pregnancies, then our observed RR are overestimates. However, the RR estimate under the most extreme assumptions remains >1.25 for TMP-SMX versus β-lactam, the comparison for which we had significant findings in the primary analysis. Therefore, our study conclusions are unlikely to meaningfully change based on selection bias due to restricting the cohort to livebirths.

eTable 22. Values of the Probability of Livebirth for β-lactam and Trimethoprim-sulfamethoxazole Used in Sensitivity Analyses and Corrected Risk Ratios for Any Malformation*

Proportion of difference due to selection bias	Decrease in livebirth probability in malformation group	Probability of livebirth among pregnancies with: No malformation			Malformation			Corrected risk ratio for any malformation
		TMP-SMX (S_{01})	β-lactam (S_{00})	Ratio (S_{01} / S_{00})	TMP-SMX (S_{11})	β-lactam (S_{10})	Ratio (S_{10} / S_{11})	
Approach 1								
0	5	83.0	83.0	1.00	78.0	78.0	1.00	1.35
0	10	83.0	83.0	1.00	73.0	73.0	1.00	1.35
0	20	83.0	83.0	1.00	63.0	63.0	1.00	1.35
50	5	77.2	83.0	0.93	72.2	78.0	1.08	1.35
50	10	77.2	83.0	0.93	67.2	73.0	1.09	1.36
50	20	77.2	83.0	0.93	57.2	63.0	1.10	1.38
100	5	71.4	83.0	0.86	66.4	78.0	1.17	1.36
100	10	71.4	83.0	0.86	61.4	73.0	1.19	1.38
100	20	71.4	83.0	0.86	51.4	63.0	1.23	1.42
Approach 2								
0	5	83.0	83.0	1.00	78.0	78.0	1.00	1.35
0	10	83.0	83.0	1.00	73.0	73.0	1.00	1.35

0	20	83.0	83.0	1.00	63.0	63.0	1.00	1.35
50	5	83.0	77.2	1.07	78.0	72.2	0.93	1.34
50	10	83.0	77.2	1.07	73.0	67.2	0.92	1.33
50	20	83.0	77.2	1.07	63.0	57.2	0.91	1.32
100	5	83.0	71.4	1.16	78.0	66.4	0.85	1.33
100	10	83.0	71.4	1.16	73.0	61.4	0.84	1.32
100	20	83.0	71.4	1.16	63.0	51.4	0.82	1.28

*observed RR = 1.35 comparing risk of any malformation for those pregnancies exposed to TMP-SMX compared to β-lactam

eFigure 16. Selection Bias-Corrected Corrected Weighted Risk Ratio Estimates of (A) Any Malformation and (B) Any Cardiac Malformation in Infants Born to Individuals Treated for Urinary Tract Infection (UTI) in the First Trimester: TMP-SMX Versus β-lactam (Reference)[a]

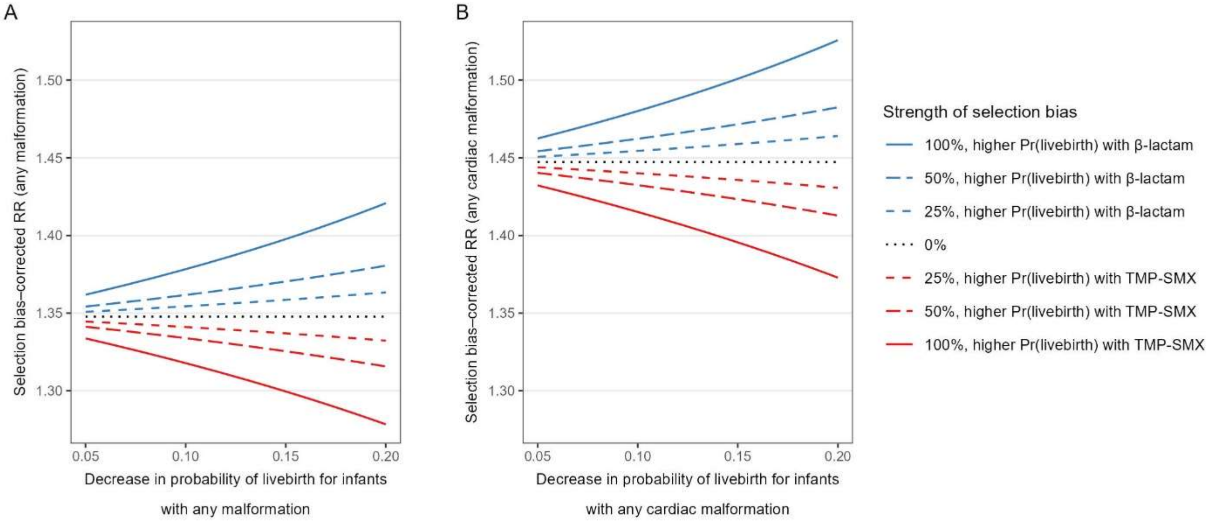

Abbreviations: RR, risk ratio; TMP-SMX, trimethoprim-sulfamethoxazole.

[a] Propensity score weighted estimates accounted for all potential confounders listed in eTable 8. Starting observed RR for any malformation was 1.35; starting observed RR for any cardiac malformation was 1.45.

eFigure 17. Selection Bias-Corrected Weighted Risk Ratio Estimates of (A) Any Malformation and (B) Any Cardiac Malformation in Infants Born to Individuals Treated for Urinary Tract Infection (UTI) in the First Trimester: Fluoroquinolone Versus β-lactam (Reference)[a]

© 2025 Osmundson SS et al. *JAMA Network Open*.

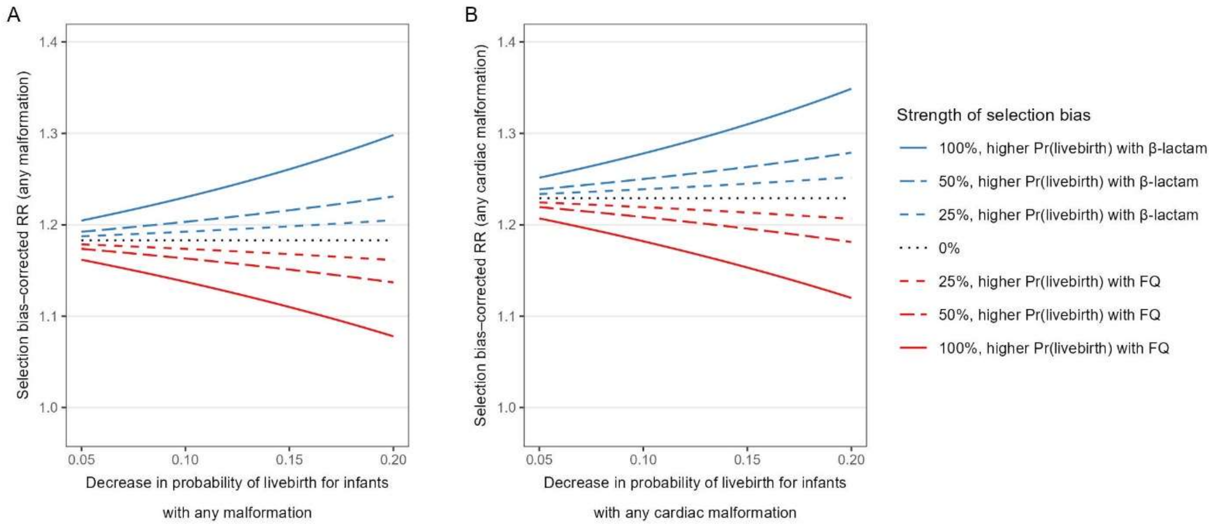

Abbreviations: FQ, fluoroquinolone; RR, risk ratio.

[a] Propensity score weighted estimates accounted for all potential confounders listed in eTable 8. Starting observed RR for any malformation was 1.18; starting observed RR for any cardiac malformation was 1.23.

© 2025 Osmundson SS et al. *JAMA Network Open*.
