# Supplementary material for: First-Trimester Antibiotic Use for Urinary Tract Infection and Risk of Congenital Malformations
Source: JAMA Netw Open. 2025 Jul 9;8(7):e2519544. doi: 10.1001/jamanetworkopen.2025.19544 (PMC12242685; doi:10.1001/jamanetworkopen.2025.19544)
Supplement: Supplement 3. — Data Sharing Statement [file jamanetwopen-e2519544-s003.pdf]

Data Sharing Statement

Osmundson. First-Trimester Antibiotic Use for Urinary Tract Infection and Risk of Congenital Malformations. *JAMA Netw Open*. Published July 09, 2025.
doi:10.1001/jamanetworkopen.2025.19544

Data
Data available: No

Additional Information
Explanation for why data not available: Available for license through Merative.
